# A photoacoustic patch for three-dimensional imaging of hemoglobin and core temperature

**DOI:** 10.1038/s41467-022-35455-3

**Published:** 2022-12-15

**Authors:** Xiaoxiang Gao, Xiangjun Chen, Hongjie Hu, Xinyu Wang, Wentong Yue, Jing Mu, Zhiyuan Lou, Ruiqi Zhang, Keren Shi, Xue Chen, Muyang Lin, Baiyan Qi, Sai Zhou, Chengchangfeng Lu, Yue Gu, Xinyi Yang, Hong Ding, Yangzhi Zhu, Hao Huang, Yuxiang Ma, Mohan Li, Aditya Mishra, Joseph Wang, Sheng Xu

**Affiliations:** 1 Department of Nanoengineering, University of California San Diego, La Jolla, CA 92093 USA; 2 Materials Science and Engineering Program, University of California San Diego, La Jolla, CA 92093 USA; 3 Department of Electrical and Computer Engineering, University of California San Diego, La Jolla, CA USA; 4 Department of Radiology, School of Medicine, University of California San Diego, La Jolla, CA 92093 USA; 5 Department of Bioengineering, University of California San Diego, La Jolla, 92093 CA USA

**Keywords:** Sensors and biosensors, Ultrasonography, Photoacoustics, Imaging techniques

## Abstract

Electronic patches, based on various mechanisms, allow continuous and noninvasive monitoring of biomolecules on the skin surface. However, to date, such devices are unable to sense biomolecules in deep tissues, which have a stronger and faster correlation with the human physiological status than those on the skin surface. Here, we demonstrate a photoacoustic patch for three-dimensional (3D) mapping of hemoglobin in deep tissues. This photoacoustic patch integrates an array of ultrasonic transducers and vertical-cavity surface-emitting laser (VCSEL) diodes on a common soft substrate. The high-power VCSEL diodes can generate laser pulses that penetrate >2 cm into biological tissues and activate hemoglobin molecules to generate acoustic waves, which can be collected by the transducers for 3D imaging of the hemoglobin with a high spatial resolution. Additionally, the photoacoustic signal amplitude and temperature have a linear relationship, which allows 3D mapping of core temperatures with high accuracy and fast response. With access to biomolecules in deep tissues, this technology adds unprecedented capabilities to wearable electronics and thus holds significant implications for various applications in both basic research and clinical practice.

## Abstract (web summary)

The authors present a wearable photoacoustic patch, which integrates laser diodes and piezoelectric transducers for three-dimensional imaging of hemoglobin and temperature in deep tissues.

## Introduction

Monitoring biomolecules in the human body can help track wellness levels, diagnose diseases, and evaluate therapeutic outcomes. In particular, the amount and location of hemoglobin in the body provide critical information about blood perfusion or accumulation in that area. Low blood perfusion inside the body may result in severe organ dysfunctions. It can happen in many kinds of diseases (such as myocardial infarction^1^, post‐cardiac arrest syndrome^2^, and vascular diseases of the extremities^3^), or after surgery (such as organ transplant^4^). On the contrary, accumulation of blood is often a sign of inflammation^5^, trauma^6^, or cancer^7^. For example, cysts with many possible types of biofluids inside may be found throughout the human body. Bloody cysts are suspicious and should be further examined and closely monitored for the risk of malignant tumors^8,9^. Continuous monitoring can benefit understanding and diagnosing these pathophysiological conditions, and thus enable timely medical interventions to achieve better outcomes. However, existing methods are not designed for continuous monitoring on individual patients: some necessitate costly equipment, such as magnetic resonance imaging; some rely on radioactive tracers, such as positron emission tomography^10^. Ultrasonography can image internal tissues and blood flow, but requires an operator and a separate lasing system for biomolecule sensing^11^. The recent advances in soft electronics have given rise to soft patches that can adhere to the human skin for continuous health monitoring^12–14^. These devices have demonstrated their capability in biomolecule sensing based on electrochemical reactions^15–22^ and optics^23,24^. However, existing soft patches can only sense biomolecules close to the skin surface. None of them has access to biomolecules in deep tissues, which have a stronger and faster correlation with the physiological and metabolic processes in the human body than those close to the skin surface^25^ (Supplementary Note 1, Supplementary Table 1).

Here we report a photoacoustic patch for continuous sensing of biomolecules in deep tissues. The device integrates an array of high-power VCSEL diodes and piezoelectric transducers, which are interconnected by serpentine metal electrodes and encapsulated in an elastomeric matrix. Pulsed laser emitted from the VCSEL array excites hemoglobin molecules to radiate acoustic waves. Those photoacoustic waves will be received by the transducer array and then processed to reconstruct a 3D map of the hemoglobin with a sub-millimeter resolution. Moreover, the photoacoustic signal amplitude has a linear relationship with the media temperature^26^, which provides a noninvasive way for core temperature measurement with a high spatial resolution and fast response. This work integrates laser sources and piezoelectric transducers into an electronic patch, which is unique in design, fabrication, and working principle among all existing wearable electronic patches (Supplementary Note 2).

## Results

### Design, fabrication, and working principle of the soft photoacoustic patch

**Fig. 1:**
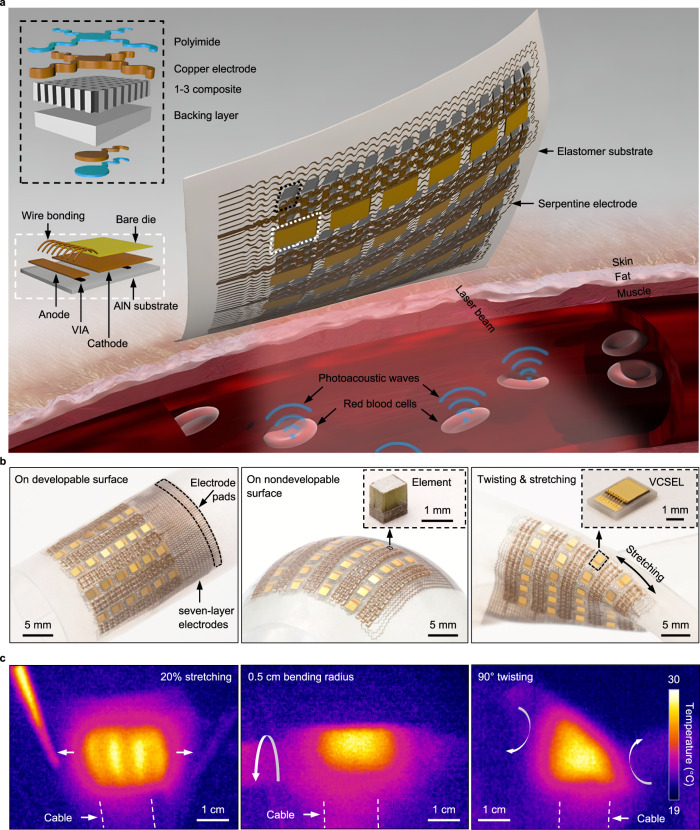
Design, fabrication, and working principle of the soft photoacoustic patch. **a** Schematics of the device structure and the working principle. The patch comprises an array of VCSELs and an array of piezoelectric transducers, interconnected by serpentine copper electrodes. All components are encapsulated in Ecoflex. Upon absorption of the optical energy, the hemoglobin molecules in red blood cells undergo thermoelastic expansion and radiate acoustic waves into the surrounding media. The photoacoustic waves will be collected by the transducer array and then relayed to a backend system for data processing. AlN: aluminum nitride. VIA: vertical interconnect access. VCSEL: vertical-cavity surface-emitting laser. **b** Optical photographs of the soft photoacoustic patch under different modes of deformation, including bending on a developable surface, wrapping on a nondevelopable surface, and stretching and twisting. Insets in the middle and right panels are optical micrographs of a single transducer element and a VCSEL diode, respectively. **c** Infrared camera images of the soft photoacoustic patch when the VCSELs (850 nm laser wavelength) are in operation under different modes of deformation, including stretching, bending, and twisting.

Figure 1a schematically illustrates the design and working principle of the soft photoacoustic patch. The patch includes a VCSEL array as the light source and a piezoelectric transducer array for photoacoustic wave detection. The laser beams are diffused in deep tissues. Hemoglobin molecules will undergo thermoelastic expansion after absorbing optical energy and collapse when the energy is absent. Therefore, when illuminated by the pulsed laser from the VCSEL array, hemoglobin will vibrate and emit acoustic waves. The piezoelectric transducers will receive the acoustic waves for generating the spatial distribution of the wave emitters. Therefore, photoacoustic imaging takes advantages of the unique absorption characteristics of biomolecules and highly penetrating acoustic waves to achieve high spatial resolution mapping of biomolecules in deep tissues.

In the patch, 24 VCSELs are evenly distributed in four equally spaced columns (Methods, Figs. S1–S3). The VCSELs in each column are connected in series. The distributed VCSEL layout can help generate uniform illumination in regions below the patch (Fig. S4). 240 piezoelectric transducers are arranged in between the VCSELs, in 15 columns with 16 transducers in each column. To address each transducer independently with a compact device profile, seven layers of serpentine interconnects are designed (Fig. S5). Four adjacent elements in the column are virtually connected in parallel to enhance the signal in the image reconstruction process (Fig. S6), forming 13 linear arrays in the row direction (Fig. S7). The overlap between adjacent arrays can increase the number of imaging planes, further improving the lateral resolution in the overlapping direction (Fig. S8). The VCSELs, transducers, and interconnects are all encapsulated in an elastomeric polymer, forming an “island-bridge” structure with an overall footprint of 2.0 cm × 1.6 cm and a thickness of 1.2 mm (Methods). In comparison with conventional photoacoustic imaging systems that need complicated components and strict operation environments (Fig. S9, Supplementary Note 3, Supplementary Table 2), the integrated wearable photoacoustic patch greatly reduces the physical constraint on the human body, potentially allowing imaging on moving subjects.

High-power VCSELs are used in this study to achieve a great detection depth and a large signal-to-noise ratio (SNR). A wavelength of 850 nm is used because it has deep-tissue penetration and is in the first optical window for probing human tissues^27,28^ (Supplementary Note 4). Hemoglobin also has the dominant optical absorption coefficient compared with other molecules, such as water and lipid, at this wavelength. Furthermore, VCSEL at 850 nm wavelength is most commonly available because, on the one hand, 850 nm is a common optical wavelength whose attenuation in fibers is relatively low^29^; on the other hand, silicon-based 850 nm photodetectors are low-cost and widely used^30^. The receiving transducer element is composed of a piezoelectric layer and a backing layer (Figs. S10, S11). The piezoelectric layer is made of 2 MHz lead zirconate titanate (PZT) micropillars embedded in epoxy. Compared with bulk PZT, the 1–3 composite suppresses the transverse vibration and enhances the axial vibration of the PZT micropillars, thereby increasing the electromechanical coupling coefficient and improving the energy conversion efficiency. The backing layer, made of cured silver epoxy, has a high electrical conductivity and a strong attenuation effect on acoustic waves to dampen excessive vibrations and thus improve the signal bandwidth and axial resolution of the transducers (Fig. S12).

The as-fabricated soft photoacoustic patch is mechanically and electrically robust. Figure 1b shows the photographs of the patch under different modes of deformation, including bending on a developable surface, wrapping on a nondevelopable surface, and twisting and stretching. The photoacoustic patch is rigid locally at each piezoelectric transducer element and laser diode but soft globally on the system level. No external pressure is required to conformally attach the photoacoustic patch to the skin (Fig. S13). Figure 1c presents the infrared camera images of the patch during operation (850 nm laser wavelength). Mechanical deformations do not affect the performance of the VCSELs (Fig. S14).

### Optical, thermal, and acoustic characterizations of the soft photoacoustic patch

**Fig. 2:**
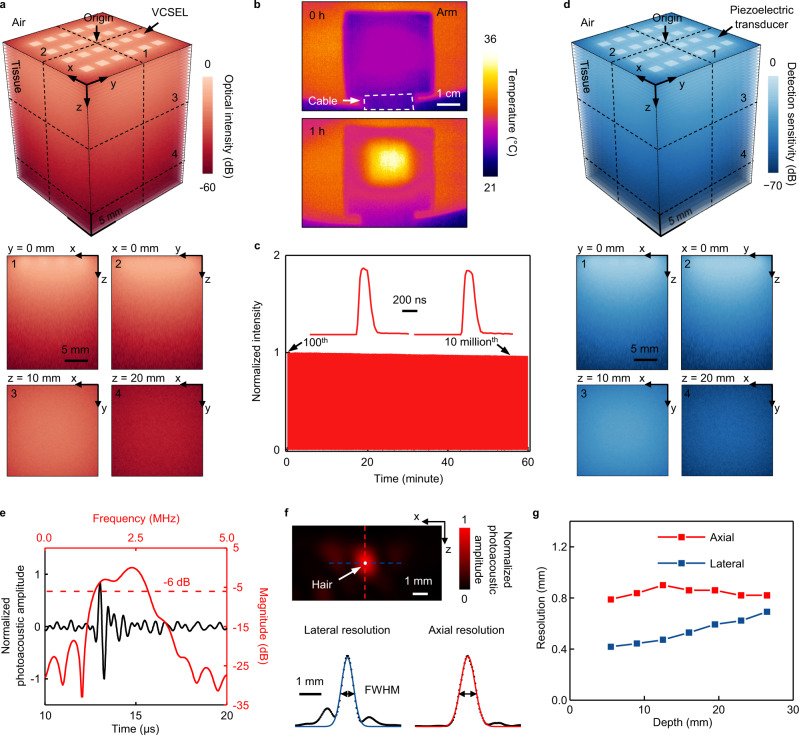
Characterizations of the soft photoacoustic patch. **a** Simulated optical intensity distribution in tissue. The 3D distribution map comprises 51 horizontal planes stacked together with display transparency of 60%. The four slices at the bottom panel highlight the optical intensity distribution at different cross-sections. VCSEL: vertical-cavity surface-emitting laser. **b** Thermal imaging of the photoacoustic patch on the arm immediately after turning on the lasers and after one hour of continuous operation. The maximum temperature is below 36 °C, which is comfortable for long-term wear. **c** Testing of the VCSELs’ output stability as they continuously work for 1 h with a pulse duration of 200 ns at a pulse repetition frequency of 3 kHz. The insets correspond to the 100th and the 10 millionth pulses. The decrease in normalized light intensity after an hour is <4%. **d** Simulated photoacoustic detection sensitivity distribution in tissue. The 3D distribution map comprises 51 horizontal planes stacked together with display transparency of 60%. The four slices at the bottom panel highlight the detection sensitivity distribution at different cross-sections. **e** Photoacoustic impulse response of the patch, in both time and frequency domains, characterized by detecting signal of a hair excited by the VCSELs. **f** A photoacoustic image of a hair at a depth of 2 cm in a gelatin phantom. The blue and red curves are the Gaussian fit to the lateral and axial photoacoustic amplitude profiles (black dots), respectively. The lateral and axial resolutions are determined by the FWHM in different directions. **g** The lateral and axial resolutions at different depths.

The optical energy distribution in the tissue should be as uniform as possible to minimize systematic artifacts introduced to the photoacoustic images. Optical attenuation needs to be minimal to ensure the greatest detection depth. Figure 2a shows the simulated optical intensity in a 2 cm × 2 cm × 2.5 cm human muscle tissue (Methods). Each VCSEL emits a laser beam perpendicular to the diode surface into the tissue with a divergence angle of 20° and a peak power of 40 W per VCSEL (Methods). The top surface in Fig. 2a corresponds to the interface between the patch and the tissue. The simulated optical intensity distribution in four planes cross-sectioning the illuminated volume is characterized (the bottom panels of Fig. 2a). The origin of coordinates is set at the central point of the photoacoustic patch. The optical intensities in the plane 1 (XZ plane at *y* = 0) and plane 2 (YZ plane at *x* = 0) show uniform distribution in the horizontal direction and small attenuation along the axial direction. In the XY plane, the distributions are highly uniform, with 20% and 2.3% of the incident intensity at the depths of 1 cm (plane 3) and 2 cm (plane 4), respectively. This indicates the ability of the laser beams to penetrate thick tissue layers. The optical intensity distributions of a stretched, bent, and twisted VCSEL array under normal mechanical deformations induced by the skin are tested. The 20% uniaxially stretched array shows an optical distribution very similar to that of an undeformed array (Fig. S15, Methods).

VCSELs in operation will generate a lot of heat. Excessive heat will not only raise safety concerns, but also degrade the VCSEL performance^31^ and change the sensitivity of piezoelectric transducers (Figs. S16, S17). Figure 2b shows the thermal images of the patch on a human arm immediately after turning on the lasers (top panel) and after continuous operation for an hour (bottom panel) at a repetition frequency of 3 kHz and a pulse duration of 200 ns (Fig. S18). A relatively long pulse, i.e., 200 ns, is used to enhance the signal-to-noise ratio, which is close to the pulse duration in other studies that utilize LEDs or laser diodes as the light sources^32–34^ (Fig. S19). After one-hour operation, the maximum temperature measured was ~36 °C, slightly higher than the skin surface temperature, but still comfortable for the subject. The photoacoustic patch generates as much heat as ultrasound-phased arrays^35^, both of which are within safety standards. Figure 2c presents the changes in incident intensity from the VCSELs during continuous operation. At 3 kHz pulse repetition frequency and 200 ns pulse duration, the intensity decreases by only <4% after one hour, showing the high stability of the VCSELs.

For quantitative photoacoustic studies, it is critical for the transducer array to have a uniform distribution of detection sensitivity to photoacoustic signals in the target region. Figure 2d shows the simulated photoacoustic sensitivity distribution of the patch in the 2 cm × 2 cm × 2.5 cm human breast tissue in consideration of nonuniform light distribution in Fig. 2a (Methods, Fig. S20). Due to the remarkable penetration of acoustic waves in human tissues, the detection sensitivity loss caused by the wave-sensing ability of piezoelectric transducers is < −10 dB. The high detection sensitivity ensures the high imaging depth of the photoacoustic patch. The tested transmitting and receiving properties of piezoelectric transducers demonstrated their high penetration depth and uniform sensitivity (Figs. S21–S23, Methods).

The impulse response is a critical characteristic of a sensing system (Methods, Figs. S24, S25), which is characterized by the time domain photoacoustic signal of a linear source excited by the VCSELs in this study. We measured the photoacoustic signals of a human hair (with a diameter of ~80 µm). The working frequency of the system is then characterized by applying Fourier Transform to the temporal photoacoustic signal (black curve) received by one transducer element (Fig. 2e). As the optical intensity of VCSELs is much lower than a conventional bulky laser, photoacoustic signals in the time domain are averaged to increase the SNR. On the other hand, the times of averaging will reduce the frame rate of imaging. To balance the SNR and frame rate in this study, the times of averaging are 3000 (Fig. S26), yielding a frame rate of 1 Hz and a SNR^36–38^ of 26.8 dB (signal of a hair at a depth of 2 cm in a gelatin phantom) at a lasing pulse repetition frequency of 3 kHz. The red curve shows the impulse response in the frequency domain, with a center frequency of 2.40 MHz and a bandwidth of 1.47 MHz (Figure 2e).

Imaging resolutions are characterized based on a linear source. Photoacoustic images are reconstructed based on signals generated by hairs embedded in gelatin phantoms at different depths^39–42^. Figure 2f displays a 2D photoacoustic image of a hair at a depth of 2 cm (Methods, Fig. S27). The amplitude profiles (black dots) of the photoacoustic image in the lateral and axial directions are fitted by the Gaussian function, illustrated by the blue and red curves, respectively (the bottom panel). The image resolution is determined by the full width at half maximum (FWHM) of the Gaussian curve fit to the profile^40–43^. The axial resolution is mainly determined by the signal frequency and remains almost constant at ~0.8 mm for different imaging depths (Fig. 2g). The lateral resolution will be reduced from ~0.4 mm to ~0.7 mm as the imaging depth increases because of the degraded focusing (Fig. 2g).

### Ex-vivo 3D hemoglobin mapping and core temperature measurement

**Fig. 3:**
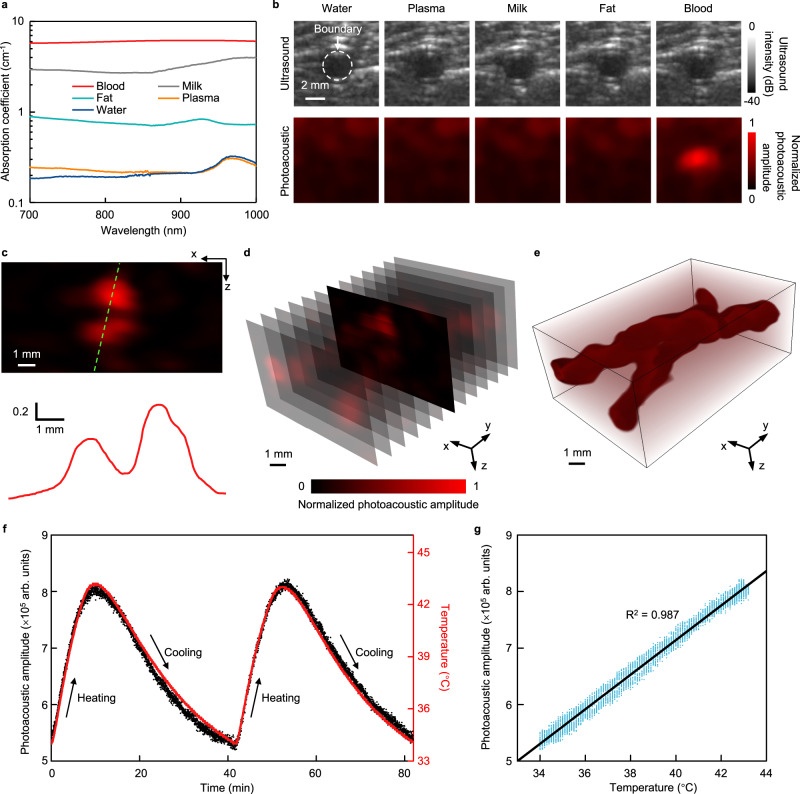
Ex-vivo 3D imaging of hemoglobin and monitoring of core temperature in deep tissues. **a** Optical absorption spectra of different body fluids. Absorption coefficients of water, plasma, milk, fat, and bovine whole blood are 0.197, 0.214, 2.716, 0.722, and 6.114 cm^−1^ at the wavelength of 850 nm, respectively. **b** Ultrasound B-mode and photoacoustic images of different cysts embedded in a porcine tissue at a depth of 2 cm. The ultrasound B-mode images, acquired by a commercial ultrasound probe, show no differences because of the low acoustic impedance contrast between various fluids. The soft photoacoustic patch differentiates the blood cyst based on the high optical absorption contrast. **c** A high-resolution photoacoustic image of a blood vessel phantom, which contains two silicone tubes filled with blood embedded underneath a 2 cm thick porcine tissue. The red curve at the bottom panel shows the profile along the green dashed line. **d** 13 slices of photoacoustic images of the blood vessel phantom with display transparency of 80%. **e** 3D imaging of hemoglobin at a depth of 2 cm in porcine tissue. **f** Comparison between the beamformed photoacoustic signal amplitude and temperature of flowing blood during two cycles of heating and cooling. **g** Linear fitting of the photoacoustic amplitude and temperature.

The wavelength of 850 nm is critical for a high penetration depth in human tissues^27,28^. Additionally, for photoacoustic mapping of hemoglobin amongst other biomolecules in the tissue, a laser wavelength where hemoglobin absorption is dominant needs to be selected. To characterize the sensing selectivity at this wavelength, we tested cyst phantoms with five different biofluid inclusions, including water, plasma, milk, fat, and bovine whole blood, in transparent colorless silicone tubes embedded underneath a 2 cm thick porcine tissue (Fig. S28). Figure 3a shows the measured optical absorption spectra of all types of biofluids (Methods), which shows that bovine whole blood has the dominant absorption coefficient at 850 nm. To further verify the selectivity, both ultrasound and photoacoustic images of the cyst phantoms are collected (Fig. 3b, Methods). Ultrasound-based B-mode images can only detect the acoustic impedance mismatch between different tissues, which is why the boundaries between the inclusions and matrixes are clear, while the types of biofluids are indistinguishable. The photoacoustic images are based on the contrast of optical absorptions, which differentiates the blood from other biofluids (Fig. S29).

16 rows of transducers form 13 linear arrays, each of which can produce a 2D photoacoustic image. Combining the 13 images, the patch can generate a 3D map of hemoglobin. The 3D mapping performance is tested on two crossed silicone tubes filled with bovine blood embedded underneath a 2 cm thick porcine tissue. Figure 3c shows a slice of the 3D map where the two silicone tubes overlap (top panel) and the corresponding photoacoustic signal amplitude profile along the green dashed line in the top panel (bottom panel). All 13 slices of the photoacoustic images are displayed in Fig. 3d, where the slice with overlapped tubes in Fig. 3c is highlighted. Figure 3e gives the integrated 3D mapping of hemoglobin at a depth of 2 cm (Figs. S30, S31). The patch can also distinguish two overlapping vessels (Fig. S32). When the stretching strain is within 15%, the patch performance is minimally affected (Fig. S33).

Core temperature is critical for governing the essential functions of the body and should be maintained near 37 °C^44^. It typically fluctuates within 1 °C according to circadian rhythm^45^, but can reach ~40 °C amid strenuous workload or ~35.6 °C in cold environments. A significant deviation of the core temperature indicates failing thermoregulation^44^ with dire consequences^46–49^, sometimes life-threatening. Most soft patches can only measure the temperature on the skin surface, which can be easily affected by the external environment and thus has a weak correlation to the core temperature. Noninvasive sensing of core temperature is mainly based on Zero-Heat-Flux^50,51^ or Dual-Heat-Flux^52,53^ thermal models, which have long response time (~3 min)^51^ and limited detection depths (~1 cm)^51^ (Supplementary Note 5, Supplementary Table 3).

Photoacoustic signals are generated when the biomolecules convert the pulsed optical energy to mechanical energy in the form of photoacoustic waves^54^. In the range of 10–55 °C, there is a linear relationship between the amplitude of photoacoustic waves and the temperature^26^ (Supplementary Note 6), allowing the measurement of temperature by the photoacoustic approach. As an initial test, we used the soft photoacoustic patch to measure the temperature in a phantom and checked its performance with thermocouples (Fig. S34). The phantom is composed of warm bovine blood injected in transparent silicone tubes underneath 2 cm thick room-temperature porcine tissues. Thermocouples were placed in the tubes, where the photoacoustic measurements were also taken. We demonstrate the high accuracy (Fig. S35, Supplementary Note 7), spatial temperature mapping, and fast response (Fig. S36) of the photoacoustic patch in core temperature measurement by detecting static blood, as validated by the thermocouple.

To monitor flowing blood, we tested the core temperature of an ex-vivo porcine tissue (Fig. S37). A pump drove the blood to flow in a transparent silicone tube with an inner diameter of 3 mm. The flow rate was set to be ~9 mL s^−1^, resulting in a blood flowing speed of ~127 cm s^−1^, faster than the blood flow velocity of most blood vessels in the human body^55^. The two ends of the tube were immersed in a beaker containing bovine blood, which was placed on a hot plate to heat the blood to different temperatures during flowing. The blood could also naturally cool down while the hot plate was turned off. A portion of the tube was embedded underneath a porcine tissue at a depth of ~2 cm, which was measured by the photoacoustic patch. A thermocouple was inserted into the tube to measure the blood temperature simultaneously and record the data continuously. Figure 3f shows the beamformed photoacoustic amplitude (black dots) of flowing blood measured by the photoacoustic patch and the temperature (red line) measured by the thermocouple, which agree well with each other during the entire dynamic process. Two cycles of heating and cooling were tested, lasting ~82 min. Figure 3g presents the scatter plot of the photoacoustic amplitude as a function of the thermometer temperature. The fitting of the measured data (*R*^2^ = 0.987) demonstrates the linear relationship between the photoacoustic amplitude and the temperature in flowing blood.

### In-vivo 3D imaging of blood vessels and venous occlusion test

**Fig. 4:**
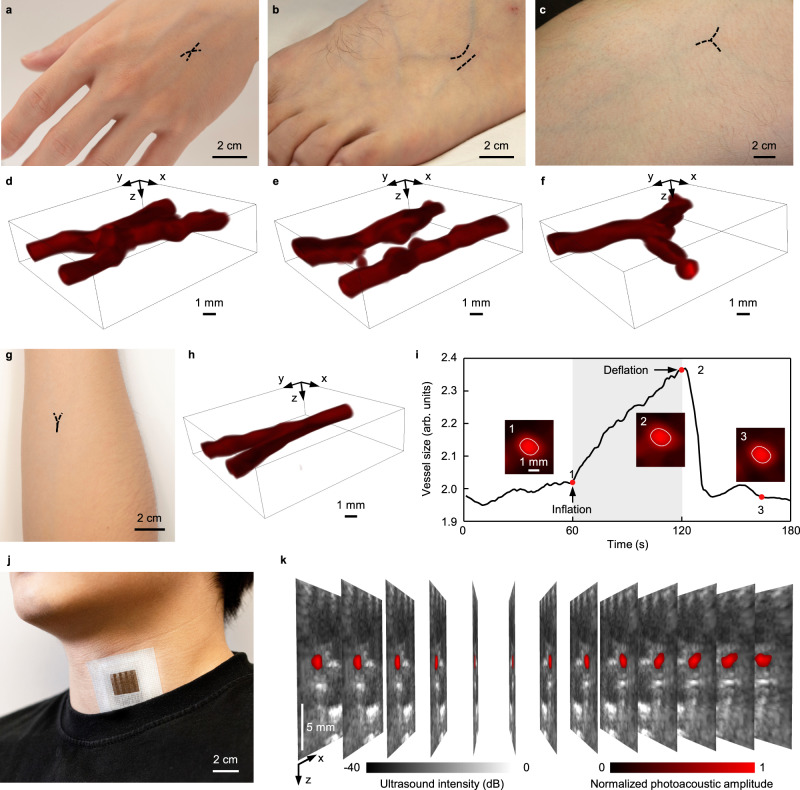
In-vivo imaging of blood vessels and venous occlusion test. **a–h** Photos of the hand, foot, thigh and forearm where the target veins are highlighted. The reconstructed 3D photoacoustic images are all shown accordingly. **i** Cross sectional size changes of the vein before cuff inflation, during cuff inflation with a pressure of 70 mmHg, and cuff deflation. The pixels inside the white boundary with a value >0.5 are counted to calculate the vein size. **j** A photograph of the patch attached to the human neck at a location above the internal jugular vein. **k** 13 slices of dual-mode images acquired by the photoacoustic patch, i.e., photoacoustic images of the internal jugular vein superimposed on ultrasound B-mode images. The pixel values of normalized photoacoustic images < 0.5 are not shown.

To test the feasibility of in-vivo monitoring, we used the photoacoustic patch to image veins in the hand, foot, thigh, and forearm, monitor the venous response to the occlusion test, and image the internal jugular vein (IJV) (Fig. S38, Methods). Figure 4a-h presents the photos of a volunteer’s hand, foot, thigh, and forearm, where the target veins are labeled. The photoacoustic patch acquired 13 cross sections of the veins, which were then converted to 3D images, respectively, as shown at the sides of corresponding photos. The 3D images clearly display the vein structures of different body locations. In comparison to Doppler ultrasound imaging^35^, photoacoustic imaging has high sensitivity and contrast in detecting blood vessels, especially for small blood vessels with slow blood flow^56^ (Fig. S39). Note that the flexible photoacoustic patch may also be affected by motion artifacts (Fig. S40), a common problem in existing wearable electronic devices^57–59^. The volunteer kept the arm static during the measurements to minimize any motion artifacts.

Venous occlusion plethysmography is a noninvasive tool to assess the blood flow and vascular resistance of limbs^60,61^. In the measurements, venous return from the forearm was briefly interrupted by inflating a cuff, wrapped around the upper arm, to above venous pressure but lower than the diastolic pressure (Methods). As a result, the venous dimension will increase as the arterial blood inflow. We attached the photoacoustic patch on the forearm, above the veins, and continuously monitored the dynamic vascular response to a venous occlusion (Supplementary Movie 1). Figure 4i shows the change of the vein size during a 3 min continuous recording. Insets show the photoacoustic images of the vein at three different moments. The image pixels with a normalized value >0.5 were counted into the vein area^62^, as labeled by the white boundaries. No pressure was applied in the first 1 min, thus no significant area change was observed. At 60 seconds, the cuff was quickly inflated to 70 mmHg, resulting in an increasing vein area with the time. Inset image 2 presents an obvious expansion of the vein compared to inset 1. The cuff was rapidly released after 1-min inflation, accompanied by a dramatic drop in the vein area. Those results are similar to others acquired by bulky photoacoustic systems^63^. The venous occlusion test demonstrated the fast response of the photoacoustic patch for in-vivo imaging.

We used the photoacoustic patch to 3D image the IJV (>1.1 cm in depth) in the neck (Fig. 4j and Fig. S41). Figure 4k shows 13 slices of photoacoustic images of the IJV superimposed on the corresponding ultrasound B-mode images (Fig. S42), which are all acquired by the photoacoustic patch. The central frequency of the ultrasound transducers is close to 2 MHz, which results in the low contrast of the IJV in the ultrasound B-mode image. On the contrary, the IJV shows high contrast to other surrounding tissues in the photoacoustic image (Fig. S43), benefiting from the strong optical contrast between the hemoglobin and other molecules. The irregular skin curvature has a minimal influence on the imaging resolutions of the photoacoustic patch, due to the relatively low acoustic working frequency (Figs. S44, S45 and Supplementary Note 8). The carotid artery is invisible in the photoacoustic images because its strong pulsation will induce unstable phases to the photoacoustic signals and therefore damage their coherent averaging^64,65^ (Fig. S46).

## Discussion

The soft photoacoustic patch demonstrated in this study allows for continuous, noninvasive mapping of hemoglobin and core temperature with high spatial resolution in real time. This work reports using soft electronic devices for 3D imaging of biomolecules in deep tissues (>2 cm in ex-vivo tests and >1.1 cm in in-vivo tests). The high-resolution imaging of hemoglobin will enable the monitoring of hemodynamics and vascular proliferation in tissues to manage a variety of conditions and diseases. Monitoring the dimension of blood vessels can be valuable for evaluating vessel functions and diagnosing vascular diseases. For instance, measuring the dynamic change of the vein diameter during an occlusion can help examine venous compliance, which is a strong indicator of cardiac function^60^. The photoacoustic effect-based temperature measurements, with the advantages of deep penetration, high accuracy, and fast response, introduce a strategy for monitoring the core temperature, e.g., during exercise, anesthesia, and surgical hypothermia, in fundamental biomedical research and clinical practice^66,67^.

Although the photoacoustic patch discussed here only detects hemoglobin, this platform technology can potentially be extended to monitor many other endogenous biomolecules, such as melanin^68,69^, glucose^70–72^, lipid^73,74^, cytochrome^75^, nucleic acid^76^, and proteins^77,78^ (Supplementary Note 9). Furthermore, exogenous contrast agents, like single-walled carbon nanotubes^79^, gold nanoparticles^80^, and methylene blue^81^, can further enhance the signal intensity, increase the detection depth, and improve the detection specificity^82^. The laser wavelength is the key to selectively monitoring various biomolecules. Integrating multiple laser diodes with different wavelengths on the photoacoustic patch can expand the portfolio of detectable biomolecules, with more accurate targeting of biomolecules by detecting a set of absorption characteristics at different wavelengths. It is possible to integrate two or more wavelengths VCSELs in the patch as VCSELs with a wide wavelength range have been developed, from blue light^83^ (447 nm) to infrared light^84^ (1550 nm), which overlaps with the typical wavelength range in photoacoustic imaging applications.

The current detection depth is still limited by the optical intensity of the VCSELs. It is challenging for the photoacoustic patch to image the cardiac region because the optical power of laser diodes used in this work is not as high as expensive bulky high-power lasers. The depth of the cardiac region ranges from several centimeters to >17 centimeters^85^. The average shortest distance between the heart and the skin is about 3.1 cm when detecting from the apical view^86^. This large imaging depth is currently not achievable by the wearable photoacoustic patch. In-vivo photoacoustic imaging of the human heart represents a grand challenge in the field, even using conventional bulky photoacoustic systems with expensive high-power lasers. In this work, we achieved an ex-vivo imaging depth of ~2 cm in porcine tissues and an in-vivo imaging depth of ~1 cm. Higher power VCSELs will be needed to further increase the detection depth to the regions of visceral organs. Additionally, developing higher power VCSELs, by either fabricating larger VCSELs with more light emitting elements (Figs. S3, S47) or constructing driving circuits with higher output current, will be essential for increasing the SNR of photoacoustic signals and thus reduce the times of averaging for imaging dynamic arteries.

Photoacoustic imaging without calibration can only monitor relative temperature changes. A calibration process that establishes the relationship between photoacoustic amplitude and absolute temperature can enable photoacoustic imaging to monitor absolute temperature. In this work, we demonstrated monitoring absolute core temperature in ex-vivo porcine tissues. While monitoring the photoacoustic amplitudes of blood, we recorded the absolute temperature simultaneously by a thermocouple. Therefore, the photoacoustic amplitude could be calibrated and transferred to absolute temperature. For in-vivo applications, photoacoustic imaging can monitor relative temperature changing without calibration. Invasive temperature catheters can be used for calibration. It is worth mentioning that for long-term monitoring, the calibration is only required once.

Before applying the photoacoustic patch to monitor blood temperature in the human body, additional challenges need to be solved. First, a gold standard technique for core temperature measurement is required to calibrate the photoacoustic amplitude. Some noninvasive core temperature sensors based on thermal flux models suffer from slow response and lack of spatial resolution, which are not suitable for calibration in this case. Invasive catheters can be directly inserted into the blood vessel to monitor temperature, but it is too invasive. Second, some other factors may also affect the photoacoustic signals, such as the amount of blood perfusion. More advanced methods should be developed to eliminate the influence of these factors, such as the thermal memory based photoacoustic technique^87^.

We adopted data averaging and bandpass filter to improve the signal-to-noise ratio. A high pulse repetition rate, i.e., 3 kHz, is used to ensure a high imaging frame rate (Supplementary Note 10). Although data averaging is a common and convenient way, thousands of times of averaging makes it time-consuming, causing more laser exposure. Besides, there are other methods for enhancing the SNR while requiring less time consumption (Supplementary Note 11), such as coded excitation^88–91^, empirical mode decomposition^92,93^, wavelet thresholding^94,95^, Wiener deconvolution^96^, and adaptive filtering^97^. These methods and the data averaging can be adopted together to achieve better SNR^92^. In the current design, bulky ultrasound probes and sophisticated laser machines are eliminated, which have significantly improved the device portability and ease of use, but the photoacoustic patch is still wired to a backend system for signal acquisition and processing. Future efforts can focus on miniaturizing the control electronics to realize a fully integrated wearable system and, therefore, enable measurements on-the-go (Fig. S48).

## Methods

### Human experiment protocols

All human tests were performed under University of California San Diego Institutional Review Board (IRB) approval (number 800975). One 25-year-old male volunteered to be tested with informed consent obtained without compensation. For the venous occlusion experiment, the volunteer sat on a chair with a pressure cuff worn on the upper arm. The vertical distance between the neck and the forearm was about 30 cm. Then we attached the photoacoustic patch on the forearm above the veins using a medical tape. After that, the venous occlusion was performed: (1) No pressure was applied to the cuff in the first 1 minute; (2) inflate the cuff to 70 mmHg immediately and maintain for 60 s; (3) deflate the cuff to zero to let the veins recover to the normal status. In the detection of the internal jugular vein, the volunteer sat on a chair with the photoacoustic patch attached to the neck with a medical tape. For the imaging of veins in the hand, foot, thigh, forearm and venous occlusion test, a 1 cm-thick gelatin phantom was placed between the patch and skin to compensate non-uniform light distribution (Fig. S38).

### Fabrication of laser diode chips

The fabrication process of the VCSEL diode chip is schematically illustrated in Fig. S2. The anode and cathode of the VCSEL die (850 nm, Ace Photonics) are on the top and bottom surfaces (Fig. S3), respectively. To facilitate the fabrication of the photoacoustic patch, the anode and cathode are routed to the same surface by creating vertical interconnect accesses (VIAs) and wire bonding. Two vertical openings were created by laser ablation in a 1.7 mm × 2.4 mm × 0.25 mm aluminum nitride (AlN) substrate and filled with silver epoxy (E-Solder 3022). The silver epoxy VIAs were cured in an oven at 80 °C for 2 h. The AlN substrate was cleaned with acetone and isopropyl alcohol to remove organic contaminants, followed by rinsing with DI water and drying with nitrogen gas. Moisture induced in the cleaning process was removed by baking the samples in a vacuum oven at 100 °C for 10 min. A lift-off process allowed patterning metal electrodes on AIN. The process involved photolithography (photoresist AZ 1529: spin-casting at 4000 r.p.m. for 60 s, baking on a hotplate at 95 °C for 120 s, UV irradiance at 350 mJ cm^−2^, and developing for ~40 s with developer AZ 300 MIF) and then sputtering (Ti: 200 W, 3.0 mTorr, 5 sccm Ar, 5 min, ~50 nm; Au: 200 W, 3.0 mTorr, 5 sccm Ar, 15 min, ~400 nm). The sample surface was activated (reactive ion etching: 50 W, 50.0 mTorr, 35–40 °C, 50.0 sccm O_2_, 30 s) before sputtering. The samples were soaked in acetone for 30 min to thoroughly remove all photoresists and lift off the metals on the top of the photoresists. Moisture induced in the lift-off process was removed by baking the samples in a vacuum oven at 100 °C for 10 min. The VCSEL die was then pasted on the ground electrode pad on AlN with silver epoxy, which was cured in an oven at 80 °C for 2 h. The anodes of the VCSEL die and AlN substrate were connected with wire bonding.

### Fabrication of the photoacoustic patch

The fabrication process can be generalized into three steps: (1) patterning of the stretchable multilayered electrodes, (2) preparation of the VCSEL diode chips and ultrasonic transducer array, and (3) soft packaging. Cu foils with 20 μm thickness were used as the multilayered conductive interconnects. To adhere the interconnects on the soft elastomeric substrate tightly, a PI thin film [poly(pyromellitic dianhydride-co-4,40-oxydianiline) amic acid solution, PI2545 precursor, HD MicroSystems] was spin-coated on the Cu, at the speed of 4000 r.p.m, with an acceleration of 5000 r.p.m per second, for 60 s. The PI was cured by soft baking at 100 °C for 3 min and hard baking at 300 °C for 1 h under a nitrogen atmosphere. The PI-based Cu foil was activated by ultraviolet light (PSD series Digital UV Ozone System, Novascan) for 2 min and then laminated on a temporary PDMS substrate (base to hardener ratio is 20:1, Sylgard 184 silicone elastomer). The ultraviolet light activation strengthens the bonding between the PI and the PDMS substrate. A nanosecond laser (Laser Mark’s, central wavelength, 1059–1065 nm; power, 0.228 mJ; frequency, 35 kHz; speed, 300 mm s^−1^; and pulse width, 500 ns) was used to ablate the Cu/PI into the “island-bridge” serpentine layout. The electrode patterns were designed by AutoCAD (Autodesk, USA). The patterned Cu/PI thin film was transfer-printed to an Ecoflex substrate (15 μm thick; Ecoflex-0030, Smooth-On) on a glass slide using a water-soluble tape (3 M) after activation by ultraviolet light for 3 min. To tightly stack the second layer of the electrode on top of the first layer, a dielectric layer (15 μm) of Ecoflex was spin-coated on the first layer. Using the same method, six layers of top stimulation electrodes were built up and aligned under the microscope. The VIAs were developed by laser ablation to route all electrodes in multiple layers to the same plane. The VCSEL array was bonded with the six-layer electrode using silver epoxy (Esolder 3022, EIS, USA). Anisotropic conductive films (Elform) were hot pressed to the front pads of the electrodes to connect the patch to the external power supply and the data acquisition system. The bottom common ground electrode was fabricated in a similar way to the top electrodes.

The structure of the ultrasonic transducer consists of a piezoelectric material and a backing layer. 1-3 PZT-5A composites (Del Piezo, USA) were selected due to their high electromechanical coupling coefficients. The condensed backing layer was made of silver epoxy (Esolder 3022, EIS, USA) for absorbing the extra ultrasonic waves. The silver epoxy composite was mixed with the hardener in a 12.5:1 ratio over 10 min and mounted on a 0.3 mm thick mold, which was then cured at 80 °C for 2 h. The same silver epoxy was used to integrate the backing layer with the 1-3 composite material, and the entire piece was diced into multiple small elements (0.8 mm length × 0.6 mm width × 1 mm thickness).

A scaffold with 240 openings was customized to fix the piezoelectric transducers. Connections to the top and bottom electrodes were achieved with the conductive adhesive at 80 °C for 2 h. The device was encapsulated by filling the device with the uncured Ecoflex precursor, followed by curing at 80 °C for 20 min. After that, the glass substrates carrying the top and bottom electrodes were peeled off. VCSEL chips and piezoelectric transducers are connected to external driving and signal acquisition systems with wires. The connection of VCSEL chips can be integrated with that of the piezoelectric transducers, which does not increase the complexity of the overall wearable patch compared to ultrasound sensors^98^.

### Simulation of optical distribution

The simulation of the optical intensity distribution in a 3D space was performed by the Monte Carlo method using an open-source MATLAB toolbox—MCmatlab^99^. A 4 cm × 4 cm × 4 cm homogeneous region was set as the human breast tissue, with the absorption coefficient *μ*_*a*_, scattering coefficient *μ*_*s*_, Henyey – Greenstein scattering anisotropy factor *g*, and refractive index *n* set as 0.1 cm^−1^, 85 cm^−1^ ref. 100, 0.9 ref. 99, and 1.3, respectively. The region above the top surface was considered as air, with *μ*_*a*_, *μ*_*s*_, *g*, and *n* set as 1 × 10^−8 ^cm^−1^, 1 × 10^−8 ^cm^−1^, 1, and 1, respectively. The laser diode array was placed at the center of the top surface. The width of each laser source was 1.5 mm. Each laser diode emitted a laser beam into the tissue perpendicular to the surface with a divergence angle of 20°. All the boundaries were set to be cuboid. The wavelength was 850 nm.

### Simulation of photoacoustic detection sensitivity

The simulation of photoacoustic detection sensitivity was performed in a 4 cm × 4 cm × 4 cm homogeneous region using an open-source MATLAB toolbox ― k-Wave^101^. The transducer array was placed at the center of the top surface. Assuming the background tissue as the human breast, the sound speed and tissue density were set as 1510 m s^−1^ and 1020 kg m^−3^, respectively. The frequency dependent acoustic absorption coefficient was considered as 0.75 dB (MHz^*y*^ cm)^−1^, where *y* equals to 1.5 ref. 102. The simulation region was divided into voxel elements with a pitch of 0.05 mm in each direction. In each voxel, one point source emitted a pulsed photoacoustic signal with the amplitude decided by the light distribution. All transducers received the pulse signal, followed by Delay-And-Sum beamforming. The amplitude of the beamformed signal was the detection sensitivity of this voxel.

### Characterization of VCSEL array

The laser power of a single VCSEL chip is about 40 W measured by a power meter (Newport Corporation, 835 Optical Power Meter, 818-SL detector, 883-SL attenuator), which has a sensing aperture of 11.3 mm to cover the entire light beam of one VCSEL. Considering the entire patch with a footprint of 2 cm × 1.6 cm, the average power is about 1.8 × 10^3 ^W m^−2^, which is lower than the safety limit^103^ of 3.99 × 10^3 ^W m^−2^. Smaller pulse repetition frequency can be selected to further reduce the power if needed by specific use cases. To detect the light fields of optical beams in different cases, including a single VCSEL, and an undeformed, stretched, bent, or twisted VCSEL array, we scanned a photodetector point by point in free space to measure the optical intensity in a 2D plane. An optical attenuator (Thorlabs, NE60A-B) was fixed on the photodetector (Thorlabs, PDA10A2) to make sure the optical intensity does not exceed the measurement range of the photodetector. The scanning plane was 3 cm away from the VCSEL and VCSEL array. We measured five optical fields with a size of 2 cm × 2 cm and a step size of 1 mm (Fig. S15).

### Characterization of piezoelectric transducers

The transmitting sound field of a transducer element was measured using a hydrophone (ONDA, Model no. HNP-0400) in a water tank (Fig. S21). The piezoelectric transducer was excited by a pulse voltage of 100 V. A hydrophone scanning system (ONDA, AIMS III) moved the hydrophone in the 3D space. The peak-to-peak value of the signal measured by the hydrophone was extracted. To test the receiving sensitivity of the piezoelectric transducers, the performances of the photoacoustic patch and the commercial probe P4-2v from Verasonics were compared. P4-2v was chosen because it has a central frequency of about 2.7 MHz, close to the transducers (~2.4 MHz) in the photoacoustic patch. A 100 V pulse was applied to a customized single transducer (0.5 mm × 0.5 mm) to emit ultrasound waves, which was measured by both the commercial probe and the photoacoustic patch (Fig. S22). Signals measured by four elements of the photoacoustic patch were summed, which was the same as the case in the practical applications. To map the receiving sensitivity in 3D space, the single transducer ultrasound source was moved by the scanning system (ONDA, AIMS III) to emit ultrasound waves, which was measured by the photoacoustic patch and beamformed in the Verasonics system. The peak-to-peak value of the beamformed signal was extracted (Fig. S23).

### System setup and data collection

Verasonics Vantage 256 worked as the host to control the timing sequence of the whole system and signal acquisition. It has 256 individual signal acquisition channels with built-in low-noise amplifier, programmable gain amplifier and filters. That means each element receives the photoacoustic signal independently. All of the elements can receive the data simultaneously. Signals of four elements will be summed digitally in the MATLAB program to form one element in each virtual linear array. A program was written by MATLAB and run on the Verasonics system, controlling the laser radiation and photoacoustic signal acquisition.

To synchronize the laser emission and signal acquisition, Verasonics exported a 3.3 V LVTTL-compatible trigger signal to the signal generator (Rigol, DG822), which was a 1 μs active low output. The signal generator would be triggered to output a 5 V pulse signal with a duration of 200 ns. The laser driver (PicoLAS, LDP-V 240-100 V3.3) received the output from the signal generator, and immediately provided a 50 A current to drive the laser diodes with a pulse duration of 200 ns. The peak power of each VCSEL was 40 W driven by a 50 A pulse current. After laser illumination, the Verasonics system started the signal acquisition process. The recorded photoacoustic signal was digitized at a sampling frequency of 62.5 MHz and filtered by a bandpass filter with a center frequency of 2.2 MHz and −6 dB bandwidth of 1.2 MHz. To enhance the SNR, photoacoustic signals were averaged 3000 times to reduce the incoherent noise. Verasonics controlled the VCSELs to emit laser beams and transducers to receive signals at a pulse repetition frequency of 3 kHz, resulting in a detection frame rate of 1 Hz. A C-language program was written and called in MATLAB by the host program to reconstruct the 2D images. Reconstructing one 2D image takes about 50 ms, which means ~0.65 s is required to reconstruct all of the 13 slices of 2D images. These slices of 2D images can be shown during the measurement in real-time, which reveal information in 3D space. Converting the 2D images into 3D image was manually processed offline in a software (Amira) after saving all of the 2D images. The processing time took <20 s. The conversion from 2D images to 3D image may be processed automatically in MATLAB in the future to save time. The time-domain signals were also saved for offline processing to reconstruct the 3D images.

The human skin and driving electrodes that connect VCSEL diodes are isolated by a 1 mm-thick Eco-flex 00–30 layer. As reported^104^, the leakage current for such a silicon polymer layer with the same thickness is as low as 10^−11^ A at an applied electric field of 5 V μm^−1^. Since the applied electric field in this study is less than 1 V μm^−1^, the leakage current should be smaller than 10^−11^ A for the photoacoustic patch, which is very safe. In the ex-vivo temperature measurements (Fig. 3f, g), to avoid direct illumination on the thermocouples (Omega Engineering Inc., Model no. SC-TT-K-30-36) by the laser beam and getting photoacoustic signals from them, the thermocouples were placed at the peripheral region of the photoacoustic patch. Supplementary Tables 4 and 5 list the detailed information of experimental equipment and material used in this study, respectively.

### Image reconstruction algorithms

The Coherence-Factor-weighted-Delay-And-Sum (CFDAS) algorithm was applied to reconstruct photoacoustic images. For the unmodified DAS beamforming algorithm, assuming the photoacoustic signals are measured by a transducer array with *M* elements, the received signal of each channel is expressed as *p*_*m*_(*t*). To reconstruct the image *I*(*x*, *z*) at pixel (*x*, *z*), the wave propagation time from the pixel to the *m*-th element is calculated as *∆t*_*m*_. Therefore, the image *I*(*x*, *z*) could be computed through the summation of ${\sum }_{m=1}^{M}{p}_{m}(\triangle {t}_{m})$. The CFDAS introduces an adaptive coherence factor as an additional weight to ${\sum }_{m=1}^{M}{p}_{m}(\triangle {t}_{m})$, which is ${{\mbox{CF}}}=\frac{{\left|{\sum }_{m=1}^{M}{p}_{m}(\triangle {t}_{m})\right|}^{2}}{M\bullet {\sum }_{m=1}^{M}{\left|{p}_{m}(\triangle {t}_{m})\right|}^{2}}$ ^105^. CFDAS has been demonstrated to improve the image quality^106^ (Fig. S27). The reconstructed 2D images were combined in Amira to form 3D images. The gaps between the 2D image slices were smoothed by Amira automatically.

### Test of optical absorption spectra

The NIR-UV-Vis measurements were carried out through a PerkinElmer lambda 1050 UV/Vis/NIR Spectrometer. Water absorbance spectrum was measured under 150 mm InGaAs Int. Sphere Absorbance module and the rest were carried out through 3D WB Det. Absorbance Module. Before each measurement, a 100% transmittance (0 absorbance) baseline was auto-zeroed. The water spectrum was denoised through white certified reflectance standard from Labsphere Company while the rest background was calibrated with pure water. The detection cuvette had a transmittance length of 5 mm. The injected beam (Slit width of 2.00 nm) was sourced from the combination of D2 Lamp and Tungsten Lamp with a lamp change at 860.8 nm. The spectra were collected in the wavelength range from 1000 nm to 700 nm with a data interval of 1 nm.

### Acquisition of ultrasound B-mode images

The ultrasound B-mode images of cyst phantoms were acquired by the Verasonics Vantage 256 with a L11-5v linear array. The center frequency of the probe was 7.8 MHz. The compounding imaging strategy was applied to reconstruct the images, which transmitted plane waves in 21 directions, received the echoes, and combined them all to form a single image.

### Reporting summary

Further information on research design is available in the Nature Portfolio Reporting Summary linked to this article.

## Supplementary information

Supplementary Information

Description of Additional Supplementary Files

Supplementary Movie 1

Reporting Summary

## Data Availability

All data supporting the findings of this study are available within the paper and its Supplementary Information. The data generated in this study are available from a public data repository at https://figshare.com/articles/dataset/PA_patch/21440925.
