# Supplementary material for: A photoacoustic patch for three-dimensional imaging of hemoglobin and core temperature
Source: Nat Commun. 2022 Dec 15;13:7757. doi: 10.1038/s41467-022-35455-3 (PMC9755152; doi:10.1038/s41467-022-35455-3)
Supplement: Supplementary file 1 — Supplementary Information [file 41467_2022_35455_MOESM1_ESM.pdf]

Supplementary Information for

A photoacoustic patch for three-dimensional imaging of hemoglobin and core temperature

Xiaoxiang Gao[1,†], Xiangjun Chen[2,†], Hongjie Hu[1,†], Xinyu Wang[1], Wentong Yue[1], Jing Mu[2], Zhiyuan Lou[1], Ruiqi Zhang[1], Keren Shi[2], Xue Chen[2], Muyang Lin[1], Baiyan Qi[2], Sai Zhou[2], Chengchangfeng Lu[3], Yue Gu[2], Xinyi Yang[2], Hong Ding[1], Yangzhi Zhu[1], Hao Huang[1], Yuxiang Ma[1], Mohan Li[1], Aditya Mishra[2], Joseph Wang[1,2], Sheng Xu[1,2,3,4,5,*]

1. Department of Nanoengineering, University of California San Diego, La Jolla, CA 92093, USA

2. Materials Science and Engineering Program, University of California San Diego, La Jolla, CA 92093, USA.

3. Department of Electrical and Computer Engineering, University of California San Diego, La Jolla, CA, USA.

4. Department of Radiology, School of Medicine, University of California San Diego, La Jolla, CA 92093, USA.

5. Department of Bioengineering, University of California San Diego, La Jolla 92093, CA, USA.

†These authors contributed equally to this work.

*Corresponding author. E-mail: shengxu@ucsd.edu

Supplementary Note 1 | Comparison between different biomolecular imaging methods

Conventionally, there are several non-invasive methods for detecting biomolecules with high penetration depth (>10 cm) such as magnetic resonance imaging (MRI)[1,2] and positron emission tomography (PET)[3,4]. MRI leverages the different magnetic properties of various biomolecules to generate images. More specifically, those biomolecules possess multiple relaxation times after receiving radiofrequency pulses. PET is based on the detection of two annihilation photons, which are generated by collisions of the injected radioactive tracers and electrons within the tissue. The tracers will only couple with the target molecules so that high contrast will be achieved. However, the existence of radioactive tracers prevents this technique from long-term use. For these two methods, the associated equipment is too cumbersome and expensive, thus impossible for wearable long-term health monitoring.

Several optical methods are used for biomolecular imaging. The principle of fluorescence imaging is that materials will emit fluorescent light at a specific wavelength after absorbing high energy photons. Different molecules possessing various molecular energy structures result in various fluorescence. Although the spatial resolution is high (~4 μm), this technique is highly limited by its shallow penetration depth (~3 mm)[5]. Optical coherence tomography (OCT) typically uses near-infrared light for imaging. The backscattered light is measured with an interferometric setup to reconstruct the depth profile of the tissue[6]. But the penetration depth is still limited (~2 mm)[7].

Photoacoustic imaging involves shining a laser beam onto tissues. After that, the light energy is absorbed by the biomolecules and converted to mechanical vibration energy, i.e., photoacoustic waves. Photoacoustic imaging entails several advantages[8] compared to the aforementioned optical

imaging: (1) by illuminating various molecules at different wavelengths, photoacoustic tomography exhibits high contrasts regarding chemical compositions; (2) the spatial information of biomolecules is encoded in the ultrasound waves, which has relatively weak attenuation in biological tissues. Therefore, photoacoustic imaging can achieve high spatial resolution (tens of micrometers) mapping of biomolecules in deep tissues (several centimeters in depth)[9,10]. As of now, existing photoacoustic imaging devices are bulky and cumbersome, not suitable for wearable long-term continuous use.

The emergence of wearable devices such as soft electrochemical electronics[11] and soft optoelectronics[12] makes continuous monitoring possible. Nevertheless, these devices are still limited by the shallow detection depth beneath the skin. Additionally, these devices can only provide measurements at specific locations, lacking spatial resolutions.

The soft photoacoustic patch in this work inherits the merits of photoacoustic imaging, which is biomolecular selectivity and high imaging resolution in deep tissues. Also, the flexible and stretchable configuration allows the device to be conformally attached to the skin, which can potentially enable convenient and continuous measurements on the go. By innovative designs in device layout and advanced data processing methods of the soft photoacoustic patch, we achieved a detection depth >2 cm with high spatial resolution in biological tissues.

Supplementary Note 2 | Innovation of the photoacoustic patch in the fields of soft electronics and photoacoustic imaging

The innovation of this work can be summarized as the following points. In soft electronics, we developed the flexible and stretchable electronic patch that can noninvasively detect and image molecules in deep tissues. Skin-like wearable patches that integrate various kinds of sensors can monitor the health and wellness of the human body. Existing skin-like wearable patches can sense biomolecules in sweat[13-15], saliva[16,17], and tears[18-20] on the skin surface, or interstitial fluids with micro needles[21,22]. But none of the existing patches have access to those biomolecules embedded deeply underneath the skin (>1 cm). Importantly, those biomolecules in deep tissues should have a stronger and faster correlation to the dynamic processes inside the human body[23,24]. The wearable photoacoustic patch reported in this work adds an extra sensing dimension for chemical signals in the human body using soft electronics. Furthermore, our wearable patch can map and monitor core temperature in deep tissues with high accuracy and quick response. In existing literatures, core temperature can only be detected by an invasive catheter[25,26] or by heat-flux-model-based wearable temperature sensors[27,28]. However, the latter methods have slow response speed (about several hundreds of seconds) and lack capability of temperature mapping.

In photoacoustic imaging, we developed the low form factor photoacoustic patch. Conventional photoacoustic imaging systems use a laser source to generate ultrasound waves in tissues, and ultrasound transducers to receive photoacoustic waves. Laser equipment is always very bulky and heavy, not suitable for wearing. Safety regulations also require the operation of lasers by professionals. Even though some reported studies introduced the applications of laser diodes[29-32] or LEDs[33,34] as laser sources, nobody reported using vertical cavity surface emitting laser (VCSEL) bare dies, which have small thickness, ~200 micrometers, and are thus challenging to be integrated on the soft electronic platform. Furthermore, all the reported photoacoustic studies utilize bulky

ultrasound transducers to receive photoacoustic waves. Our work introduced a flexible and stretchable ultrasound transducer array as the sensing components. This work develops a flexible and stretchable photoacoustic patch by redesigning and fabricating both the laser source and ultrasound transducer elements. This work makes photoacoustic imaging possible for long-term monitoring even in freely moving subjects.

In summary, we developed a flexible and stretchable photoacoustic patch that can image molecules in deep tissues. This patch can map and monitor the core temperature in deep tissues with high accuracy and quick response. This patch is also innovative in terms of the laser source and ultrasound sensing elements of photoacoustic imaging systems, all in low form factors. None of these advances have been reported by our group or any other groups in the world.

Supplementary Note 3 | Comparison between different photoacoustic imaging systems

For a photoacoustic imaging system, the key sensing components are (1) laser sources for exciting the target molecules to generate photoacoustic waves and (2) piezoelectric transducers for detecting acoustic waves. Conventionally, the optical sources used in the photoacoustic system can be divided into three categories. The first type is the conventional high-power laser system, whose peak pulse power is usually on the order of millijoule[35,36], with a penetration depth spanning from 3 mm to 4 cm [9]. These high-power lasers are mostly used to provide strong light intensity to excite the target molecules to generate photoacoustic waves. Operating these lasers needs strict training and to be in laboratories that meet high safety standards. Additionally, these laser systems are costly and bulky, which are not suitable for wearable applications. The second type is the hand-held compact laser with a relatively lower energy than the first type. A typical laser of this kind

has a size as small as 160 mm × 64 mm × 40 mm [37]. Still, they are too large to be suitable for continuous wearing. The third type is light-emitting diodes or laser diodes. Although some photoacoustic systems employ light-emitting diodes and laser diodes as the optical source, they still rely on bulky rigid ultrasound probes to receive the acoustic waves[29,38]. Those ultrasound probes require manual holding and the subject to be static during testing. Additionally, they use edge-emitting semiconductor laser diodes, which are not suitable to be integrated into a conformal patch because the edge-emitting semiconductor laser diodes usually have a large size (more than several millimeters) in the emission direction[29].

The photoacoustic patch in this work integrates both the laser source and the piezoelectric transducer into a low form factor conformal patch (20 mm × 16 mm × 1.2 mm), by encapsulating an array of laser diode chips (1.7 mm × 2.4 mm × 0.4 mm) and transducer elements (0.6 mm × 0.8 mm × 1.0 mm) into a flexible and stretchable silicone polymer matrix. In terms of the complexity of the system, we have significantly simplified a conventional photoacoustic imaging system by replacing the bulky laser source with surface-mounted laser diode dies. Although the VCSEL chips (<$10 each) may increase the cost compared to an ultrasound patch, it greatly reduces the cost of conventionally used laser sources. In addition, the cost of each VCSEL die chip can further be reduced if the quantity of chips is increased. The stretchability of the overall patch is enabled by the serpentine shaped metal electrodes that interconnect the laser diode chips and the transducer elements. The device is rigid locally at the laser diode chips and the transducer elements but is soft globally on the system level. The penetration depth of this soft photoacoustic patch in tissues can reach >2 cm. The technology is potentially suitable for wearable health monitoring without immobilizing the test subjects.

To develop a fully integrated wearable system in the future, handling the quantity of data needs to be solved. Different clinical cases require different quantities of continuous imaging for clinical application. The monitoring period extends from several minutes to several days. Assuming each 2D image has a size of 2 cm × 2 cm, composed of 200 × 200 pixels, one 2D image would occupy ~39 KB for 1-byte unsigned integer data type. Thus, 13 slices will be ~507 KB. To be specific, continuous monitoring for 5 minutes, 5 hours, or 5 days will create datasets with the size of about 149 MB, 8.7 GB, and 209 GB, respectively. Such file sizes are easy to be accommodated since common commercial hard disks have space larger than several Terabytes. To handle these data for a completely portable system, a solution is to transfer the image data from the portable system to an external data storage equipment, which can be easily achieved by USB 2.0 cables (data transfer speed > 60 MB s^{-1}) or WiFi (data transfer speed > 2.5 MB s^{-1}).

It is worth mentioning that the big difference of optical intensity between high power lasers and laser diode chips may have influence on the detection of non-static tissues. Because the high-power laser has very strong light intensity, it can generate strong photoacoustic signals with only one pulse. Pulsation of the tissues (e.g., major arteries) will not affect the imaging result. However, for laser diode chip based photoacoustic systems, the light intensity and therefore photoacoustic signals are relatively weak. Averaging several thousands of signals are required to increase the signal-to-noise ratio. Acquiring several thousands of signals may take one second or even a longer time, during which the photoacoustic signal will move forward and back due to movements of the tissue, resulting in unstable phases of the photoacoustic signals, and thus destroying the coherent

averaging (Fig. S46)[39]. Therefore, compensation of motion will be required to achieve a good averaging result for artery imaging[40].

Supplementary Note 4 | Summary of first, second, and third optical windows

Near-infrared light has high penetration depth in human tissues compared to the visible light because of its weak scattering and absorption[41]. For probing human tissues, three commonly used optical windows are in the range of 650 ~ 950 nm, 1000 ~ 1350 nm, and 1600 ~ 1870 nm [42]. In the first window, hemoglobin still has a higher optical absorption than water and lipid. Therefore, photoacoustic signals of hemoglobin can be generated with low background noise. No extra contrast agent is needed to highlight the hemoglobin. In the second window, the penetration depth increases. But additional contrast agents are needed to label the hemoglobin molecules because of their low absorption coefficients[43-45]. The third window has even deeper penetration because of reduced scattering, but is rarely used due to the dominant water absorption[46], suppressing the detection of other molecules.

Supplementary Note 5 | Comparison between different temperature measurement methods

The gold standard for measuring the core temperature is to use a catheter to measure the temperature in the pulmonary artery[47], which is too invasive for routine measurements. Implantable devices with biocompatibility can be directly fixed in the human body, thus providing accurate and continuous temperature measurements in deep tissues[48,49]. However, in a lot of cases, the infection risks, application complexity, data communication, and power supplies of the implantable devices introduce more challenges than benefits.

There are various strategies for noninvasive temperature measurements of the human body. Wearable skin-like soft sensors usually integrate temperature sensitive electronic components, such as the thermistor[50], the ion conductor[51], and the thermocouple[52]. But they can only measure the temperature on the skin surface. Magnetic resonance imaging can quantify the internal temperature variance at a depth >10 cm and spatial resolution of 2 mm [53]. However, owing to the bulky and expensive system, it is not realistic to use MRI in daily activities.

Wearable sensors that can measure core temperatures are developed mostly based on the zero-heat-flux model[54] and the dual-heat-flux model[55,56]. In the zero-heat-flux model, when the skin and deep tissue temperatures are considered identical, there will be no heat flow between them. As a result, the core temperature is the same as the skin surface temperature[57,58]. Nevertheless, these sensors require external heaters to achieve a thermal equilibrium between the skin surface and the core body and thus have a relatively long response time (>180 s)[58], especially at a considerable depth underneath the skin. To eliminate the use of the heater, sensors based on the dual heat flux model are developed[27]. But this method requires an even longer response time (~447 s)[27] and it is imprecise since it is only a predicted value.

Compared to the existing methods, the photoacoustic patch has multiple advantages, including high penetration depth (>2 cm on tissues), short response time (~1 s), and soft mechanical design for continuous wearing.

Supplementary Note 6 | Mechanism of temperature sensing by the photoacoustic patch

Generating photoacoustic waves is a process of converting optical energy to mechanical vibration energy. After the laser illumination, biomolecules (e.g., hemoglobin in this work) will absorb the optical energy, undergo thermoelastic expansion, and radiate acoustic waves into the surrounding media. For a nanosecond laser source, the generation of photoacoustic waves satisfies the stress and thermal confinements[59]. The photoacoustic signal amplitude can be express as:[60]

$$P=\Gamma\mu_a F \tag{1}$$

where Γ is the Grüneisen parameter, μ_a is the absorption coefficient, and F is the laser fluence. During the test, the light fluence F is a constant for the same laser source. μ_a also keeps unchanged for the same type of biomolecule. The Grüneisen parameter is what changes the signal amplitude, and linear to the temperature in the range of 10 ~ 55 °C [59-61]. Therefore, the photoacoustic signal and the temperature show a linear relationship in the vicinity of human core temperature (~37 °C). The Grüneisen parameter Γ can be expressed as:

$$\Gamma=\Gamma_0+\alpha T \tag{2}$$

where Γ_0 is the value at temperature T_0, α is a constant decided by the tissue type. The photoacoustic signal amplitude can be rewritten as:[62]

$$P=(\Gamma_0+\alpha T)\mu_a F=\alpha\mu_a FT+\Gamma_0\mu_a F \tag{3}$$

The photoacoustic signal can quantify the temperature after calibrating $\alpha\mu_a F$ and $\Gamma_0\mu_a F$, which can be considered as the slope and intercept of a linear function, respectively.

Pure ultrasound techniques can also noninvasively measure the temperature in deep tissue because the tissue temperature will change the sound speed. However, there are some limitations for temperature measurements with ultrasound. First, the biggest problem is that ultrasonography can only detect the contrast of acoustic impedance, which means ultrasound collects anatomical information. As ultrasonography cannot distinguish different biomolecules, it cannot recognize the inclusion components inside cysts, which is critical for determining if the cyst is benign or malignant. Second, ultrasonography may suffer from low contrast to recognize small blood vessels. Photoacoustic imaging, as a promising biomedical imaging technique, has made a lot of advances in the last two decades[8]. Since the photoacoustic signal originates from the light absorption, photoacoustic imaging holds optical contrast, rather than the acoustic impedance contrast. In addition, photoacoustic imaging combines the best of two worlds: generating signal optically and sensing signal acoustically, which makes photoacoustic imaging best for high-resolution high-contrast imaging of biomolecules in deep tissues. Third, for temperature sensing, ultrasound has a much lower sensitivity than photoacoustics. A quantitative comparison between these two methods has been described. For instance, assuming the temperature of water increases from 20 to 30 °C, the sound speed will increase from ~1481 to ~1507 m s^{-1} [63], with a relative change of sound speed only ~0.176% per degree centigrade. On the other side, the photoacoustic signal amplitude will be enhanced by 51% for such a 10 °C increase, resulting in a relatively large amplitude change of ~5.1% per degree centigrade[60].

Supplementary Note 7 | Bland-Altman analysis

Bland-Altman plot analyzes the agreement between a pair of datasets. This plot is widely used in statistics in analytical chemistry as well as biomedicine[64] to compare a new measurement method with the gold standard[65-67]. Assuming the datasets measured by the two methods are X and Y, the *y*-coordinate of the Bland-Altman plot are the differences in each paired X and Y values, while the *x*-coordinate represents the average value of X and Y. In Bland-Altman plot, there are three horizontal lines, representing the mean bias $\bar{d}$, the upper limit of agreement E_{upper} and the lower limit of agreement E_{lower}. They are defined as follows:

$$\bar{d} = \frac{1}{n}\sum_{i=1}^{n}(\mathrm{Y}_i - \mathrm{X}_i) \tag{4}$$

$$E_{upper} = \bar{d} - 1.96 \times sd \tag{5}$$

$$E_{lower} = \bar{d} + 1.96 \times sd \tag{6}$$

where *sd* is the standard deviation. 1.96 is the boundary of the 95-confidence interval in standard normal distribution. It means that the probability of the population mean value is between -1.96 and 1.96 standard deviations.

Supplementary Note 8 | Characterization of the skin curvature on the imaging performance

To examine the influence of irregular human neck curvature on the imaging performance of the soft photoacoustic patch, the skin curvature distribution was characterized. We used a 3D scanner (HDI Advances, LMI Technologies, Vancouver, Canada) to scan the area above the internal

jugular vein (Fig. S44a). The 3D skin surface morphology was reconstructed with high spatial resolution in the software, which was then imported into Catia software (Dassault Systèmes, France) for curvature extraction. Accurate spatial positions of the skin could be read in the Catia. We acquired 26 typical 1D skin curves by placing 26 planes, with a spacing of 1 mm, vertical to the skin and extracting the intersection line between the planes and the skin surface (Fig. S44b). Then, the skin curvatures were calculated by circle fitting[68] (Fig. S44c). All of the extracted curvatures are shown in Fig. S44d. The smallest curvature radius is 6.5 cm, which corresponds to the largest deviation from an ultrasound array on a planar surface. The raw irregular skin curves were then used to decide the positions of irregularly distributed ultrasound transducer elements.

To quantify the influence of the skin curvature on the imaging performance, the generation process of the photoacoustic signals was then simulated in a MATLAB toolbox — k-Wave[69]. Seven equally distributed point sources were set at the depth of 5, 7.5, 10, 12.5, 15, 17.5, and 20 mm in human tissues. The ultrasound array of a 2 MHz center frequency was placed at the depth of 0 mm. The spatial mesh in each direction was set to be 0.05 mm, much smaller than the ultrasound wavelength of 0.77 mm to ensure high accuracy. The sampling frequency was 62.5 MHz, the same as the experimental setup. The background media was considered as breast tissues. The sound speed and tissue density were set as 1510 m s^{-1} and 1020 kg m^{-3}, respectively. The frequency dependent acoustic absorption coefficient was considered as 0.75 dB ($MHz^{y}\cdot cm)^{-1}$, where y equals to 1.5. The Coherence Factor weighted Delay And Sum algorithm was applied to reconstruct the photoacoustic images, with the ultrasound array set as a planar and a curvilinear array. Figure S45a shows the imaging results of the two scenarios side by side. The axial and lateral resolutions for the two scenarios are extracted and displayed in Figs. S45b-S45c. The results show that the average

axial and lateral resolutions are only reduced by 0.06 mm and 0.24 mm, respectively, when the transducer array moves from a planar geometry to a curved geometry without phase correction. Therefore, the irregular skin curvature had a negligible influence on the imaging performance of the soft photoacoustic patch. The reason for this negligible influence is that the working frequency of the photoacoustic patch is ~2 MHz, which is relatively low. The skin curvature radius is not small enough to produce an adverse effect for the long acoustic wavelength in this study.

Supplementary Note 9 | Feasibility of photoacoustic imaging on the detection of different biomolecules

The continuous detection of melanin could have potential applications in close monitoring metastasis of melanoma tumor cells[70]. In addition, melanoma has a very high possibility of metastasis, which causes more than 90% cancer related mortality[71]. Detection and monitoring of metastasis of melanoma tumor cells can help staging the cancer and take effective means of medical intervention at the early stage. Continuous monitoring of circulating melanoma tumor cells has been well studied[70,72,73]. Photothermal therapy has also been used to kill circulating tumors with the assistance of continuous photoacoustic imaging[73].

For the detection of glucose[74-76], cytochormes[77,78] and nucleic acid, many studies have actually demonstrated both *in-vitro* and *in-vivo* label-free imaging using photoacoustic techniques. But for now, photoacoustic imaging is not mature as a reliable technique to continuously monitor humans due to technical and regulatory challenges.

As for exogenous contrast agents, one typical example is indocyanine green (ICG), which has been approved by Food and Drug Administration due to its high biosafety[79]. ICG was not only widely used in photoacoustic imaging studies, but also well established in clinical applications in the field of other optical imaging technique[80]. Specifically for photoacoustic imaging, ICG has been used in the vena mediana cubiti of the right arm of a human volunteer to enhance the monitoring of blood haemodynamics in the finger[81]. In a much more comprehensive study, metastatic status of sentinel lymph nodes in melanoma has been detected with the administration of ICG in 20 patients[82]. The latter study demonstrates that patients can benefit from ICG-assisted photoacoustic imaging for clinical management of melanoma.

Supplementary Note 10 | Comparison of laser pulse repetition rates between different photoacoustic tomography imaging systems

Traditional photoacoustic tomography (PAT) imaging systems usually use expensive high-power lasers as the light source, such as Q-switched Nd:YAG or dye lasers[83]. They usually have a pulse energy ranging from tens of millijoules to several joules and a pulse repetition rate of around 10-100 Hz[9,70,84-90]. One laser shot can deliver sufficient light energy to generate a strong photoacoustic signal with a high signal-to-noise ratio (SNR). Therefore, without the need of data averaging, traditional PAT systems can have an imaging frame rate around 0.5~100 Hz.

However, for small-size low-cost PAT systems, expensive bulky lasers are replaced by compact inexpensive laser diodes or even light emitting diodes (LEDs)[31,38,91-94]. These light sources typically have a pulse energy from several microjoules to a few hundreds of microjoules. The laser energy per pulse is not strong enough to generate a measurable photoacoustic signal with an

acceptable SNR. Therefore, the data averaging scheme is required to improve the SNR by averaging photoacoustic signals generated by multiple laser shots. Usually, the number of averaging ranges from tens to several thousands, depending on the laser energy. To ensure a high imaging frame rate, a high pulse repetition rate is necessary for the laser diodes or LEDs to achieve the multiple data acquisition, typically in a range from several hundreds of Hz to tens of kHz. In fact, 3 kHz is a typical pulse repetition rate compared with other low-cost PAT systems.

In summary, we need to perform data averaging to improve the SNR because the laser pulse energy is low for laser diodes compared to conventional high-power lasers. To ensure a high imaging frame rate, we need to emit laser pulses at a high pulse repetition rate accordingly. For example, we need to average 3000 times of photoacoustic signals to improve the SNR. Therefore, to ensure an imaging frame rate of about 1 Hz, we have to emit 3000 laser pulses per second.

Supplementary Note 11 | Alternative denoising methods for improving signal-to-noise ratio

In this work, we adopted data averaging and bandpass filter to improve the signal-to-noise ratio (SNR) of photoacoustic signals. The bandpass filter is to eliminate the noise outside the bandwidth of the ultrasound transducers. But thousands of times of averaging makes it time consuming, causing more laser exposure. There are other methods for SNR enhancement while requiring less time consumption, as discussed in the following.

From the hardware perspective, some studies developed code excitation technique[95-98] to compensate for the low optical energy of laser diodes. Laser diode drivers control the diodes to emit light pulses at a specific sequence. The photoacoustic signal is then acquired by passing

through a match filter, which is the so-called decoding. The laser diode drivers usually require a high pulse repetition frequency to satisfy the code excitation. Although the data acquisition time of the data averaging method can also be reduced by using a high pulse repetition frequency, the code excitation scheme consumes even less time[95,97] to achieve the same SNR as data averaging.

From the signal processing perspective, there are also many denoising algorithms to improve the SNR of temporal photoacoustic signals. Typical methods include empirical mode decomposition[99,100], wavelet thresholding[101,102], Wiener deconvolution[103], and adaptive filtering[104]. Empirical mode decomposition adaptively decomposes the photoacoustic signal into a number of intrinsic mode functions, some of which represent clean photoacoustic signals and some belong to noise. Removing the intrinsic mode functions corresponding to noise based on some criteria, such as mutual information minimization[99] and energy window[100], will eliminate the noise in photoacoustic signals. Wavelet thresholding decomposes the signal into several basis functions with their own coefficients[101]. Small coefficients with the corresponding basis functions are considered as noise. Discarding the small coefficients based on hard or soft thresholding rules[101] and recombining the left basis functions with large coefficients form denoised photoacoustic signals. Wiener deconvolution models the power spectrum density of clean photoacoustic signals and additive noise. Photoacoustic signals are denoised by minimizing the expected mean squared error between the measured and clean photoacoustic signals[103]. An adaptive filtering algorithm assumes that there is at least one uncorrelated component between the measured and time-shifted photoacoustic signals[104]. This adaptive noise cancelling method does not require the prior knowledge of clean signals or noise, compared to conventional adaptive noise filters. All these denoising algorithms can be applied to the photoacoustic signals generated by one laser shot,

consuming less time than multiple data acquisition for the data averaging method. But their performance relies on the selection of specific parameters, such as the reserved number of intrinsic mode functions in empirical mode decomposition, threshold in the wavelet thresholding denoising, correlation estimation between the signal and noise in Wiener deconvolution, and time delay for adaptive filtering[104]. The data averaging method and these denoising algorithms can be adopted together to achieve better SNR[99].

Supplementary Figures

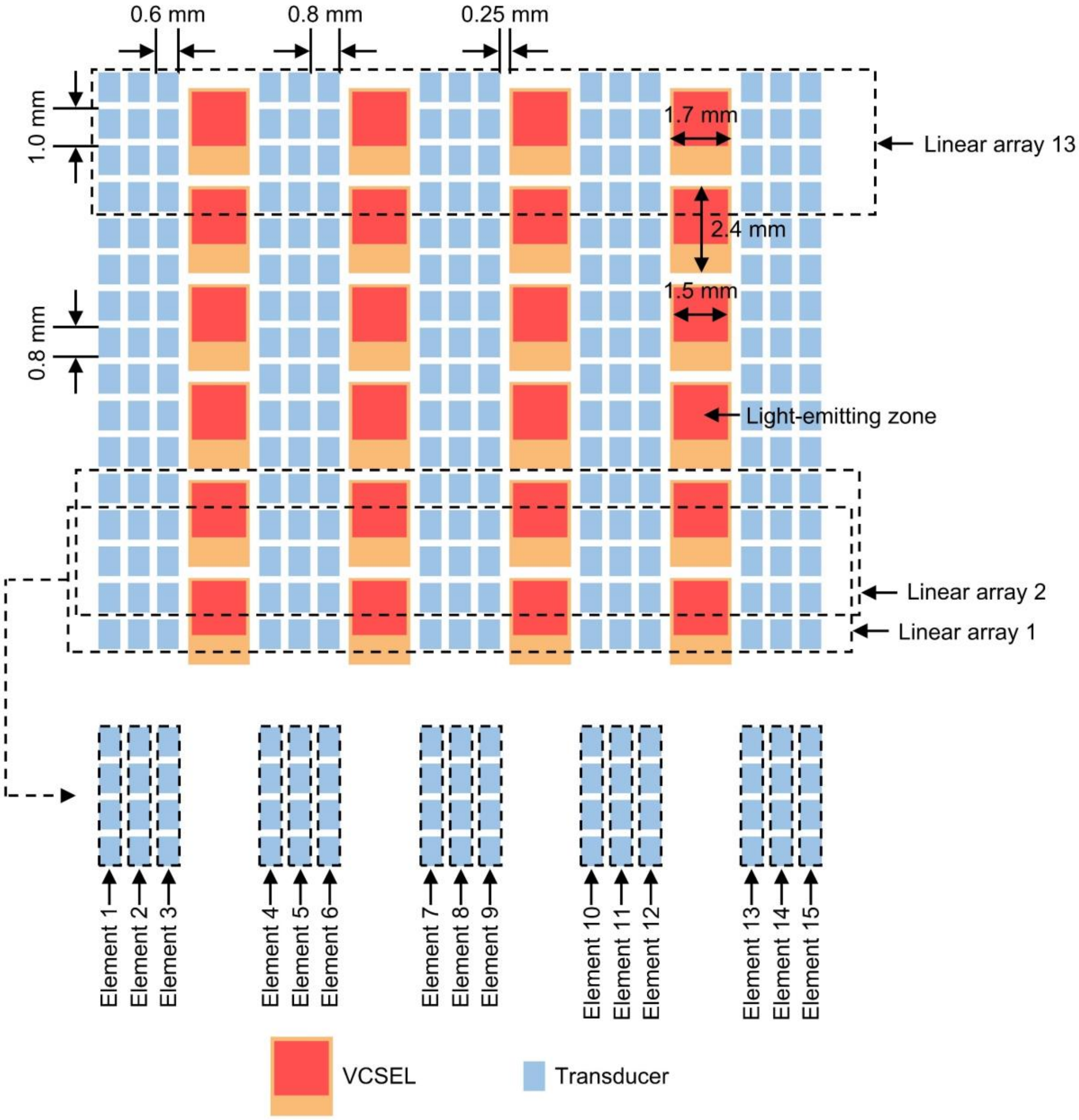

Supplementary Figure 1 | Layout design of the soft photoacoustic patch. The sizes of the laser diodes and the transducers, as well as the spacing between them, are labeled. In data processing, signals of four adjacent elements in the column are summed, digitally connected in parallel to enhance the signal amplitude. Therefore, the 16 rows of transducers form 13 linear arrays during the signal processing. In the column direction, we arrange as many VCSELs as possible to improve the optical energy intensity. In the row direction, we pack as many piezoelectric transducers as possible between VCSELs to improve the image quality. VCSEL: vertical-cavity surface-emitting laser.

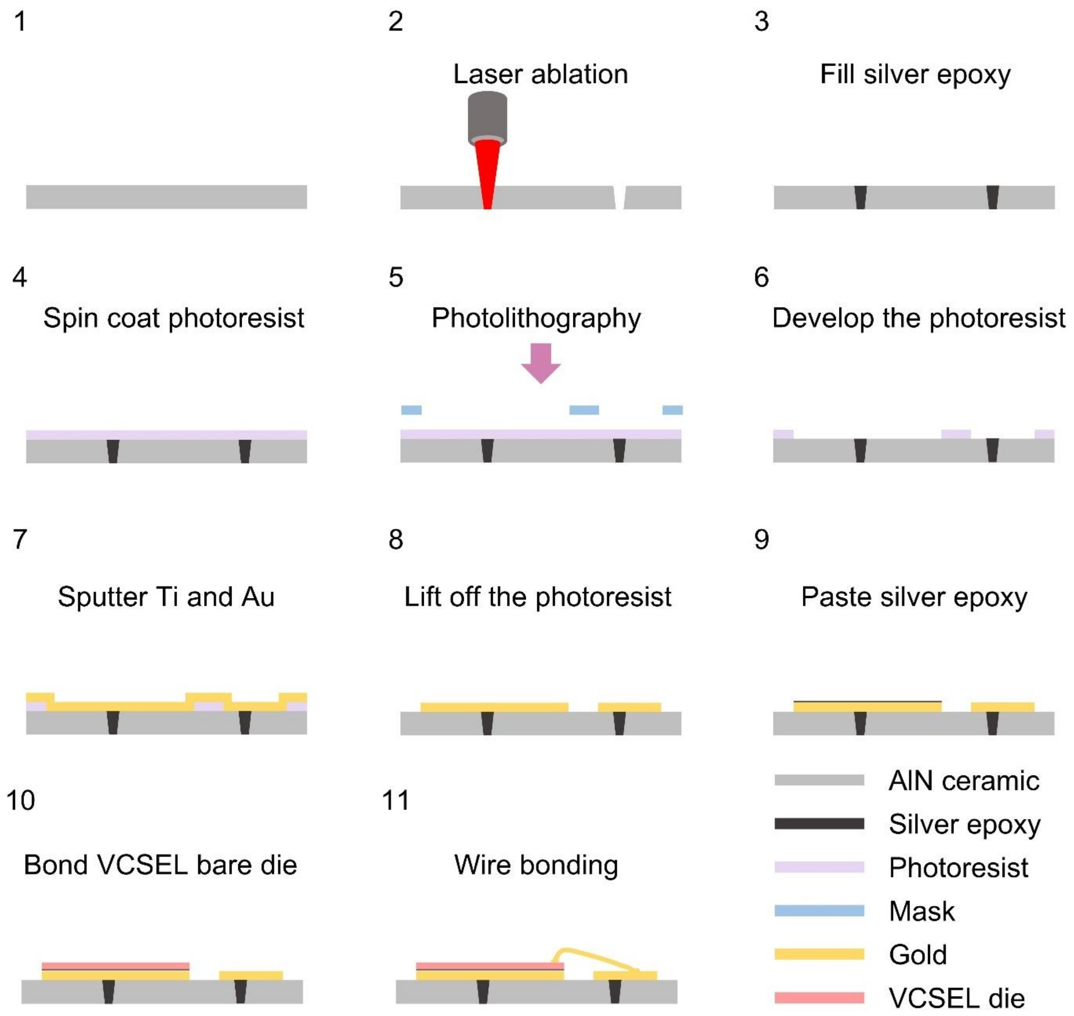

Supplementary Figure 2 | Fabrication processes of integrating the VCSEL bare die on an AlN substrate. The anode and cathode of the VCSEL bare die are on the top and bottom surfaces, respectively. We fabricate two vertical interconnect accesses in the AlN substrate and bond the VCSEL bare die by wire bonding and conductive adhesives. Then both the cathode and anode are on the bottom surface, which is readily for bonding with the serpentine Cu electrodes. VCSEL: vertical-cavity surface-emitting laser. AlN: aluminum nitride. Ti: titanium. Au: gold.

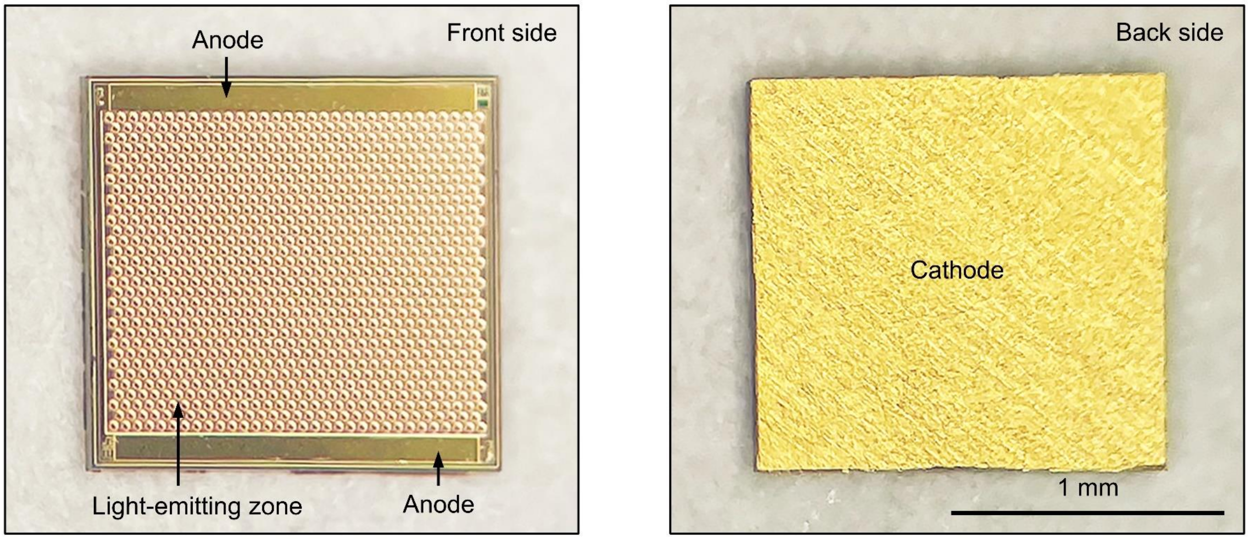

Supplementary Figure 3 | Optical photographs of the front and back sides of a VCSEL bare die. Key components of the bare die are labeled.

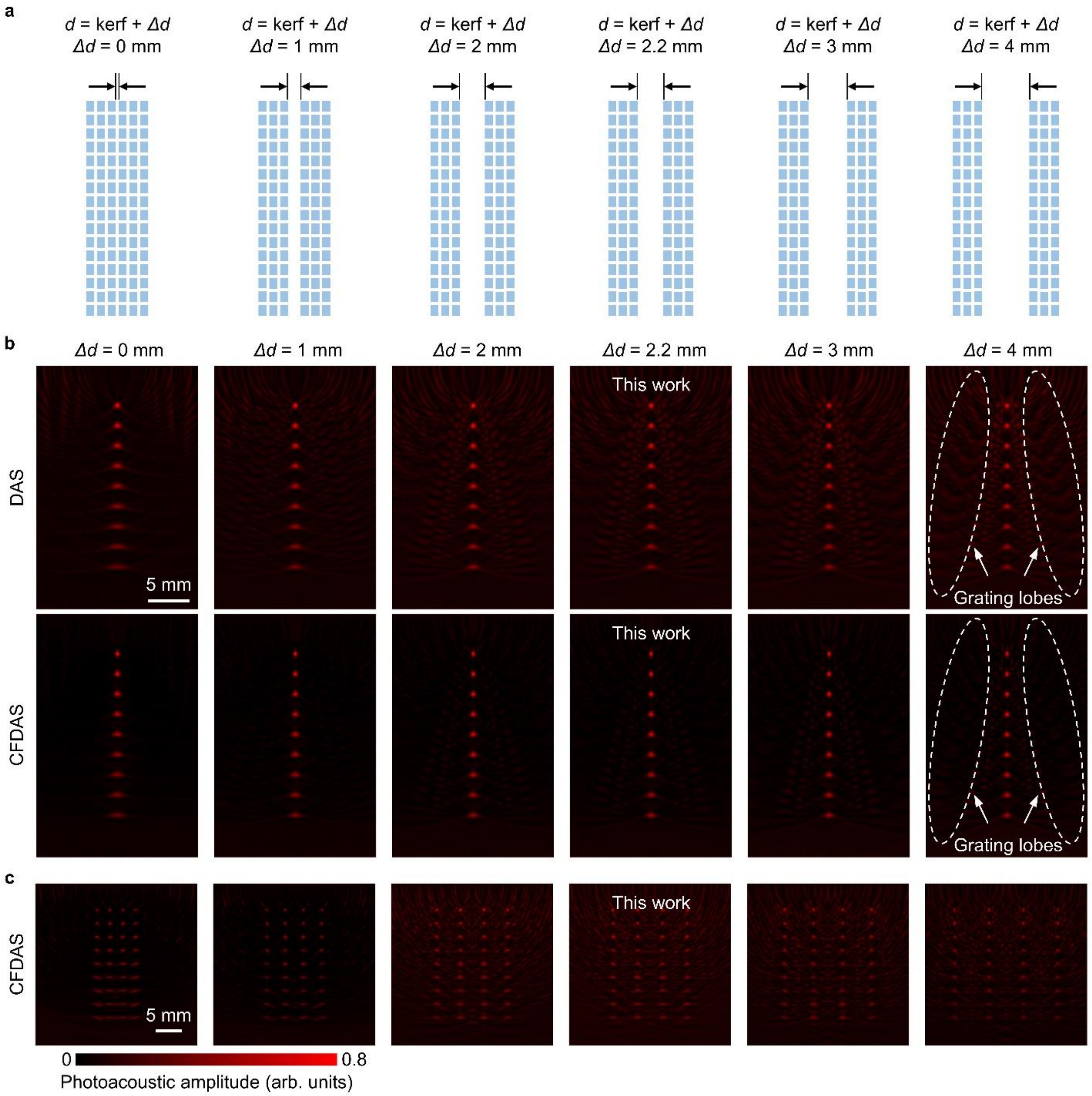

Supplementary Figure 4 | Influence of VCSEL distribution on the imaging performance. a, Schematics show how VCSEL chip increases the distance between transducers. Δd is defined as the extra distance caused by VCSEL chips between every three columns of ultrasound transducers. **b**, Simulation results show reconstructed images corresponding to different cases of Δd. Unmodified Delay-And-Sum (DAS) algorithm and Coherence-Factor-weighted-Delay-And-Sum (CFDAS) algorithm were applied to reconstruct images, respectively. When Δd equals to 0, the ultrasound transducer array has a uniform pitch between all elements. As the placement of VCSEL chips become sparser, i.e., Δd gets larger, stronger grating lobes are induced. However, the displacement of VCSEL chips also extends the aperture of the linear array, which improves the lateral resolution of photoacoustic images. Therefore, in comparison to the uniformly distributed ultrasound transducer array, VCSEL chips introduce weak grating lobes, but improve the lateral imaging resolution. For the unmodified DAS beamforming algorithm, assuming the photoacoustic signals are measured by a transducer array with M elements, the received signal of each channel

is $p_m(t)$. To reconstruct the image $I(x, z)$ at pixel (x, z), the wave propagation time from the pixel to the m-th element is Δt_m. Therefore, the image $I(x, z)$ could be computed through the summation of $\sum_{m=1}^{M} p_m(\Delta t_m)$. In the results of applying DAS to beamforming, the grating lobes become larger as Δd increases caused by VCSELs, which degrades images. Therefore, we utilize CFDAS to compensate for this effect. CFDAS introduces an adaptive coherence factor as an additional weight to $\sum_{m=1}^{M} p_m(\Delta t_m)$, which is $\mathrm{CF} = \frac{\left|\sum_{m=1}^{M} p_m(\Delta t_m)\right|^2}{M \cdot \sum_{m=1}^{M} \left|p_m(\Delta t_m)\right|^2}$. Both in ultrasound B-mode imaging[105] and photoacoustic imaging[106,107], the coherence factor weighted DAS beamforming has been demonstrated to suppress the grating lobes. The second row of images show the CFDAS algorithm decreases the impact of grating lobes. **c**, Images of points targets right underneath the VCSELs reconstructed with CFDAS algorithm. All the images share the same color map, ranging from 0 to 0.8. The images of all targets under the VCSEL have high axial and lateral resolutions, which are only slightly affected by the increased Δd. Furthermore, the intensities of the targets only decrease slightly.

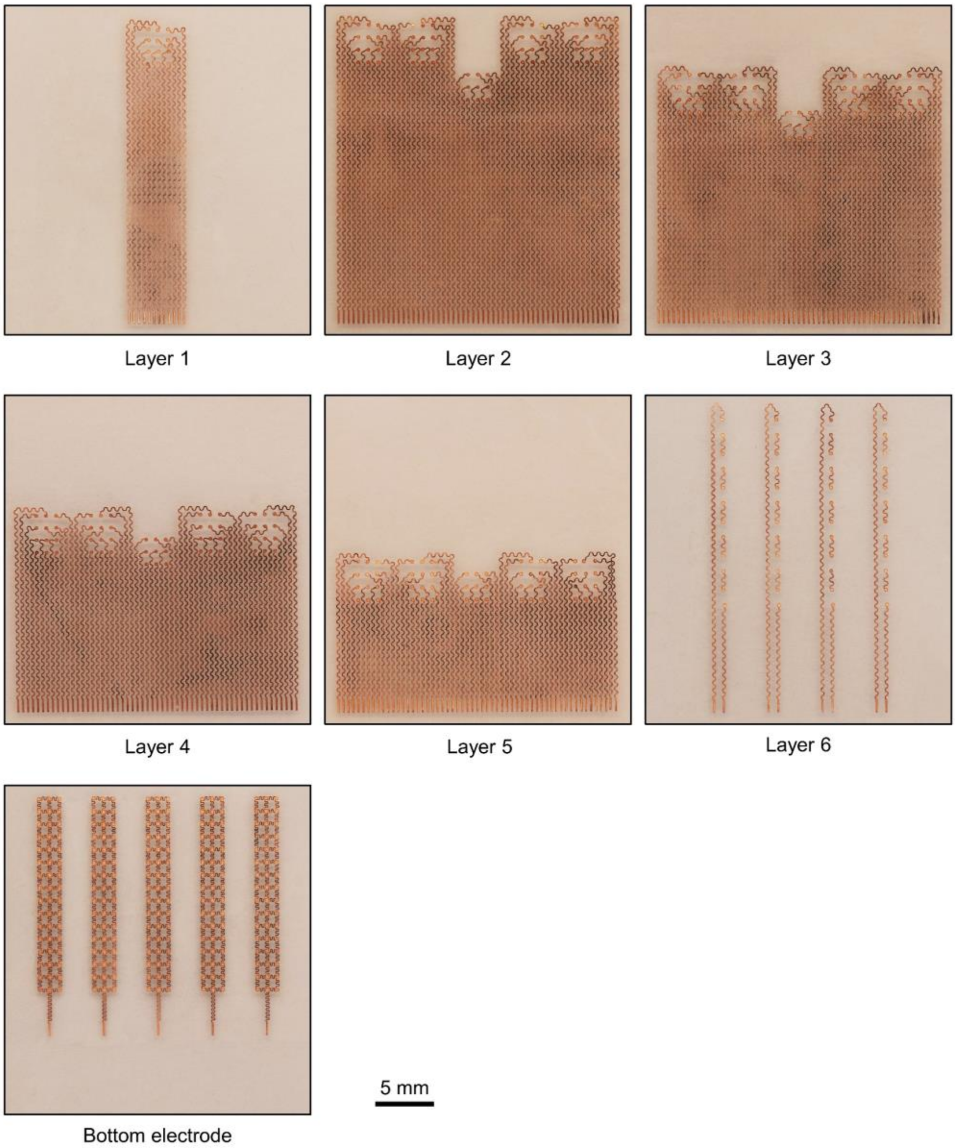

Supplementary Figure 5 | Optical photographs of all the seven layers of Cu electrodes. The first six layers form the top electrodes, among which layer 6 is for the VCSELs, while others are for the piezoelectric transducers. The bottom electrode is the common ground for all the transducers.

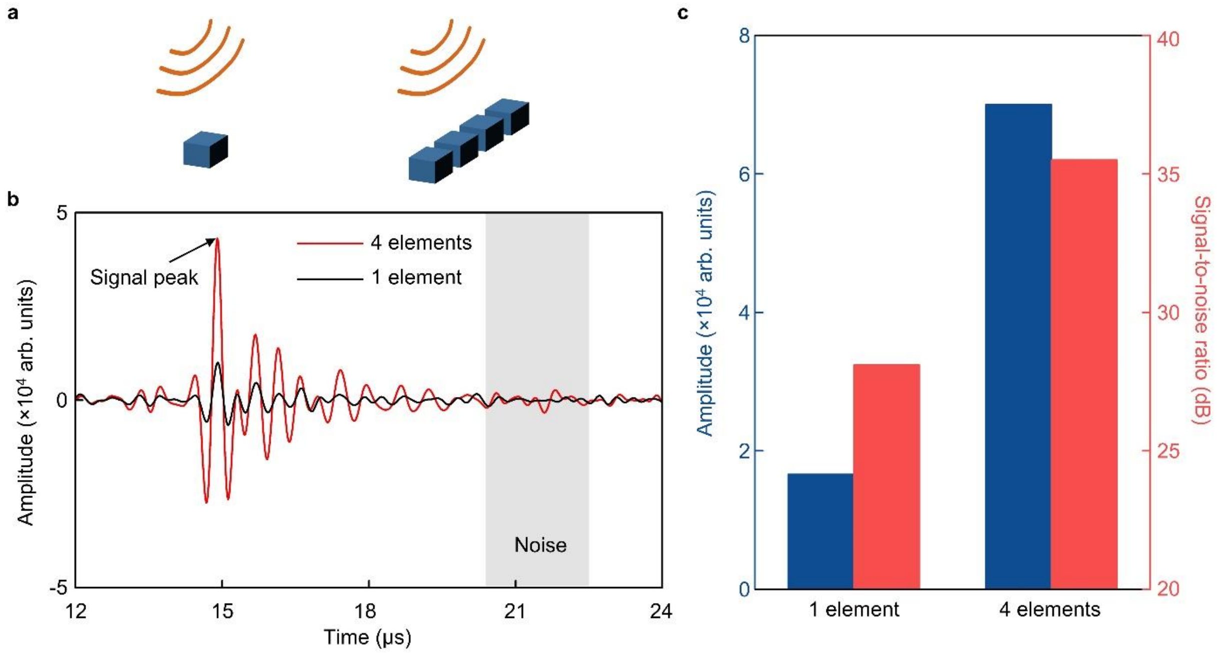

Supplementary Figure 6 | Performance comparison between one element and four parallelly connected elements. a, Schematics of the two measurement strategies. **b**, The time domain photoacoustic signals received by one element and four elements. **c**, Comparison of signal amplitudes and signal-to-noise ratios between one element and four elements. Signal-to-noise ratio is defined as 20log10(Peak photoacoustic signal/Root mean squared error of noise)[108-110].

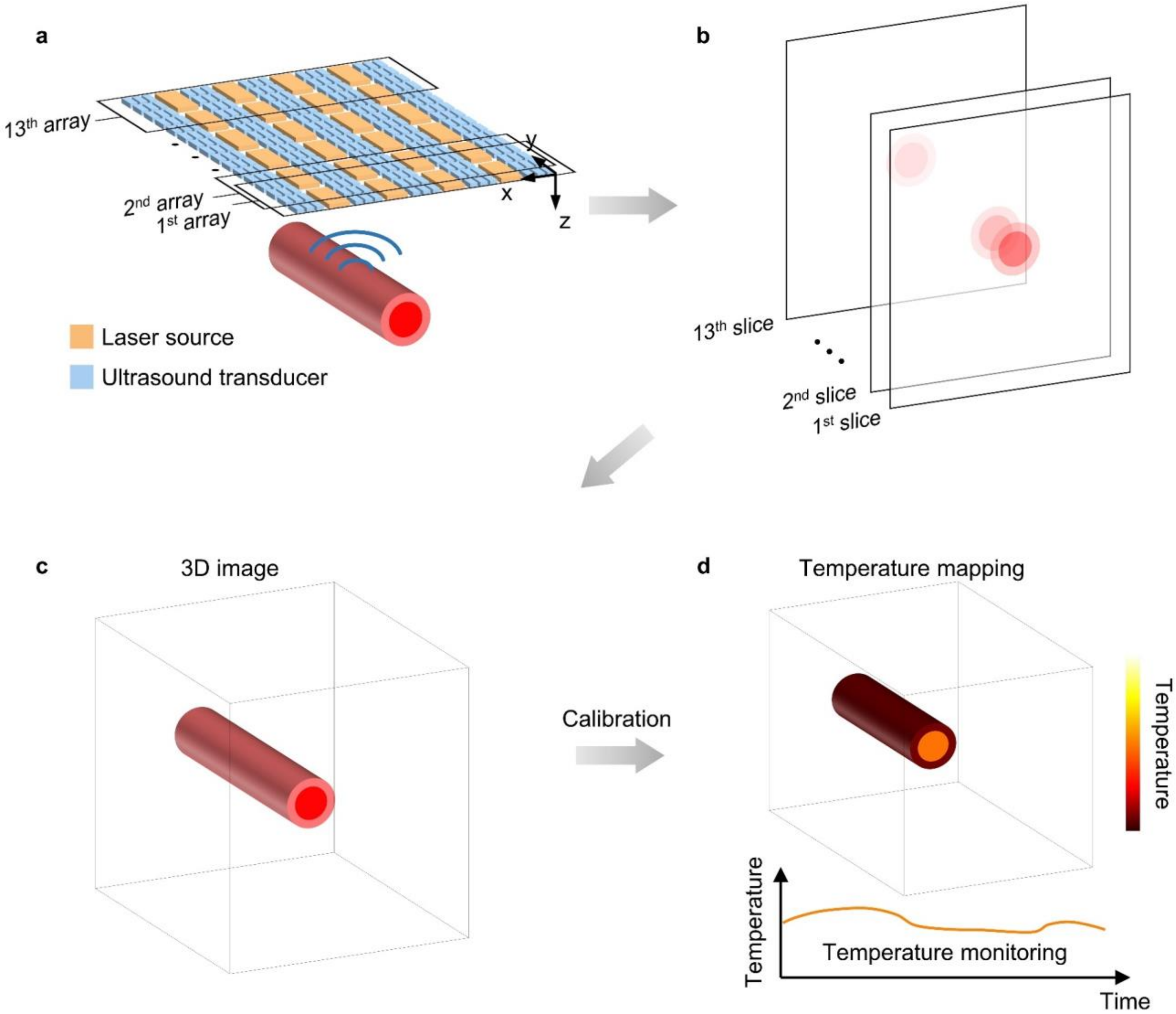

Supplementary Figure 7 | Workflow of the photoacoustic patch. a, Signals received by four elements in the *y* direction are summed to enhance the signal-to-noise ratio. 13 linear arrays in total are formed equivalently. **b,** 13 slices of 2D images are reconstructed by the patch. **c**, A 3D image is formed based on the 13 slices of 2D images. **d**, After calibration, 3D temperature mapping is achieved, which is the basis for the continuous core temperature monitoring.

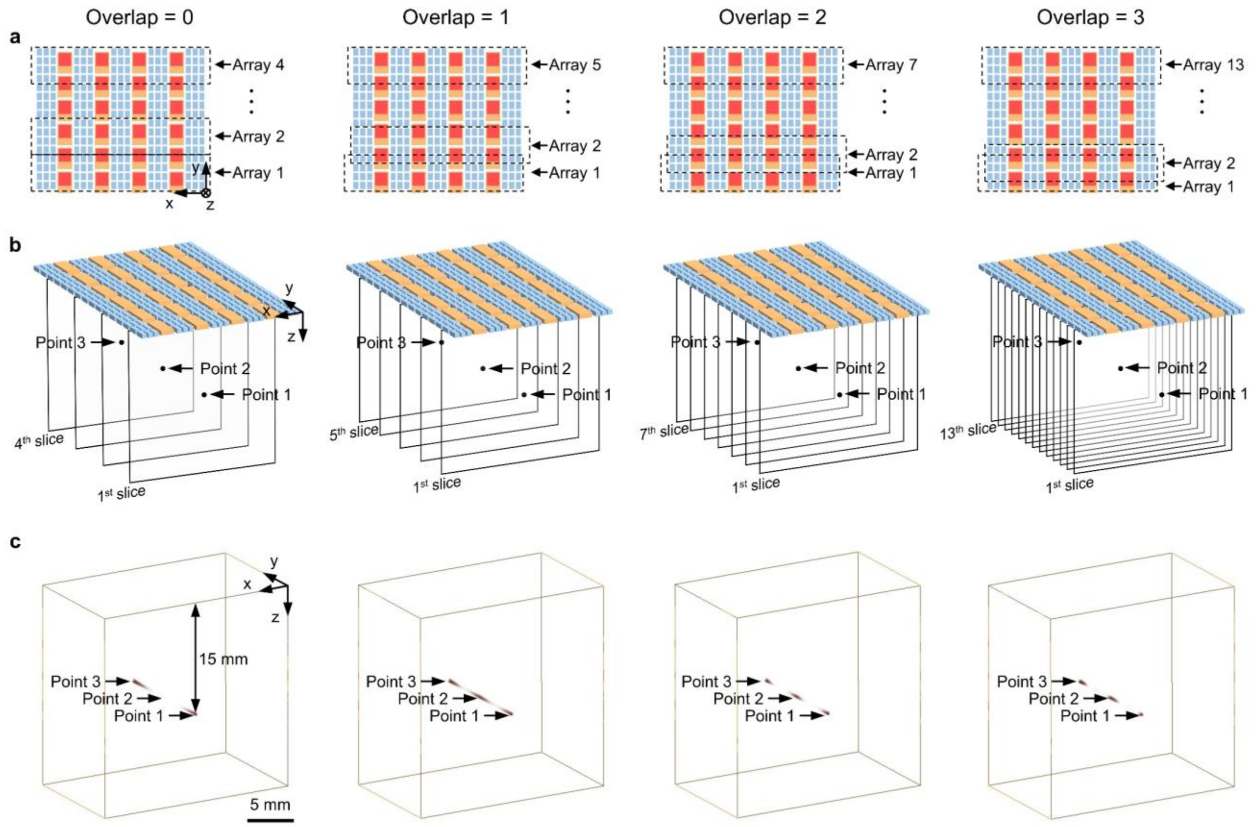

Supplementary Figure 8 | Influence of overlap of ultrasound transducers. a, Schematics showing four cases with the different numbers of overlapped transducers between adjacent linear arrays. **b,** Imaging planes corresponding to different setups. Three uniformly distributed point sources were set at the depth of 1.5 cm. **c,** Reconstructed 3D images for different cases. Because of the overlap between adjacent arrays, we can have a large number of linear arrays, which increases the number of 2D images in the *y* direction. The 3D images show that when there is no overlap, we only have four 2D images. Not all the point sources can be recognized in the 3D image. As the overlap increases, the number of 2D images increases. All points sources are captured. Furthermore, the width of the points in the *y* direction decreases as the overlapping increases, which means the lateral resolution in the *y* direction is improved. In summary, increasing the number of overlapped transducers increases the number of 2D images, improving the lateral resolution in the *y* direction.

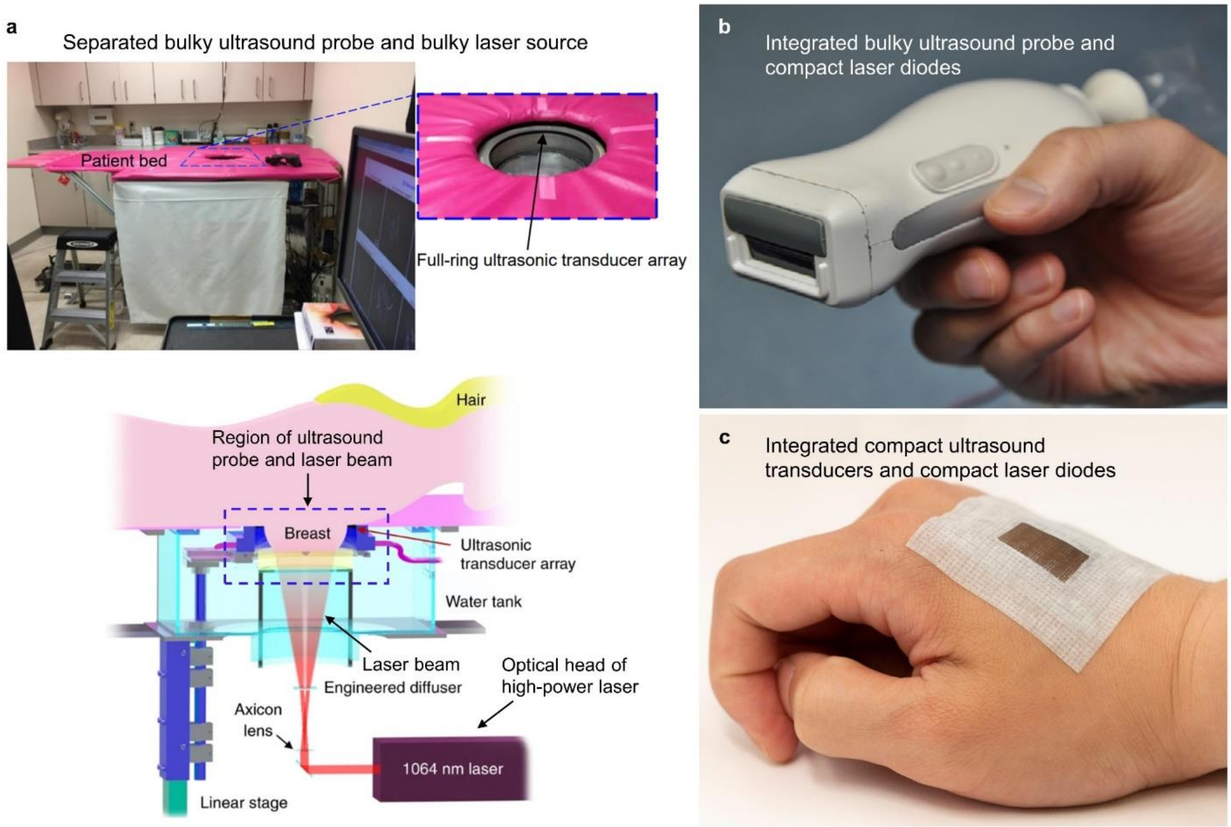

Supplementary Figure 9 | Three typical photoacoustic tomography imaging systems. a, Conventional PAT system using bulky ultrasound transducers and expensive bulky high-power lasers[9]. This PAT system has the largest size because the ultrasound probe and the laser are both bulky, immovable, and physically separated. The laser beam is usually guided by optical fibers or lens to illuminate human tissues. The size of the laser is usually over tens of centimeters. The image is adapted from REF[9], which is licensed under a Creative Commons Attribution 4.0 International License (http://creativecommons.org/licenses/by/4.0/). We added several labels to illustrate the equipment. **b**, Alternatively, a low-cost photoacoustic probe integrating a traditional bulky ultrasound probe and compact laser diodes[32]. The laser diodes are installed in the ultrasound probe and only need to be connected with an external compact laser diode driver. The integrated photoacoustic probe is handheld and movable. This PAT system reduces the size of the whole system because the laser diodes have much smaller size compared to conventional high-power lasers. The image is copied from REF[32], which is licensed under the CC BY-NC-ND license (https://creativecommons.org/licenses/by-nc-nd/4.0/). No changes were made. **c**, Wearable photoacoustic patch integrating low form-factor ultrasound transducers and vertical-cavity surface-emitting laser (VCSEL) diodes. To date, this kind of design has the smallest size in the literature because we greatly reduced the sizes of both ultrasound transducers and laser sources. The laser diodes only need to be connected to an external compact laser diode driver. The patch can be attached to the skin with medical tape, allowing hands-free monitoring.

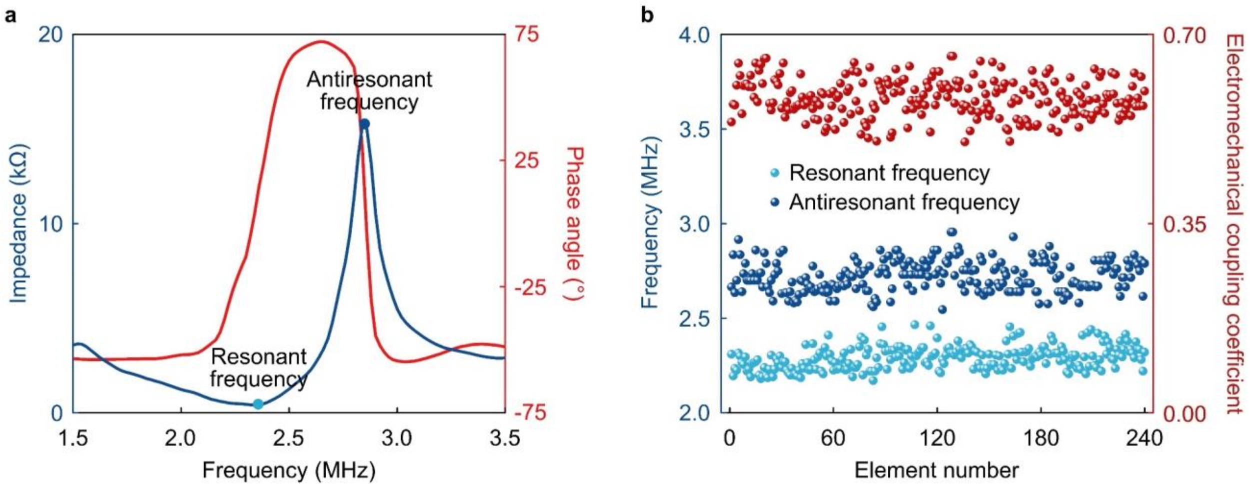

Supplementary Figure 10 | Characterization of the piezoelectric transducers. a, The impedance and phase angle of a typical piezoelectric transducer element. The resonant and antiresonant frequencies have been labeled. **b,** Resonant frequency, antiresonant frequency, and electromechanical coupling coefficient of all the 240 transducer elements, showing the consistency of the fabrication process.

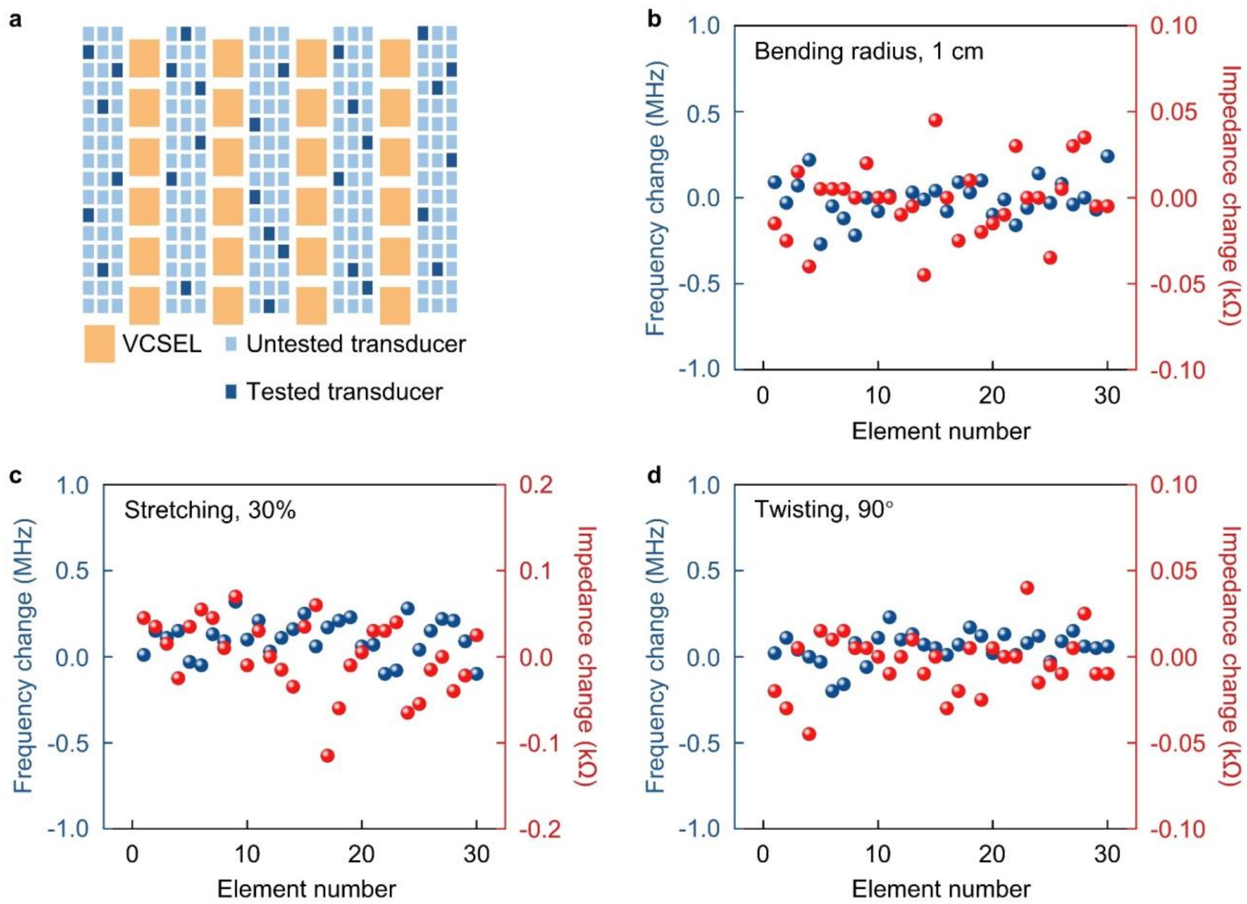

Supplementary Figure 11 | Influence of mechanical deformations on the transducer impedance. a, Distribution of representative transducer elements measured under mechanical deformations. Changes in resonant frequencies and impedance when **b,** bending, **c,** stretching and, **d,** twisting the soft photoacoustic patch. Those deformations have minimal impact on the transducer impedance.

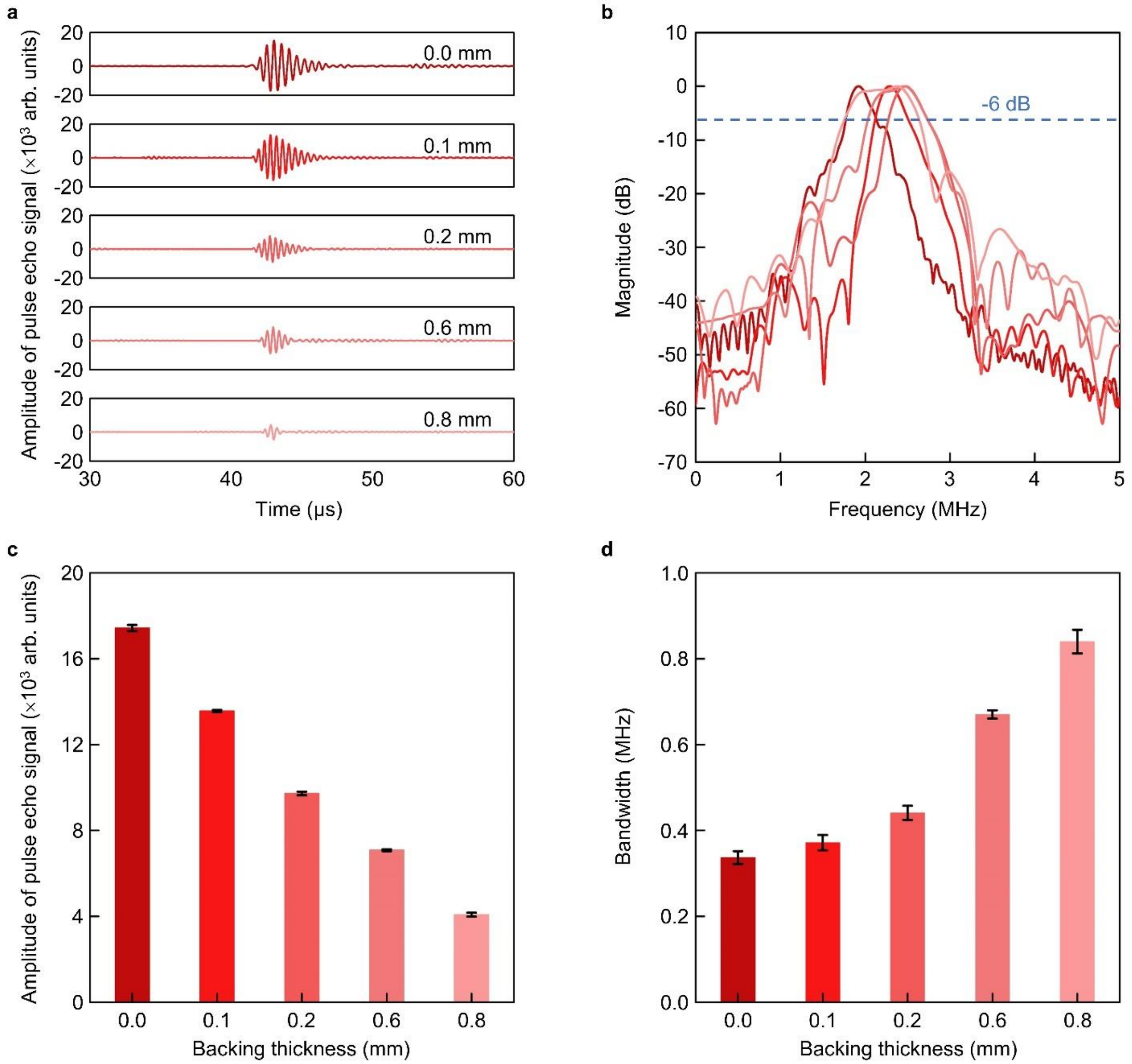

Supplementary Figure 12 | Influence of backing layer thickness on the transducer performance. The backing layer decreases the signal amplitude if too thick. We need to make sure the transducers have high sensitivity to receive weak photoacoustic signals, and small thickness not to affect the flexibility and stretchability of the patch. These two factors require the thickness of the backing layer to be as small as possible. On other hand, we still need the backing layer to dampen excessive vibrations to improve the signal bandwidth. In our design, high sensitivity and high flexibility are more important than high bandwidth. To measure the relationships among bandwidth, signal amplitude, and the thickness of the backing layer, we fabricated five different transducer elements, which have the same size of 3 mm × 3 mm, but different thicknesses of the backing layer: 0, 0.1, 0.2, 0.6, and 0.8 mm. We excited each element with the same voltage of 50 V and measured the pulse echo signal of each element reflected by an aluminum block. **a**, Time-domain pulse echo signals of different transducers. **b**, Frequency-domain pulse echo signals. **c**, The relationship between the signal amplitude and backing layer thickness. Data are presented as mean values +/- standard deviation of 20 measurements. **d**, The relationship between the -6dB bandwidth and backing layer thickness. Data are presented as mean values +/-

standard deviation of 20 measurements. The results show that the amplitude decreases and the bandwidth increases, when the thickness of backing layer increases. In this study, we set the backing thickness as 0.2 mm.

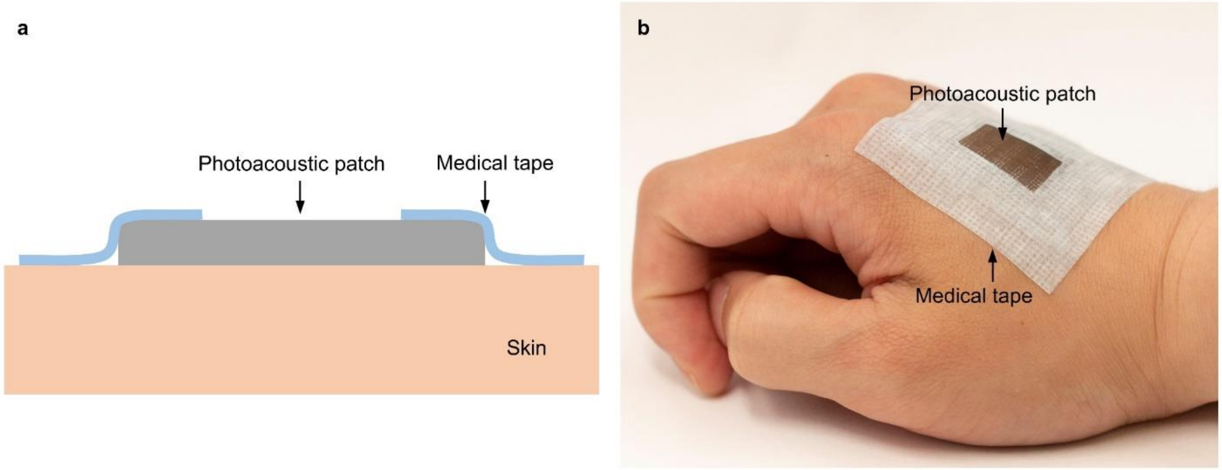

Supplementary Figure 13 | Conformal attaching of the photoacoustic patch to the skin. a, A schematic cross-sectional image of attaching the photoacoustic patch to the skin by using a medical tape. The photoacoustic patch is fabricated by encapsulating ultrasound transducer elements and laser diodes in flexible and stretchable silicone elastomer, i.e., Ecoflex in this work. The ultrasound transducer elements and laser diodes are connected by stretchable serpentine electrodes, in an island-bridge structure. The photoacoustic patch is rigid locally at each ultrasound transducer element and laser diode but soft globally on the system level. Therefore, no external pressure is required to conformally attach the photoacoustic patch to the skin. **b,** A photo shows attaching the photoacoustic patch on the hand with only a medical tape.

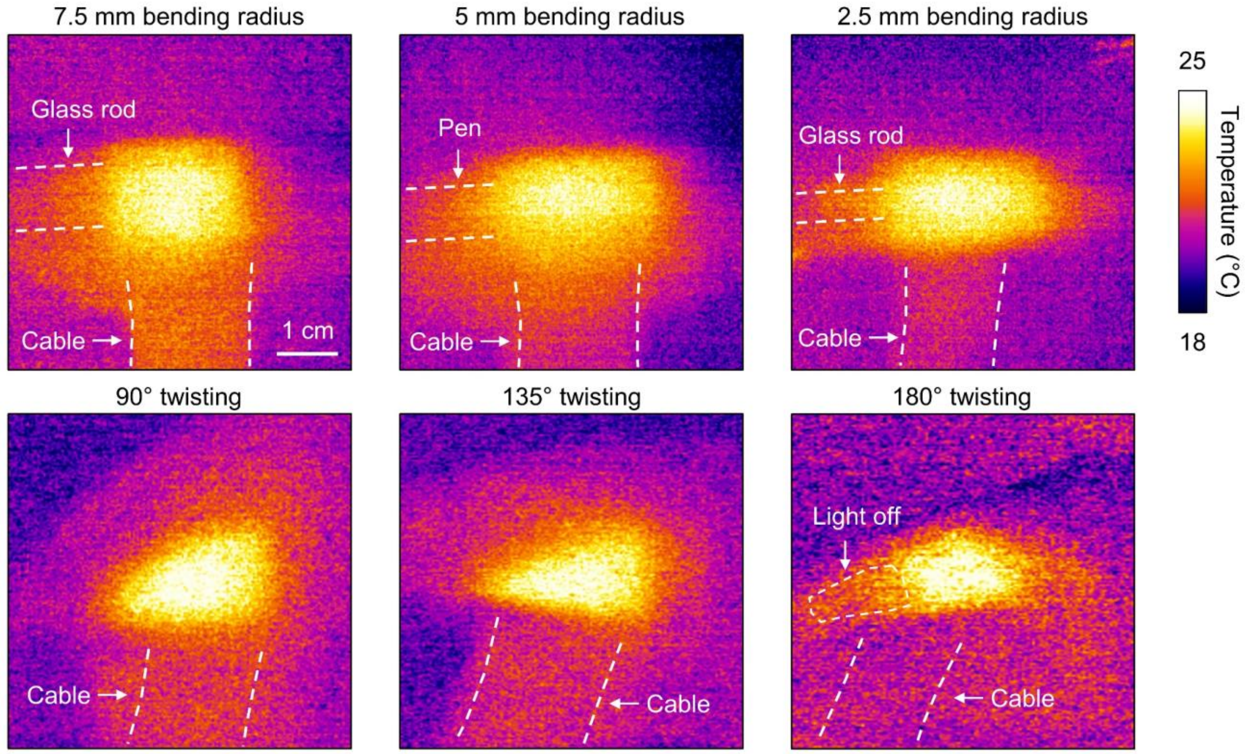

Supplementary Figure 14 | Infrared camera images of the photoacoustic patch under different degrees of bending and twisting. The patch can survive a bending radius of 2.5 mm and a twisting angle of 135°.

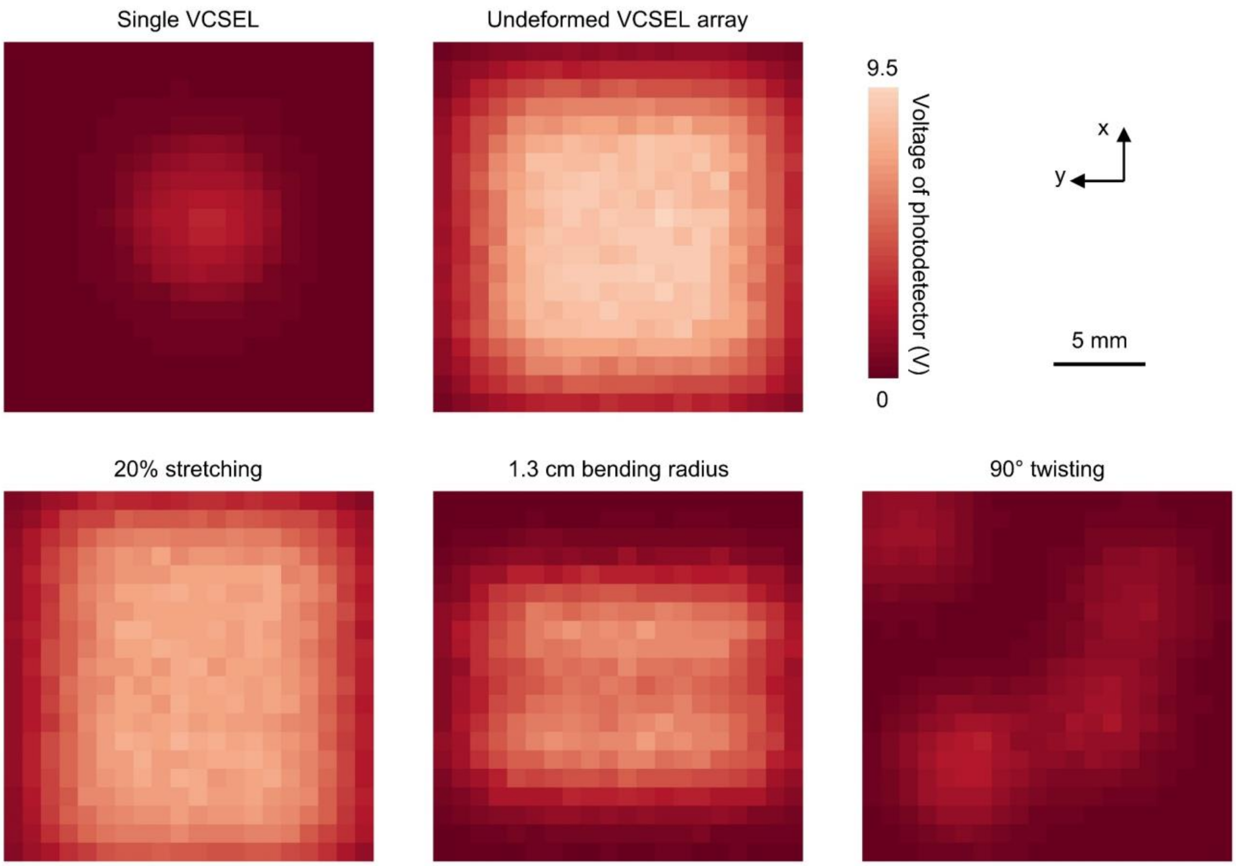

Supplementary Figure 15 | Light intensity distributions of a single VCSEL, and an undeformed, stretched, bent, or twisted VCSEL array. The optical intensity distribution under 20% uniaxial stretching is very similar to that of the undeformed state. Under large degrees of bending and twisting, the light distribution will be distorted.

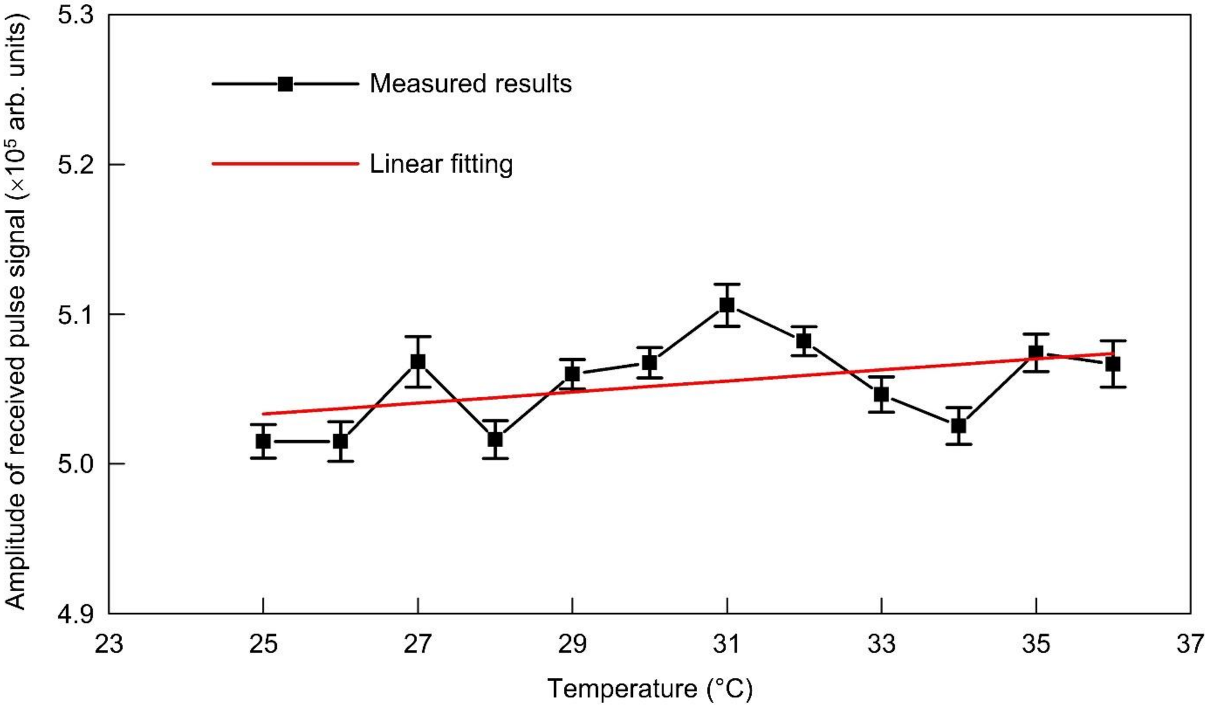

Supplementary Figure 16 | Tested relationship between the receiving sensitivity of piezoelectric transducers and temperature. The patch and a commercial ultrasound probe (Verasonics, P4-2v) were immersed in water directly opposite to each other. The water's initial temperature was about 40 °C. The commercial probe was excited by a voltage pulse at a transmitting frequency of 2 MHz, while the photoacoustic patch received the ultrasound signals. A thermocouple (Omega Engineering Inc., SC-TT-K-30-36) was immersed in water to simultaneously measure the water temperature. Signals received by the ultrasound transducer in the patch were recorded at different temperatures from 25 °C to 36 °C, which was the temperature range of the photoacoustic patch after being turned on. The signals' peak-to-peak amplitudes are shown here. The square points are the average of 20 measurements, while the error bars are the stand deviations. The red line is the linear fitting of the average amplitudes. As shown, there is fluctuation at different temperatures. The highest amplitude is about 1.8% higher than the lowest, which can be further reduced by averaging more measurements and transducers. The fitting result shows a 0.8% increase of signal amplitude as the temperature increases. This increase is very small, because the photoacoustic signal typically increases about 5% per degree centigrade[60]. In addition, the temperature of photoacoustic patch reaches a stable state after being turned on for about 8 minutes. We can easily avoid the influence of temperature on the receiving sensitivity if we start the long-term monitoring from 8 minutes after turning on the laser diodes.

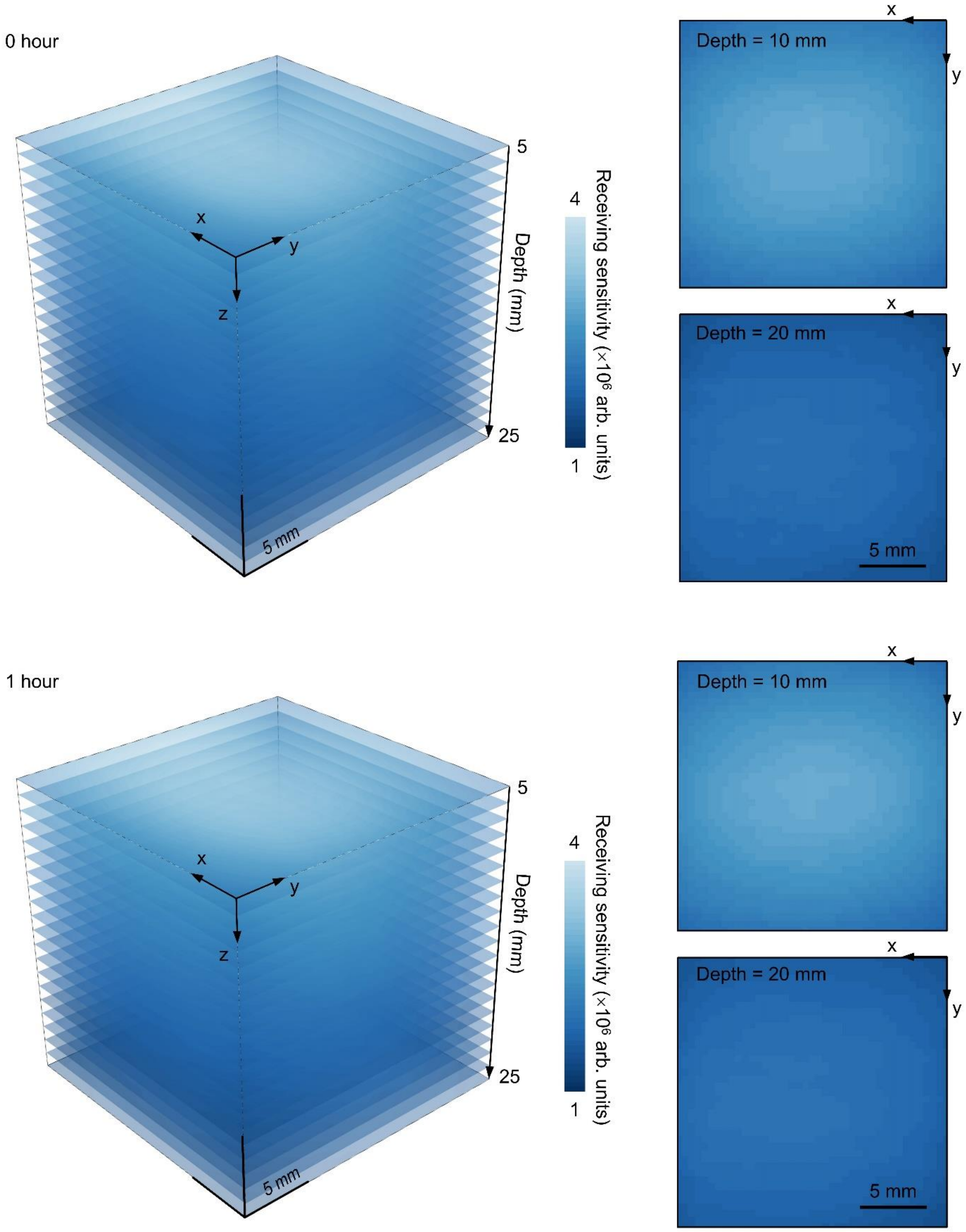

Supplementary Figure 17 | Measured receiving sensitivity mappings of the photoacoustic patch at 0 hour and 1 hour, respectively. The 3D image shows the mappings at 21 depths from 5 mm to 25 mm. Two horizontal planes at the depth of 10 mm and 20 mm are also shown on the right, respectively. The receiving sensitivity of the photoacoustic patch was measured in a water tank (ONDA, AIMS III). The results at two moments are very close, which may be because the

photoacoustic patch was immersed in water. The heat was dissipated into the water tank. However, according to the analysis in Supplementary Figure 16, the receiving sensitivity of the ultrasound transducers will not change too much either, even when the water temperature rises from 25 °C to 36 °C. Therefore, the dependency of receiving sensitivity on the temperature is not a concern in this study.

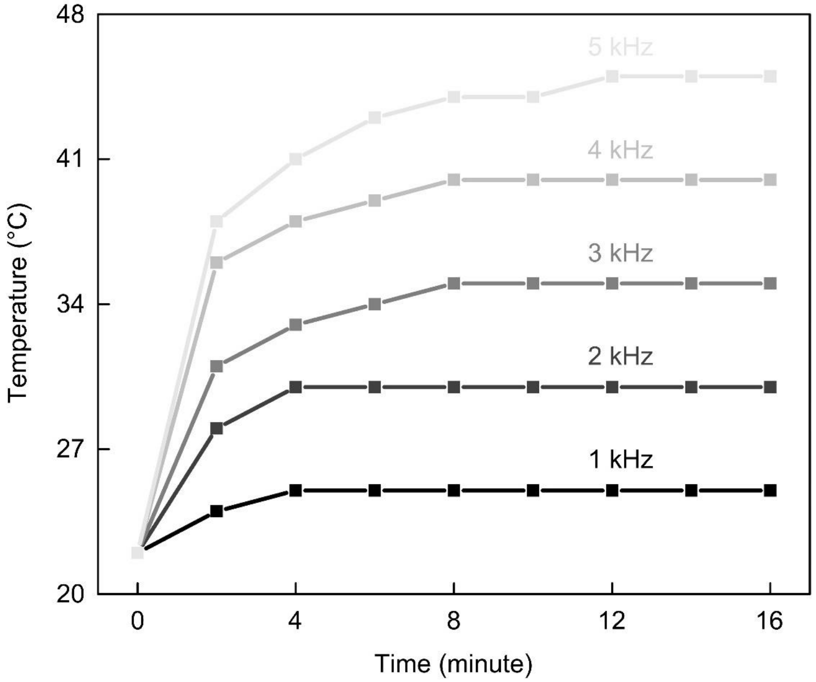

Supplementary Figure 18 | Influence of the pulse repetition frequency of the VCSELs on the overall patch temperature. The temperature increases as the pulse repetition frequency increases. Under a given pulse repetition frequency, the patch temperature gradually rises within the first few minutes and then stabilizes. 3 kHz is used in this study.

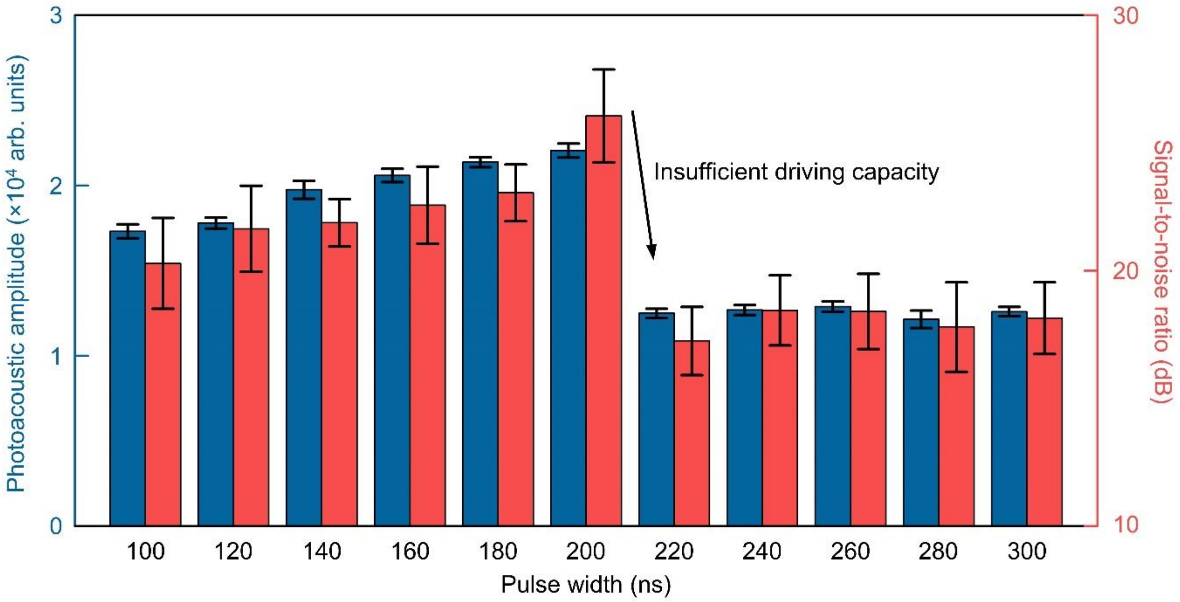

Supplementary Figure 19 | Influence of laser pulse width/duration on the photoacoustic signal amplitude and signal-to-noise ratio. The photoacoustic signal amplitude and signal-to-noise ratio increase as the pulse duration when the duration is below 200 ns. When the duration is above 200 ns, the laser diode driver (PicoLAS) used in this work cannot support further increased pulse duration due to its limited power. Data are presented as mean values +/- standard deviation of 20 measurements.

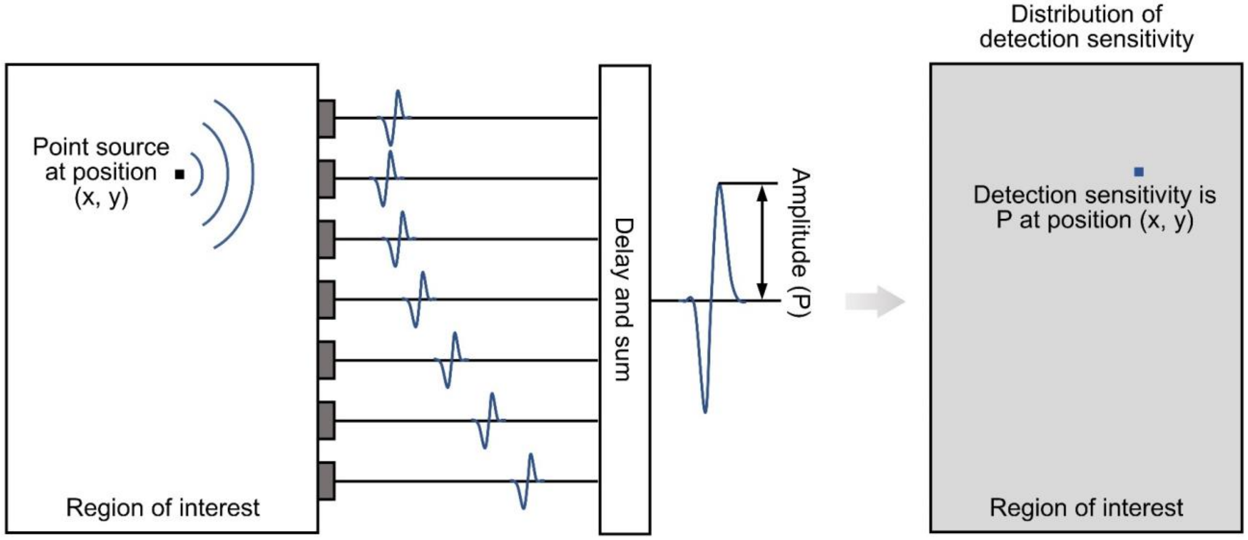

Supplementary Figure 20 | The method of calculating detection sensitivity. Assuming there is a point source at the position (x, y), the amplitude of beamformed signals (P) is set as the detection sensitivity of this position illustrated in Fig. 2d.

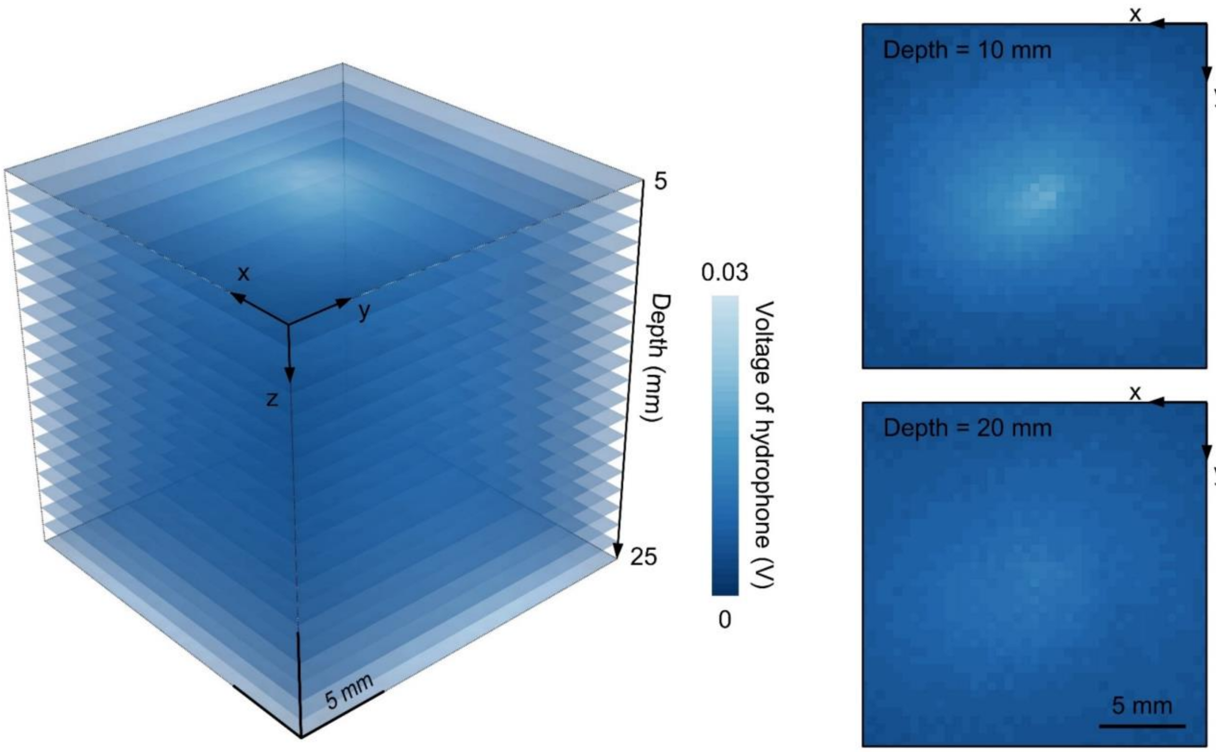

Supplementary Figure 21 | Measured transmitting sound field of one transducer element. The 3D image shows the mappings at 21 depths from 5 mm to 25 mm. Two horizontal planes at the depth of 10 mm and 20 mm are shown on the right, respectively. The figure shows the 3D ultrasound field underneath the transducer at a depth from 5 mm to 25 mm, with a horizontal area of 2 cm × 2 cm.

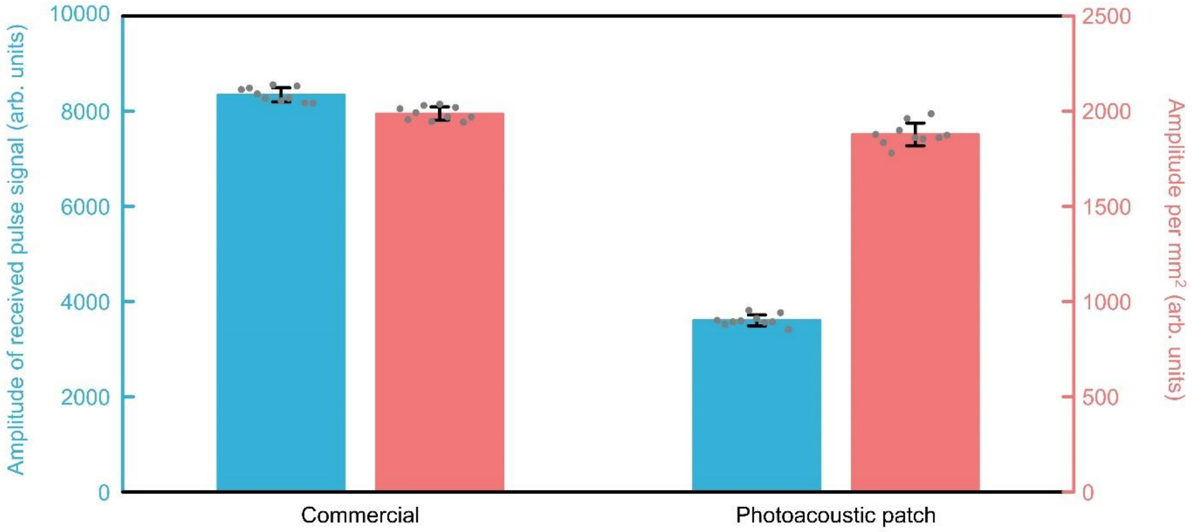

Supplementary Figure 22 | Comparison of receiving sensitivity between commercial ultrasound probe and the photoacoustic patch. Blue and red bars show the sensitivity without and with considering the area of transducer element. The sensing areas of one transducer were 4.2 mm^2 and 1.92 mm^2 for the commercial probe and the patch, respectively. Because the amplitude of measured signal increases as the transducer area, we also calculated the signal amplitude per square millimeter. The results are the averages of 10 measurements. The error bars denote the standard deviation. Gray dots show the data of 10 measurements. The blue column bars show that the commercial probe has higher signal amplitude than the patch. Considering the difference in the transducer area, the red bars show that the amplitude of the patch is only about 11% lower than the commercial probe, which means the patch has a very similar performance to the commercial probe.

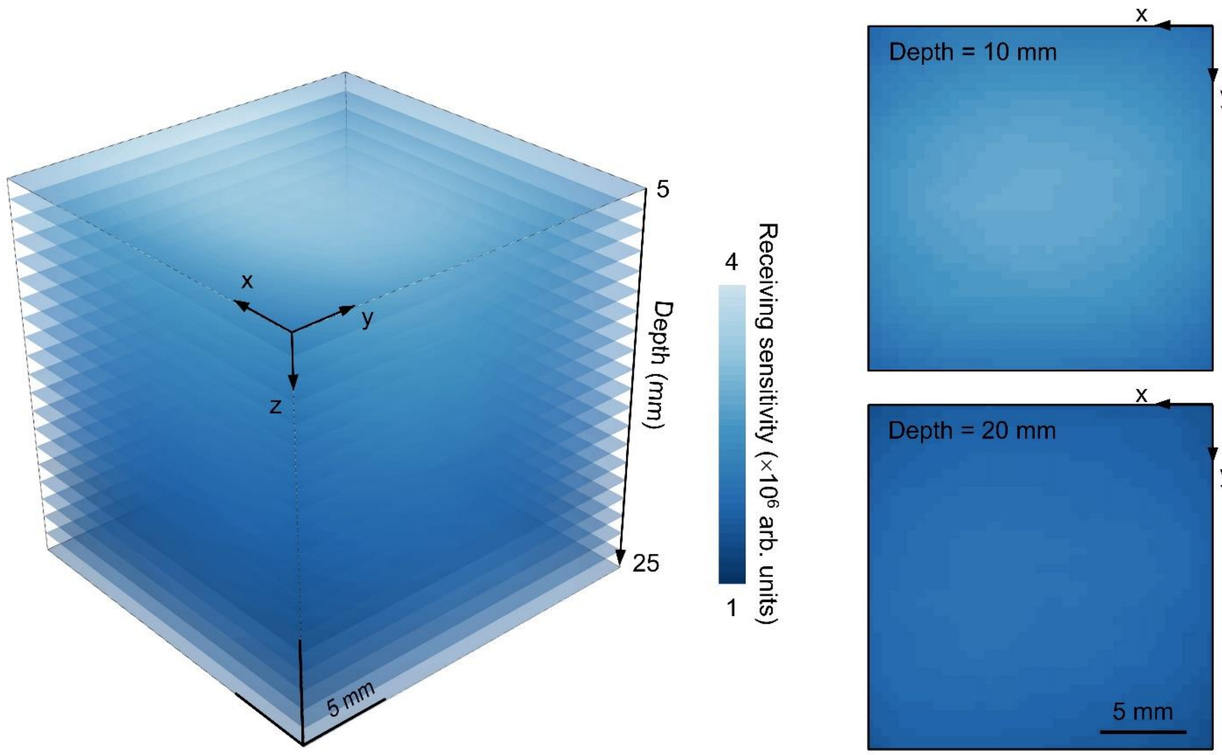

Supplementary Figure 23 | Measured receiving sensitivity mapping of the photoacoustic patch. The 3D image shows the mappings at 21 depths from 5 mm to 25 mm. Two horizontal planes at the depth of 10 mm and 20 mm are shown on the right, respectively. The results show the normalized 3D sensing field underneath the patch at a depth from 5 mm to 25 mm, with a horizontal area of 2 cm × 2 cm.

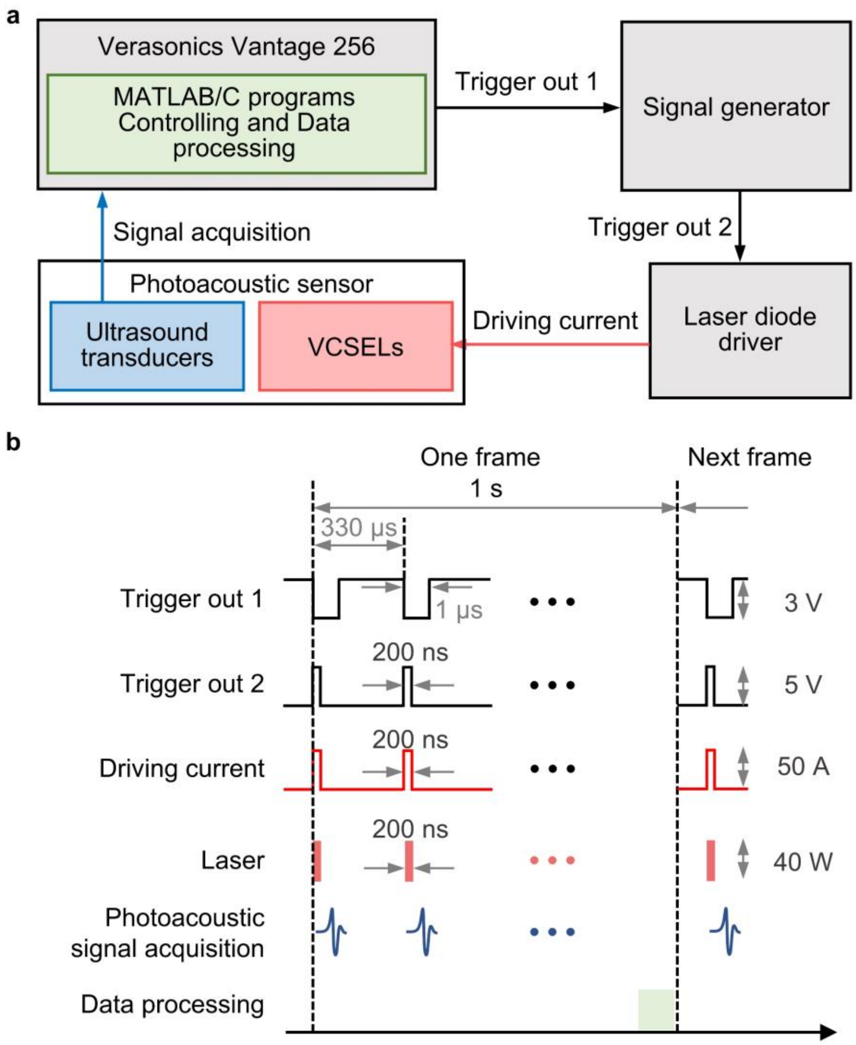

Supplementary Figure 24 | Schematic framework of the photoacoustic system. a, The hardware framework of the system. The Verasonics Vantage 256 controls the timing sequence of the entire system through a customized MATLAB program. It outputs a trigger signal to the signal generator, which then exports a pulsed trigger signal to the laser diode driver. With the driving current, the laser diodes will emit laser and excite the hemoglobin molecules to generate photoacoustic waves. At the moment of laser emission, the transducers start to measure photoacoustic waves and relay the signals to the Verasonics for processing, which is done by customized MATLAB and C programs. **b,** Timing sequence of the system. The pulse repetition frequency of laser emission and signal receiving is 3 kHz. One frame of image is reconstructed based on 3000 averaged signals in one second. The frequency of measured photoacoustic signal is mainly decided by the laser pulse width and the bandwidth of the piezoelectric transducer.

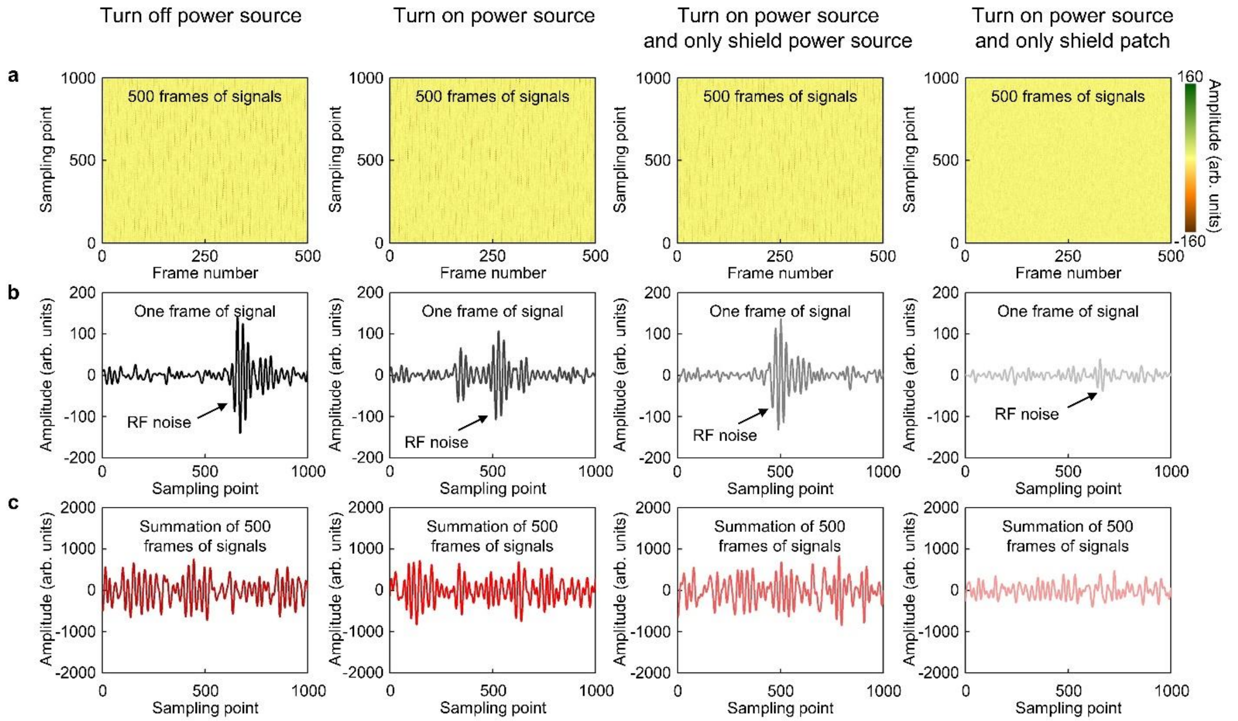

Supplementary Figure 25 | Influence of external radiofrequency (RF) noise. We did four groups of tests to show the influence of RF noise, including 1. turning off the power supply and function generator; 2. turning on the power supply and function generator, applying no shielding; 3. turning on the power supply and function generator, and only shielding the power supply and function generator; 4 turning on the power supply and function generator, and only shielding the photoacoustic patch. For the last situation, we assume it is the ideal case since it should eliminate the noise theoretically. We saved and analyzed the background noise at these four conditions. **a,** 500 frames of time-domain background noise at these four different situations are shown here. Only 500 frames of signals were measured to save storage space. It is obvious that strong RF noise exists in the first three cases, while shielding the patch itself can decrease the RF noise greatly. **b,** One typical frame of the temporal background noise at different situations. The background noise for the fourth situation is very stationary, only affected by weak RF noise. **c,** Summation of all of the 500 frames of noise. The summation is also used in the photoacoustic signal measurement, which is equivalent to signal averaging. The summation results show that RF noise is greatly decreased for all cases, benefiting from the data summation. All of the noise curves present the stationary feature, even without any shielding. The RF noise level may be further decreased by more average times. In addition, the noise levels of all of the first three cases are close to each other and only slightly higher than the ideal fourth case. We can also conclude that the power source and function generators introduce neglectable noise, including RF noise and stationary noise.

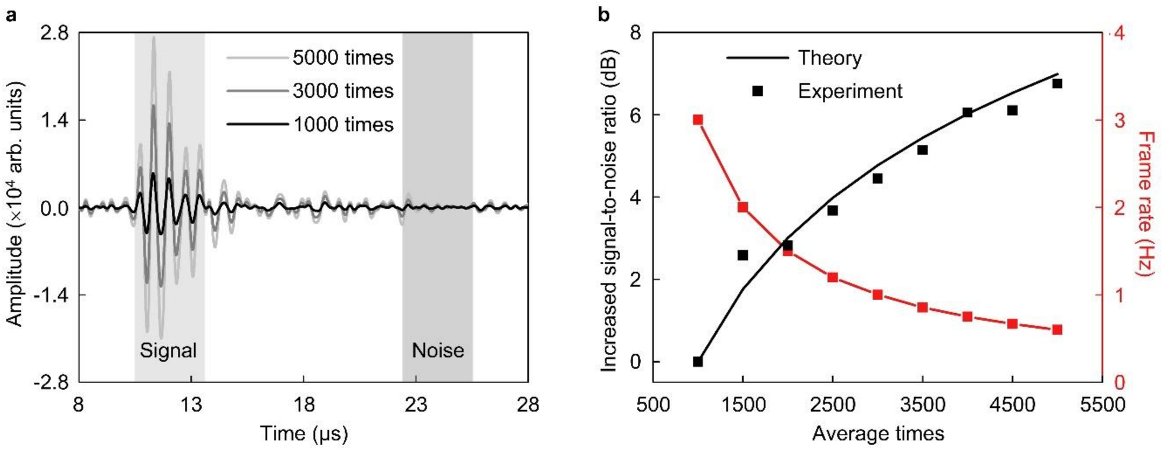

Supplementary Figure 26 | Influence of the average times on the signal-to-noise ratio. a, Photoacoustic signals averaged by 1000, 3000, and 5000 times, respectively. **b,** The signal-to-noise ratio increases as the average times. The increased value is calculated by setting the value averaged by 1000 times as the baseline. The frame rate decreases as the average times. 3000 average times are selected in this work because of the trade-off between the signal-to-noise ratio and the frame rate.

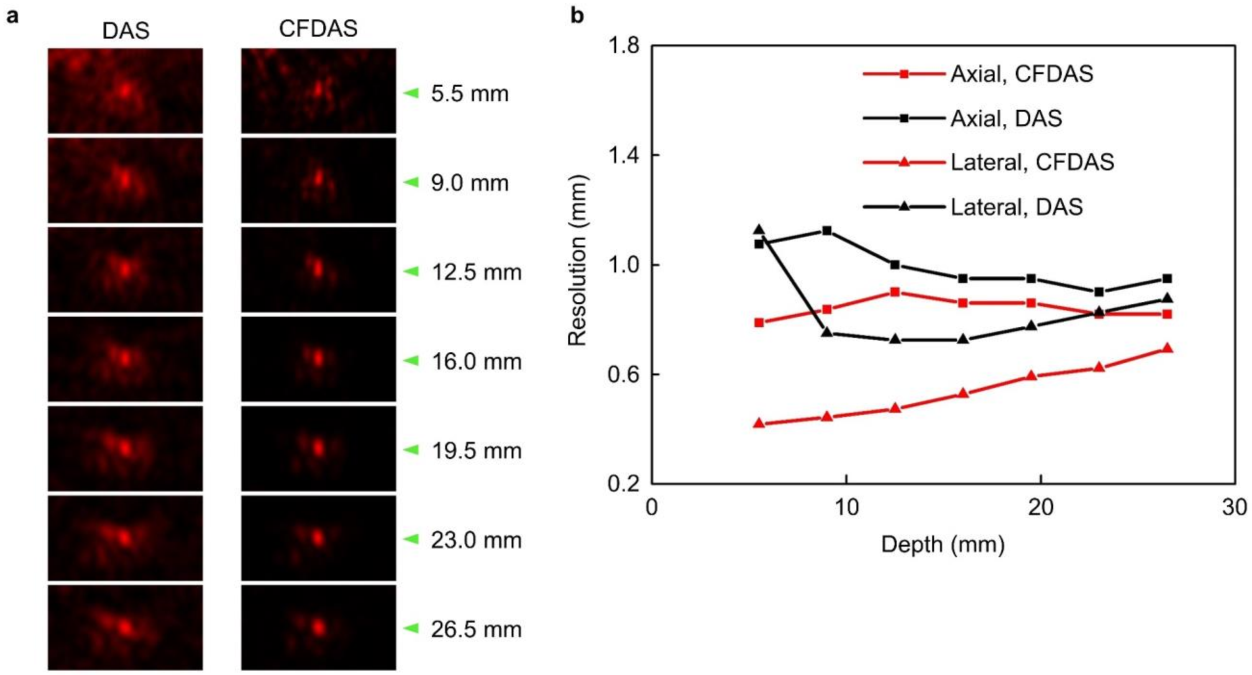

Supplementary Figure 27 | Comparison between two different image reconstruction algorithms, Delay And Sum (DAS) and Coherent Factor weighted Delay And Sum (CFDAS). **a,** Photoacoustic images are reconstructed based on the DAS and CFDAS, respectively. To characterize the resolutions of a photoacoustic imaging system, a linear object with very small diameter is usually used, such as hairs[91,111,112], carbon fibers[113,114]. We adopted those well-established standard methods for resolution characterization by embedding the linear sources in real biological tissues[112], water[113-117], or water like gelatin phantoms[91,111,118,119]. According to the literature, we also find that most studies show the resolution characterization in water or water like gelatin phantoms. Water or water like gelatin phantoms have lower optical absorption and scattering coefficients than realistic biological tissues, which will improve the signal to noise ratio of photoacoustic signals and contrast to noise ratio of photoacoustic images. But they will not affect the characterization results of imaging resolution. Therefore, we used the gelatin phantom as the background media for better accuracy and ease of operation. The hairs were embedded in different gelatin phantoms, respectively. **b,** Axial and lateral resolutions versus the depth. The CFDAS offers better axial and lateral resolutions than the DAS at all depths and is thus used in this work.

a
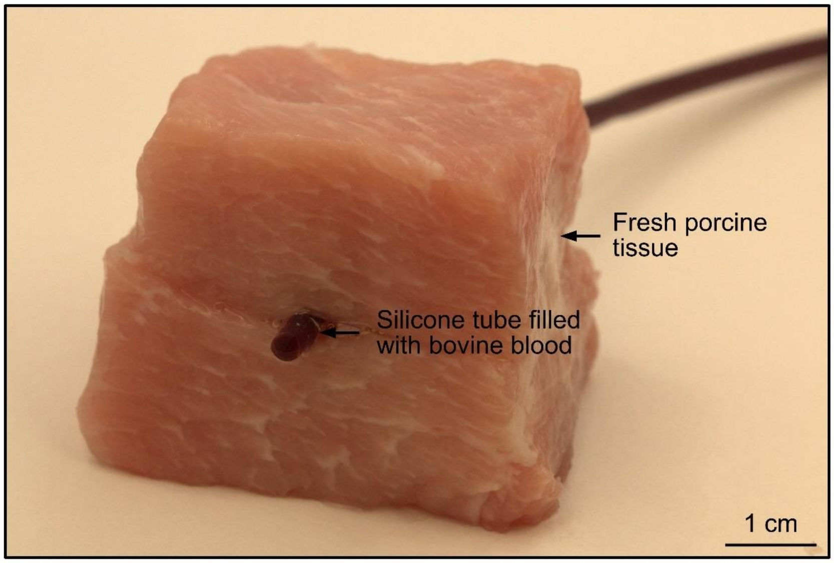

b
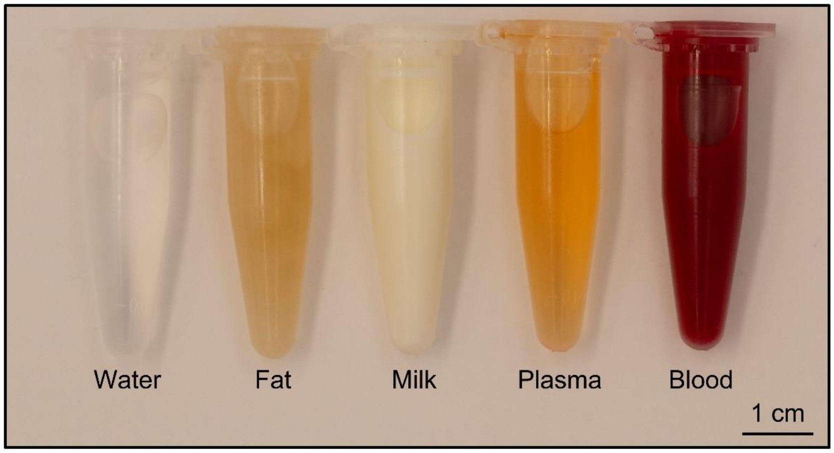

Supplementary Figure 28 | Optical photographs of the cyst phantom and different biofluids. **a,** The cyst phantom is made of porcine tissues and a transparent silicone tube filled with different biofluids. **b,** Various biofluids that can probably be found in cysts in the human body.

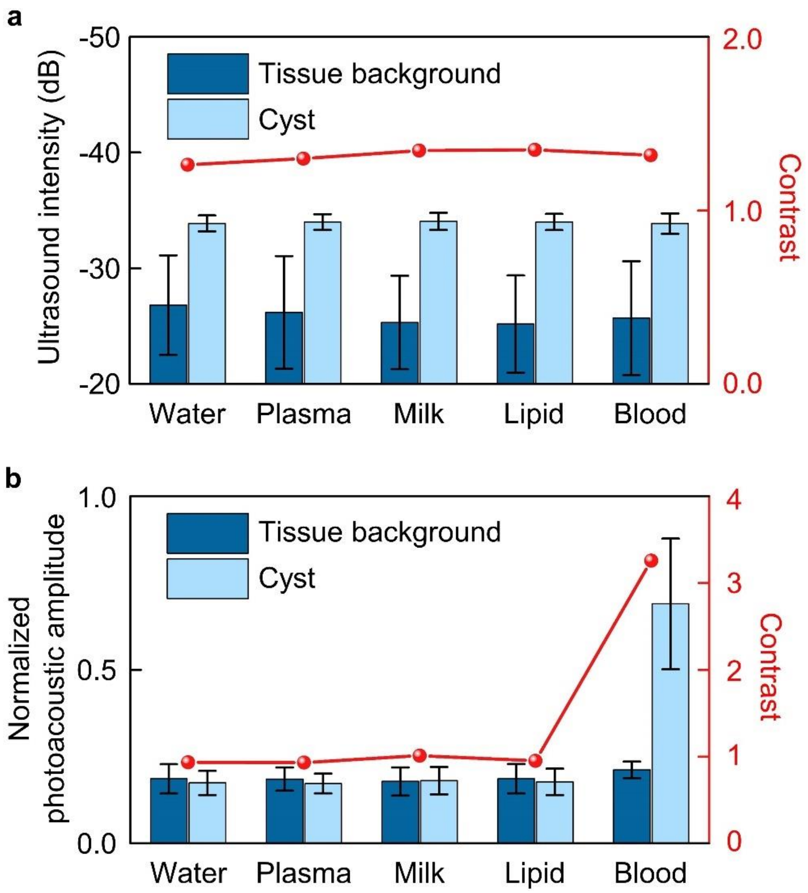

Supplementary Figure 29 | The average ultrasound intensities and photoacoustic amplitudes inside and outside the cysts. a, For the ultrasound images, the intensities of the tissue backgrounds and cyst inclusions are close to each other for all cysts, as well as their intensity contrasts. The bars show the average ultrasound intensities of 400 image pixels in the cyst and background, respectively. The error bar shows the standard deviation. **b,** For the photoacoustic images, the amplitudes of the tissue backgrounds are the same for all cysts, but the blood cyst has the highest amplitude and therefore the highest inside to outside intensity contrast among all cysts. The bars show the average photoacoustic amplitudes of 2000 image pixels in the cyst and background, respectively. The error bar shows the standard deviation.

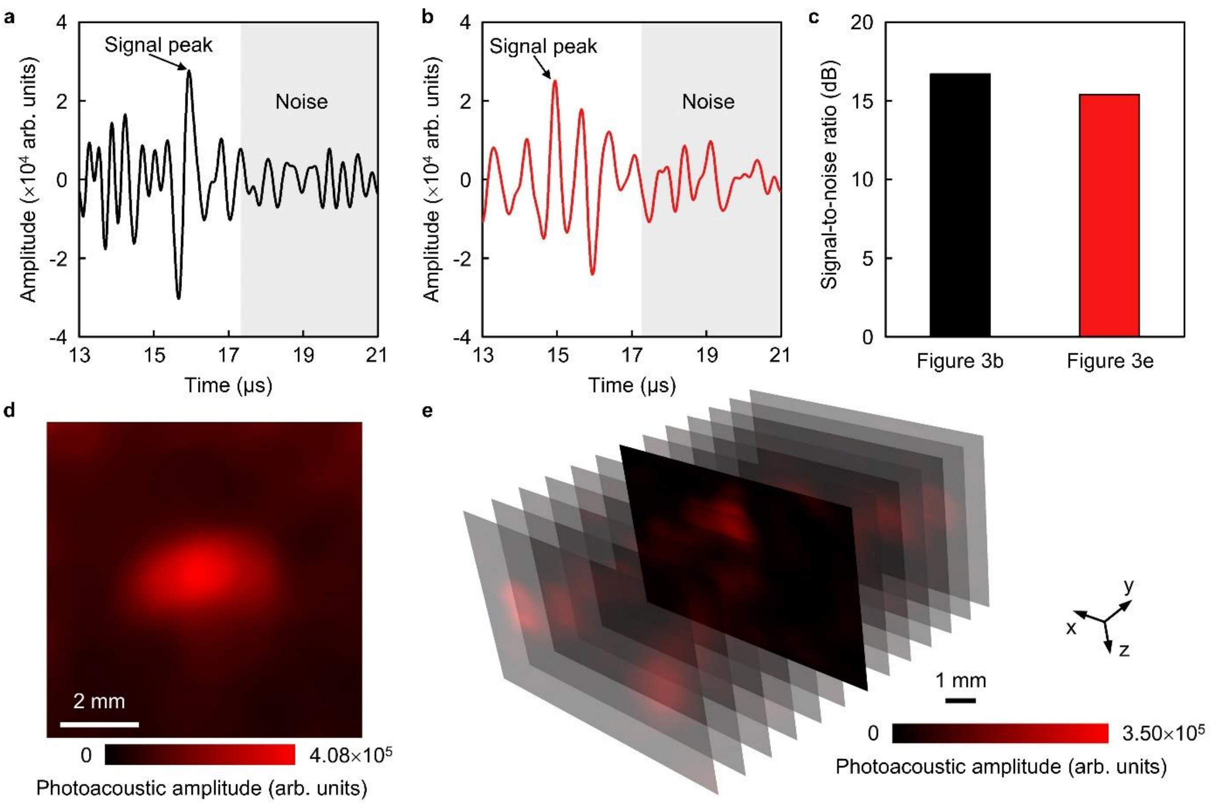

Supplementary Figure 30 | Supplementary experimental data in porcine tissues. a, A typical time-domain signal of the phantom in Fig. 3b. **b,** A typical time-domain signal of the phantom in Fig. 3e. **c,** Signal-to-noise ratios of the signals in **a** and **b**. Signal-to-noise ratio is defined as 20log10(Peak photoacoustic signal/Root mean squared error of noise)[108-110]. **d,** The image in Fig. 3b in absolute scale. **e,** The image in Fig. 3d in absolute scale.

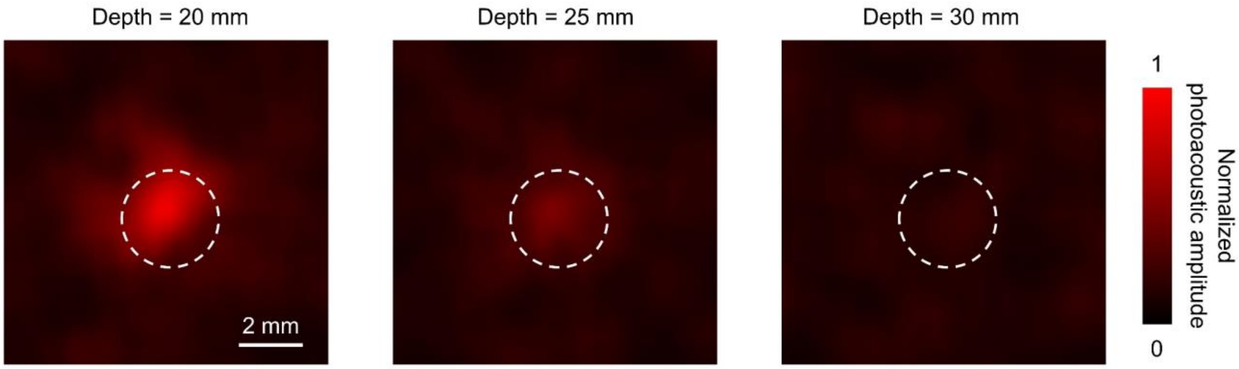

Supplementary Figure 31 | Images of the silicone tube in the porcine tissue at different depths. To determine the maximum detection depth of the photoacoustic patch, we performed an *ex-vivo* test on porcine tissue phantoms. We embedded a silicone tube under porcine tissues. The tube was filled with bovine blood. We tested the photoacoustic signals of the tube at the depth of 2 cm, 2.5 cm, and 3 cm. The photoacoustic images were reconstructed and shown. All the images were normalized by the same factor. At the depth of 20 mm, the tube has higher amplitude than the background, which shows good image result. At the depth of 25 mm, the amplitude of the tube is slightly higher than the background. At the depth of 30 mm, the tube and the background media are indistinguishable, which means the photoacoustic patch cannot operate at this detection depth. Therefore, we can determine the maximum penetration depth of the photoacoustic patch is about 25 mm on *ex-vivo* porcine tissue phantoms.

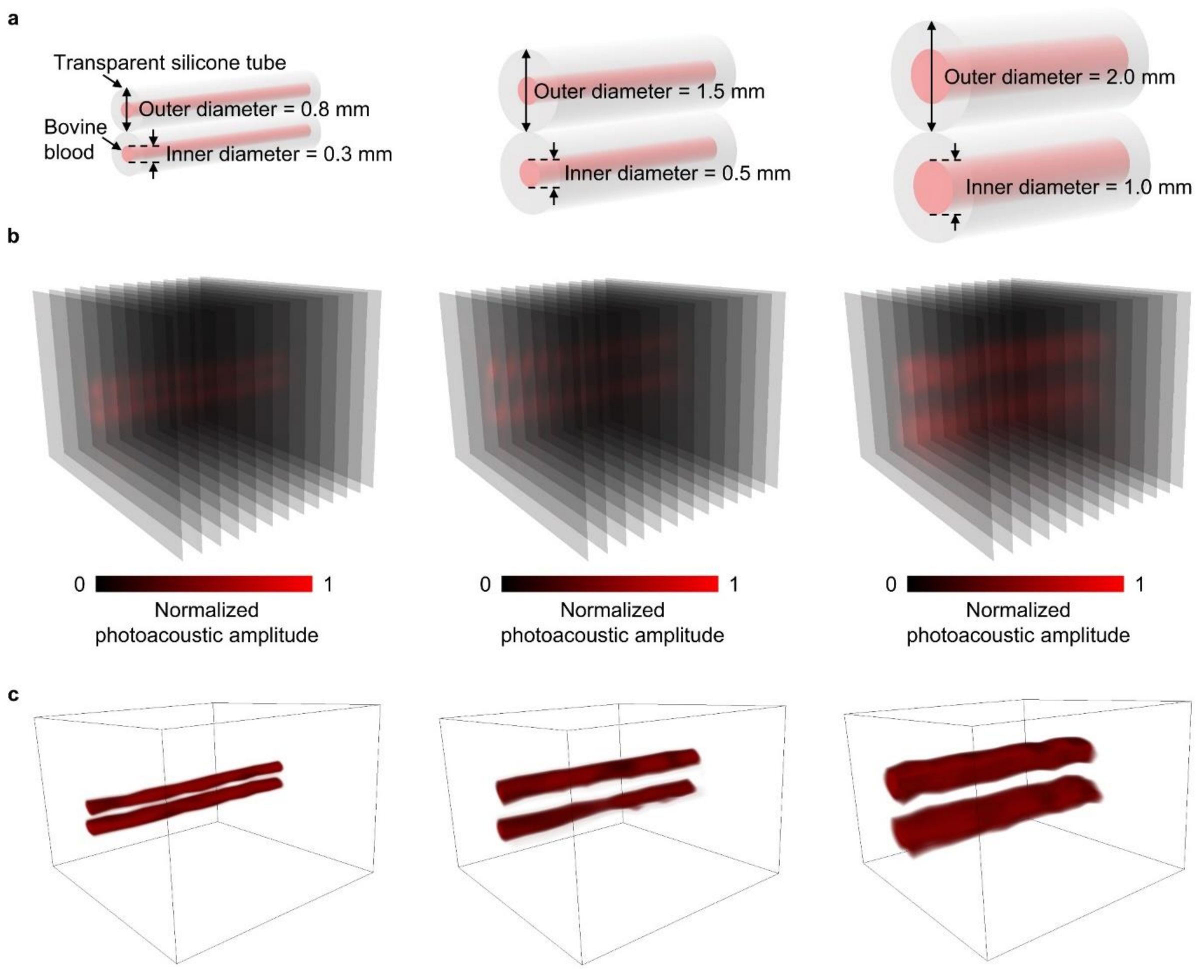

Supplementary Figure 32 | 3D photoacoustic imaging of overlapping vessels. To test the capability of 3D imaging of two overlapping vessels, we detected three different gelatin phantoms. **a**, Schematics of three phantoms, which contain two overlapping transparent silicone tubes, respectively. The tubes are filled with bovine blood. The outer diameters of the tubes are 0.8, 1.5, and 2.0 mm, while the inner diameters are 0.3, 0.5, and 1.0 mm, respectively. Those numbers resemble typical sizes of major vessels in the human body. **b**, 13 slices of reconstructed 2D photoacoustic images of the overlapping tubes. It is clear that all of the overlapping vessels are distinguishable. **c**, 3D images of the corresponding overlapping vessels.

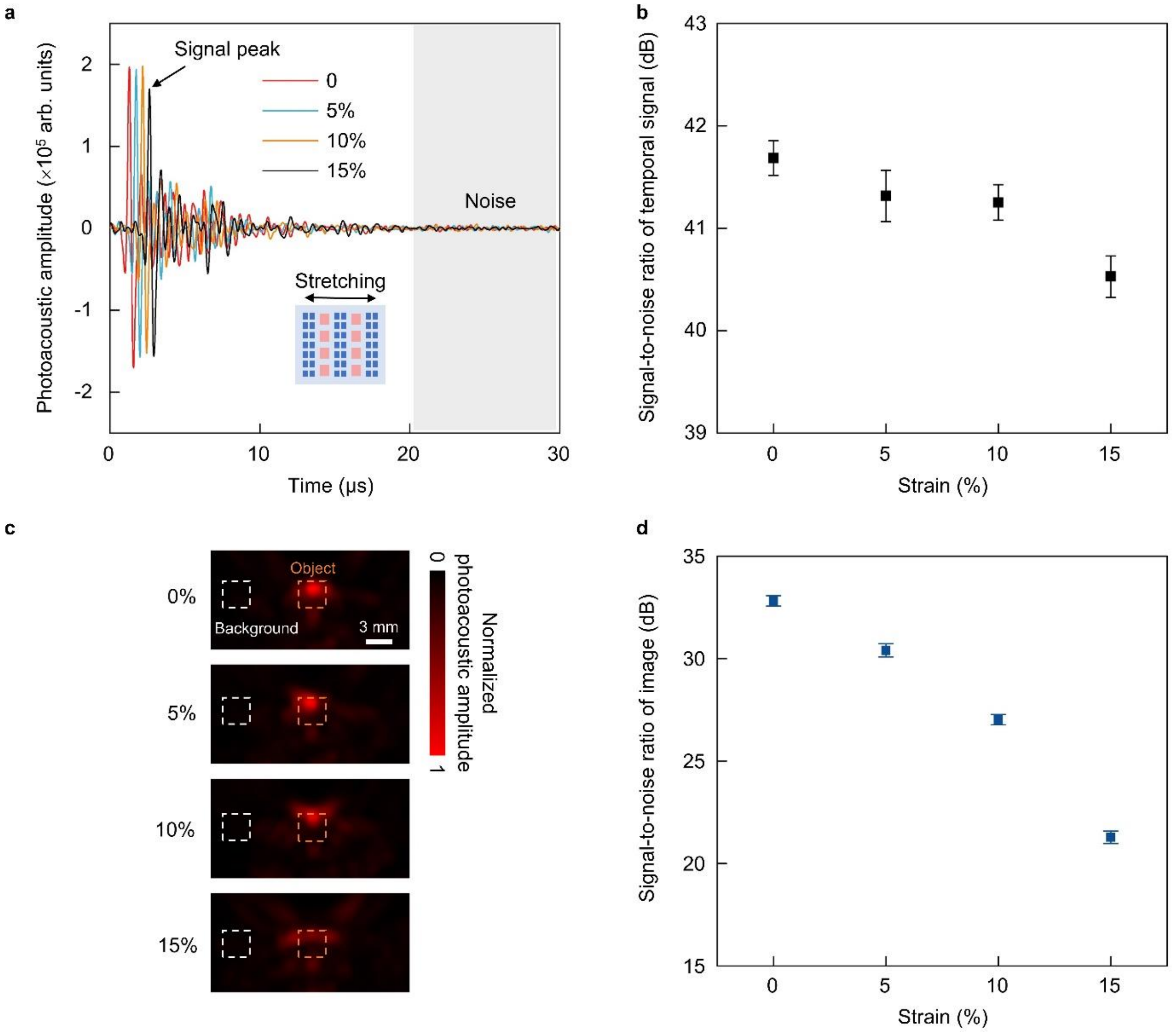

Supplementary Figure 33 | Influence of strain on the SNR of the patch. To test the influence of stretching on the device performance, we tested the device on a gelatin phantom, which contained a transparent silicone tube filled with bovine blood. We quantitatively calculated the signal to noise ratio (SNR) of both temporal signals and photoacoustic images under different strain levels. **a**, The photoacoustic patch was stretched uniaxially along the horizontal direction by 0%, 5%, 10%, and 15%. Typical temporal photoacoustic signals of one ultrasound transducer are shown when the patch was under different strains. The signals under strains are shifted in time by about 0.5, 1, and 1.5 μs, respectively to make waveforms visually distinguishable. When the patch was stretched, the distance of the laser diodes increased. This could slightly decrease the optical intensity in the phantom. Therefore, the amplitude of photoacoustic signal slightly decreased. SNR of temporal signal is defined as 20log10(Peak photoacoustic signal/Root mean squared error of noise)[108-110]. **b**, SNR of temporal signals has a small decrease as the patch is stretched. Data are presented as mean values +/- standard deviation of 10 measurements. However, stretching would also increase the distance between ultrasound transducer elements. As a result, the image reconstruction will be affected if we still use the original transducer positions in the beamforming algorithm. **c**, The photoacoustic images under different strain levels. As the strain increases, the reconstructed images show stronger distortion. The image quality substantially degraded when the

strain level reached 15%. For photoacoustic images, the SNR is defined as 20log10(Average pixel values in the region of object (orange box)/Standard deviation of pixel values in the background (white box))[86,120]. **d**, The image SNR will decrease as the strain level increases. Data are presented as mean values +/- standard deviation of 10 measurements. In this work, the photoacoustic patch could be attached to the skin with minimal stretching (<5%). The image quality does not show noticeable degradation at 5% strain. For future applications when large strain is required, we can add an additional strain sensor on the photoacoustic patch to monitor the strain level as a way to compensate for the change of distance between transducers.

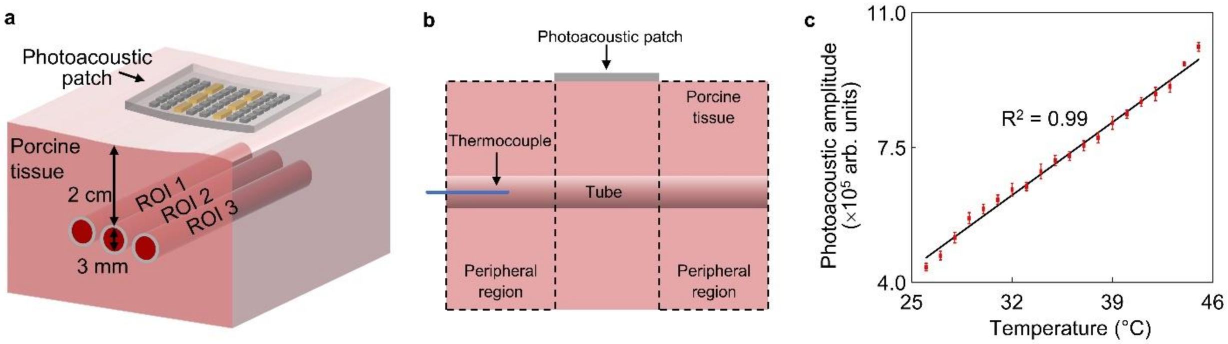

Supplementary Figure 34 | Relationship between the temperature measured by the thermocouple and the photoacoustic amplitude measured by the patch. To test the ability of photoacoustic patch to measure core temperature, we used the soft photoacoustic patch to measure the temperature in a phantom and checked its performance with thermocouples. **a,** The phantom is composed of warm bovine blood injected in three regions of interest (ROIs) underneath 2 cm thick room-temperature porcine tissues. Thermocouples were placed in the tubes, where the photoacoustic measurements were also taken for validation. **b**, Schematic showing the side view of the corresponding phantom. The peripheral regions mean those far from the photoacoustic patch in the horizontal direction, not directly underneath the patch. The porcine tissue phantom is large enough to keep the temperature uniform along the flowing direction inside the tubes enclosed by porcine tissues. **c**, Beamformed photoacoustic amplitude verse the temperature in the center of ROI 2. Data are presented as mean values +/- standard deviation of 20 measurements. Linear fitting (R^2 ~ 0.99) demonstrates the feasibility of core temperature measurement by the patch.

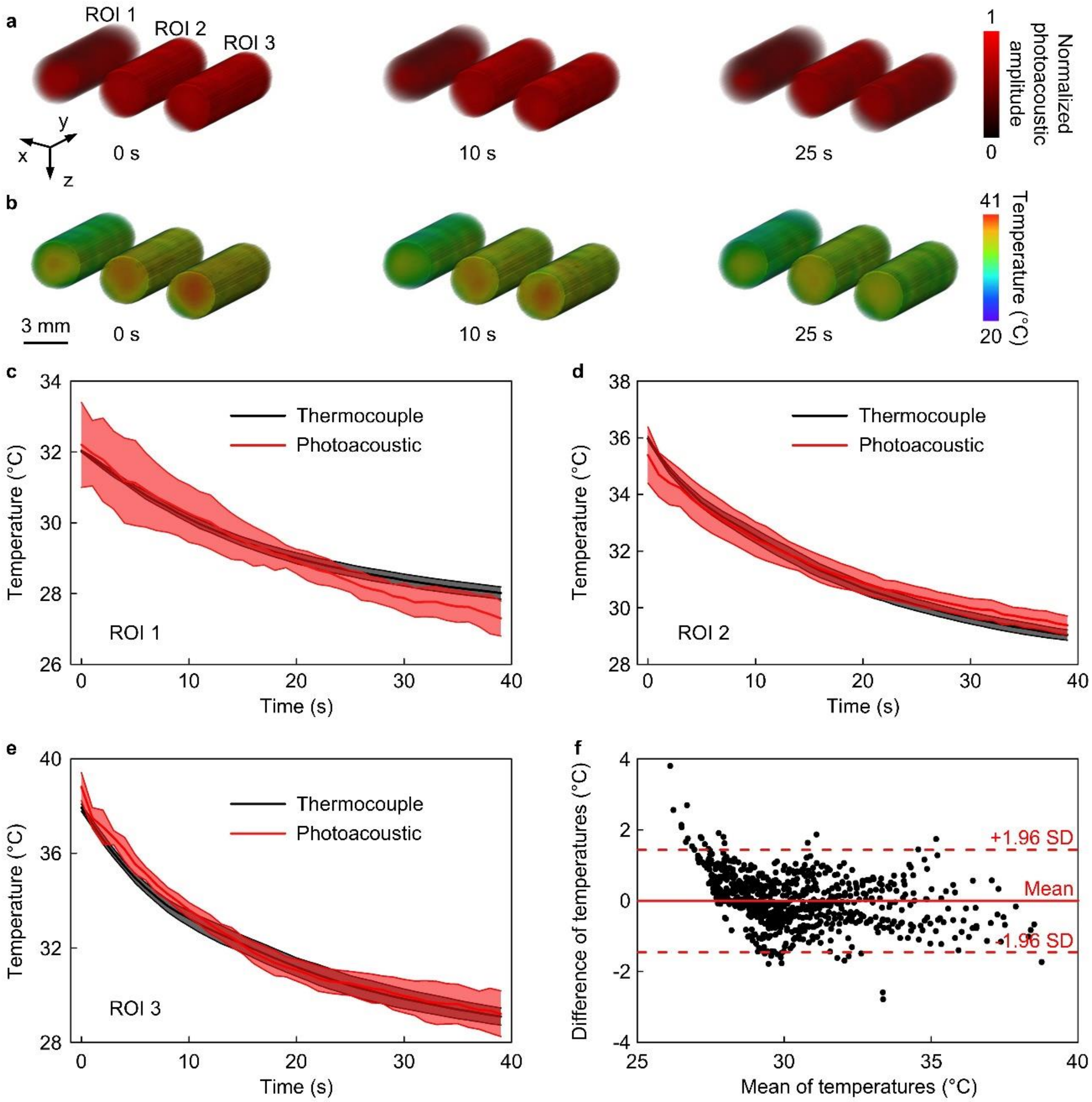

Supplementary Figure 35 | Response of the patch to dynamic temperature changes by continuously measuring warm bovine blood as they were injected into the phantom (Fig. S34)**.** Upon injection, the temperatures of the three regions of interest (ROIs) were close to 32, 36, and 38 °C, which then quickly decreased. The measurement results were validated simultaneously by thermocouples. We injected the same kind of whole bovine blood into the three tubes using three syringes, respectively. Each syringe was filled with warm blood at different temperatures. Therefore, after injecting the blood into the tubes, the initial temperatures in the tubes are different, resulting in different temperature profiles at different moments. **a,** Photoacoustic images, and **b,** Temperature mappings captured by the photoacoustic patch at 0, 10, and 25 s after injecting warm blood into the tubes. **c-e,** Changes in temperature measured by the photoacoustic patch and the thermocouples in ROIs 1-3. The black curves are the mean values of five thermocouple measurements, and the red curves are the mean values of five photoacoustic measurements. The shades of the curves are standard deviations of the measurements. **f,** Bland-Altman plot (Supplementary Note 7) showing the statistical analysis of 600 pairs of results measured by the thermocouple and photoacoustic patch. The horizontal axis is the mean of the temperatures

measured by the two devices, while the vertical axis is the difference between them. Bland-Altman plot analyzes the agreement between two datasets measured by two detection methods[64], which is widely used to compare a new measurement technique with the gold standard[65-67]. Mean defines the average value of difference. −1.96 SD (i.e., standard deviation) and +1.96 SD label the lower and upper limit boundaries of the 95-confidence interval in standard normal distribution. As shown, most (94.8%) of the datapoints are within ±1.96 standard deviations difference, demonstrating the excellent agreement between the two devices with a high statistical robustness. According to our calculation, the standard deviation between the thermocouple and photoacoustic patch is about 0.7 °C, which is considered as the accuracy of the photoacoustic patch at the depth of 2 cm in *ex-vivo* porcine tissue. The high accuracy of temperature measurement benefits from the high power of laser diodes and high receiving sensitivity of ultrasound transducers. The entire patch has a pulse energy of about 0.192 mJ with a pulse duration of 200 ns. This power is close to the 0.2 mJ of a commercial LED-array-based photoacoustic imaging system[38,91], which has achieved *in-vivo* imaging at a depth of over 2 cm. Comparison of measured receiving sensitivity between the photoacoustic patch and commercial ultrasound probe also shows they have similar performance on the wave receiving (Fig. S22). There are many studies in the literature that have reported accuracies better than this work, such as 0.6 °C in a deep chicken[121], 0.2 °C and 0.5 °C on phantom and animal using a portable photoacoustic system[122], and a high accuracy of 0.18 °C in a photoacoustic based close-loop temperature control system[123]. A temperature accuracy of 0.16 °C on porcine tissues has also been reported[60]. Because this is the demonstration of proof-of-concept of flexible and stretchable photoacoustic patch, there is still a lot of space to improve, such as enhancing the laser energy.

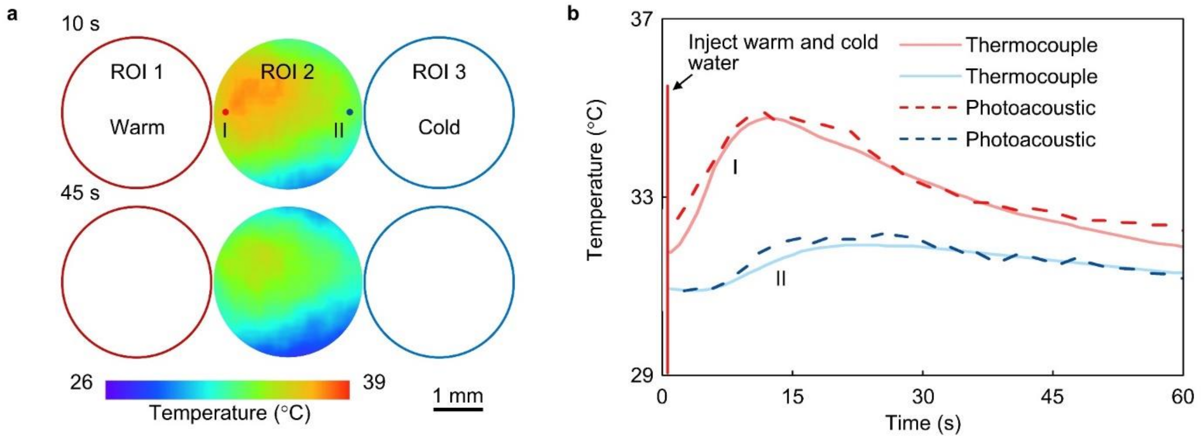

Supplementary Figure 36 | Core temperature mapping using the photoacoustic patch. We tested regions of interest (ROI) 2 (Fig. S34), filled with room temperature blood, under a changing thermal gradient created by all of the ROIs. We first injected blood at the room temperature into ROI 2. The blood in ROI 2 was static during the experiment. Then, we quickly injected warm and cold water into ROIs 1 and 3, respectively. The water flow in ROIs 1 and 3 stopped after the warm and cold water filled the tubes fully, which was achieved within 1 second. After the injection, fluids in ROIs 1, 2, and 3 all kept static. We used the photoacoustic patch to image the temperature gradient in ROI 2 created by all the ROIs. **a**, The mapping results show that the region close to ROI 1 has a much higher temperature than that near ROI 3 initially (top panel); the difference decreases rapidly with time (bottom panel). To verify the accuracy of these results, two thermocouples are placed in the ROI 2, labeled as points I and II. **b**, The temporal temperature curves measured at these two points show a strong correlation between the thermocouples and the photoacoustic patch.

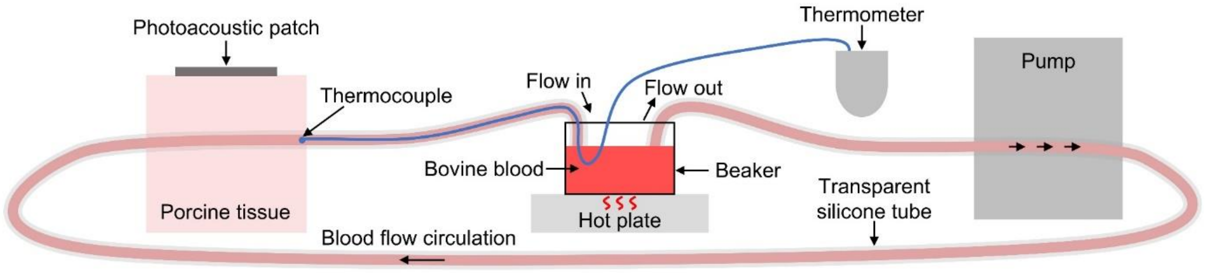

Supplementary Figure 37 | Schematic showing the experimental setup. We used a pump (Huiyu, BT300J-1A) to drive the blood to flow in a transparent silicone tube with an inner diameter of 3 mm. The rate of flow was set to be ~9 mL s^{-1}, resulting in a blood flowing speed of ~127 cm s^{-1}, which was higher than the blood flow velocity of most blood vessels in the human body. The two ends of the tube were immersed in a breaker containing bovine blood. The beaker was placed on a hot plate to heat the blood to different temperatures during flowing. The blood could also naturally cool down while the hot plate was turned off. A portion of the tube was embedded underneath a porcine tissue at a depth of ~2 cm, which was measured by the photoacoustic patch. A soft thermocouple was inserted into the tube to measure the blood temperature simultaneously. The data was read by a thermometer and recorded in a laptop continuously.

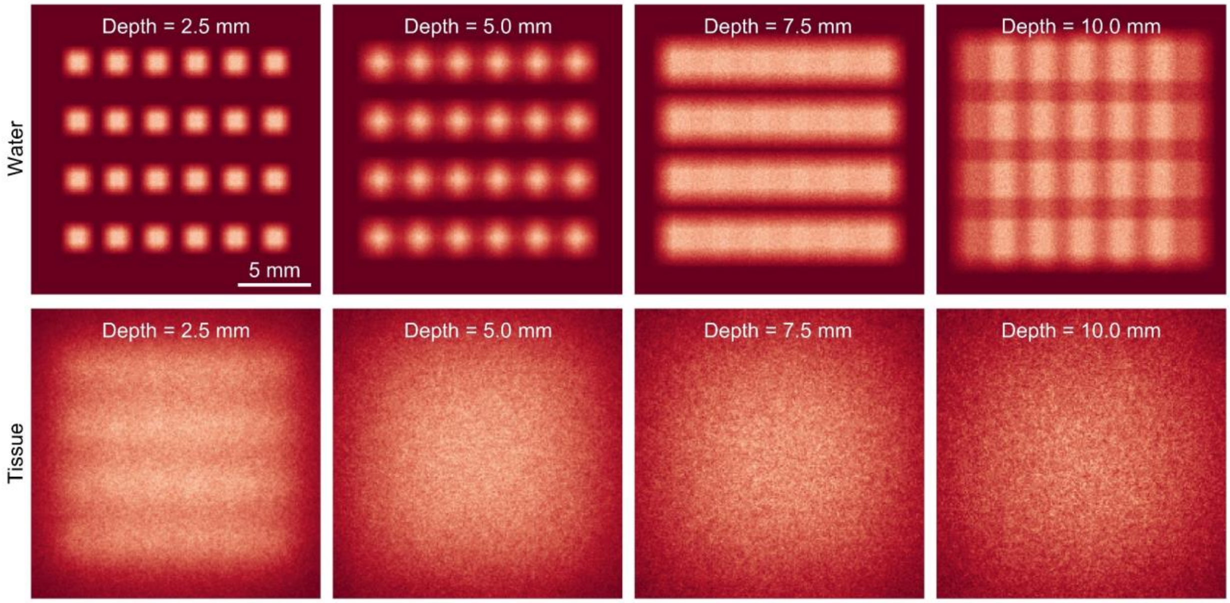

Supplementary Figure 38 | Optical distribution simulation at different depths in the water and tissue performed in an open-source MATLAB toolbox — MCmatlab. For conventional photoacoustic imaging systems, the transducer array is usually immersed in water[9,10], which serves as the acoustic coupling media between the array and tissue. Ultrasound gel can be also applied as an alternative acoustic coupling media. Water and ultrasound gel both have high optical transparency and low scattering coefficients, which means they have low diffusion capability. In this case, if the optical beam illuminated from an optical fiber or a prism is not uniform, a long stand-off distance (about 10 mm) is usually required to make sure the optical beam reach the tissue skin as uniform as possible. To decrease this stand-off distance or expand the illumination area, an optical diffuser[9,10] can be inserted between the optical source and the tissue. The results in the figure are all normalized individually to show the beam pattern. When the photoacoustic patch illuminates water, the first row presents the distribution of optical intensity at different depths. The absorption coefficient μ_a, scattering coefficient μ_s, Henyey–Greenstein scattering anisotropy factor g, and refractive index n are set as 0.00036 cm^{-1}, 10 cm^{-1}, 1, and 1.3, respectively. It is obvious that the optical beam is not uniform. This explains why conventional photoacoustic imaging systems need a long stand-off to achieve a uniform beam pattern on the skin surface. The second row shows the optical distribution at different depths in the tissue. The absorption coefficient μ_a, scattering coefficient μ_s, Henyey–Greenstein scattering anisotropy factor g, and refractive index n are set as 0.1 cm^{-1}, 85 cm^{-1}, 0.9, and 1.3, respectively. The results show that biological tissue has strong diffusion effect on the laser beam. There is a stand-off distance for our patch, which is less than 2.5 mm. The distribution of optical pattern is even more uniform at the depth of 2.5 mm in the tissue than that at the depth of 10 mm in the water. In our studies, we are interested in deep tissues (>5 mm), beyond which the optical distribution is very uniform. Except for the imaging of superficial veins in the hand, foot, thigh, and forearm, all the other experiments are not affected by the stand-off distance. When detecting the superficial veins, a 1 cm-thick gelatin phantom was added between the patch and forearm to compensate for this stand-off distance.

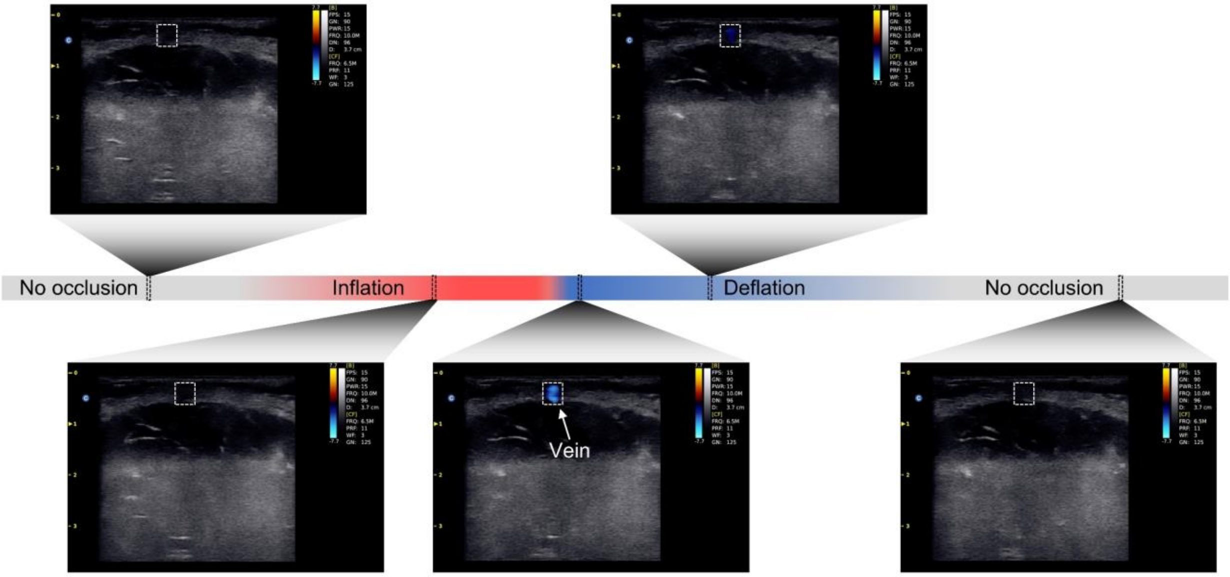

Supplementary Figure 39 | Ultrasound B-mode and color flow Doppler images of veins in the forearm acquired by a commercial ultrasound system (Chison ECO5) during venous occlusion test. The working frequency was 10 MHz. The dual mode images — ultrasound B-mode and color Doppler image, at different stages of venous occlusion test are shown in this figure. Due to the low sensitivity of ultrasound Doppler to slow blood flow, we used a cuff to induce ischemia followed by sudden release of the cuff to increase the blood flow in the vein. Five images were measured before occlusion, during inflation (~ 90 mmHg), right after deflation, after deflation, and no occlusion. Blood flow is undetectable in the first, second, and fifth images. The third image shows the moment immediately after the cuff was released. At that moment, the blood flow was the fastest and thus detected. And the blood flow quickly vanished in the image because the blood velocity dropped fast. The ultrasound Doppler detection of veins in the forearm shows low sensitivity because of the slow blood flow, which is very common for small blood vessels[124]. To the contrary, the photoacoustic patch achieves high-contrast photoacoustic images of the veins.

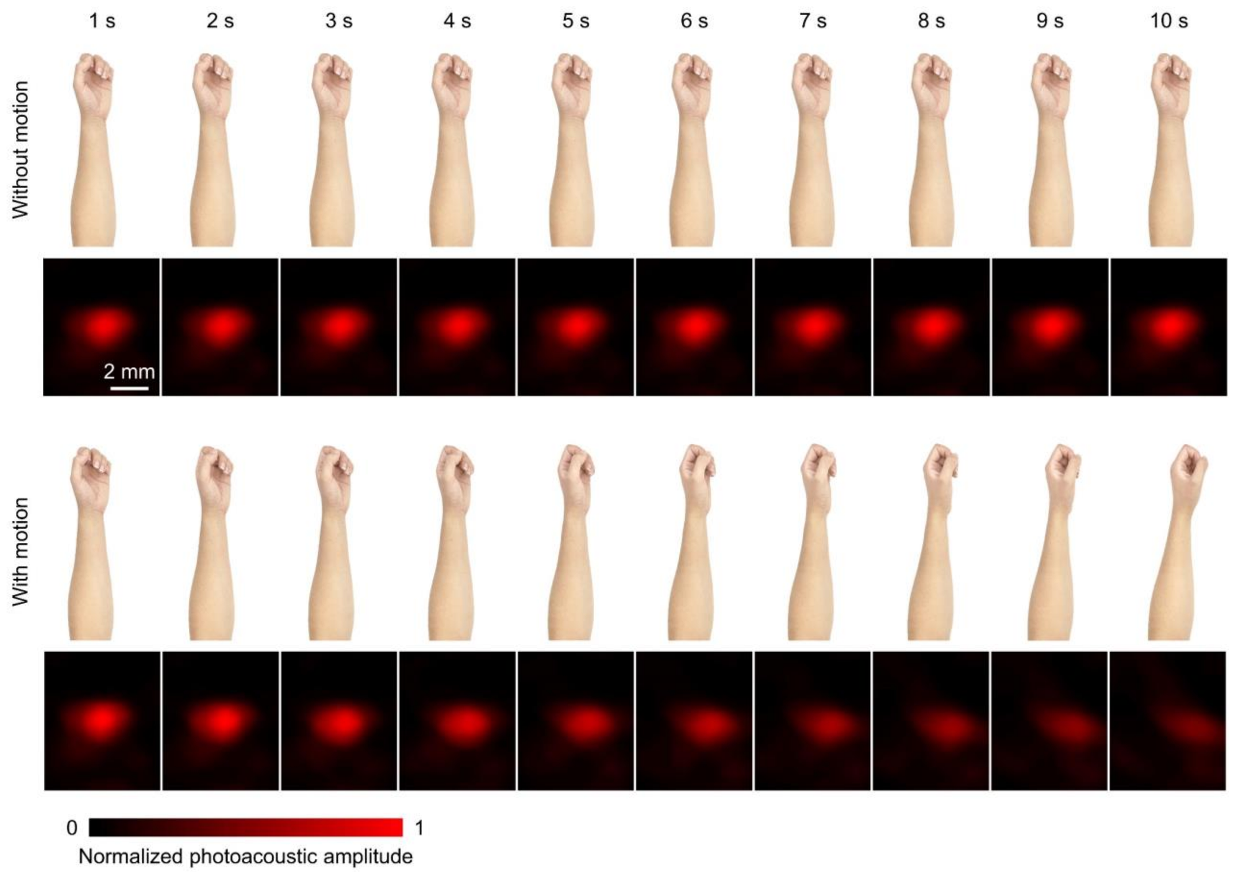

Supplementary Figure 40 | Photoacoustic imaging artifact caused by forearm movement. The figure shows two measurements of the vein in the forearm, which includes 10 frames of images of the vein corresponding to static forearm and moving forearm. For the situation without motion, the images of the blood vessel are stable. However, as displayed in the second row, rotating the forearm caused the displacement and distortion of the blood vessel. During the measurements of veins in this study, the volunteer kept the arm and neck still to decrease the influence of motion.

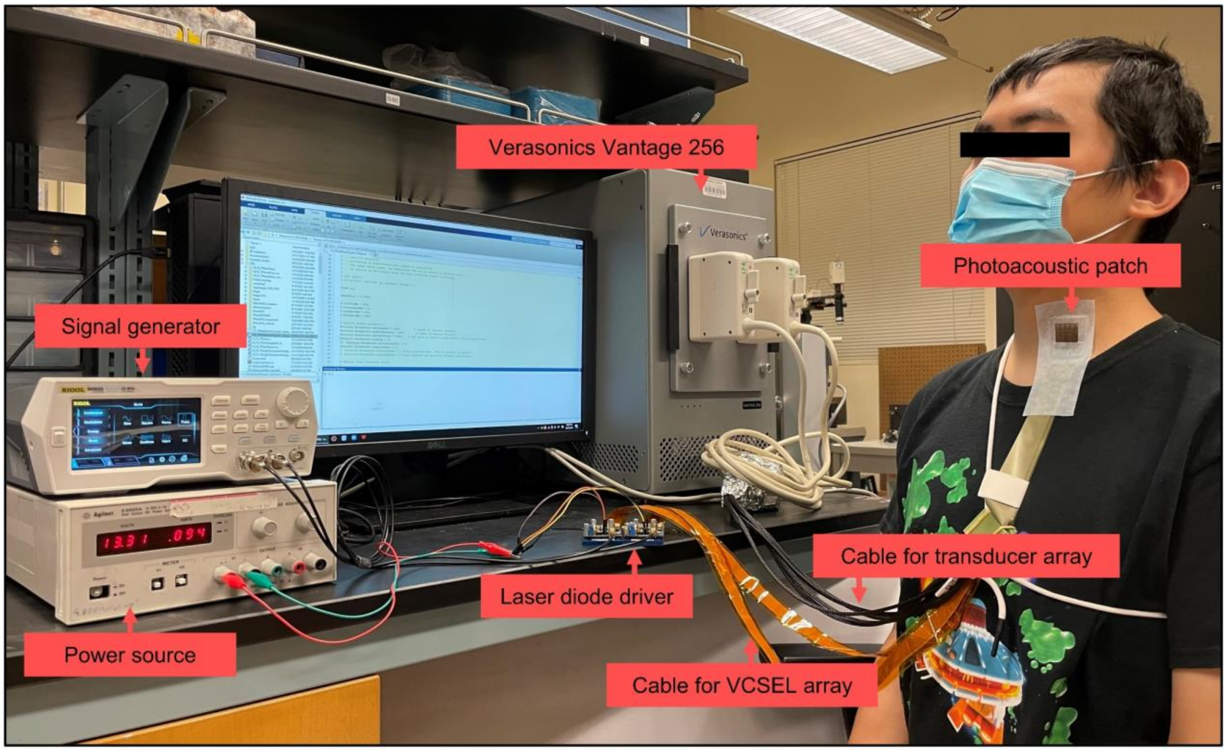

Supplementary Figure 41 | Photos of the measurement system, including the photoacoustic patch, driving circuits of the VCSEL array, and the Verasonics system. The key components have been labelled.

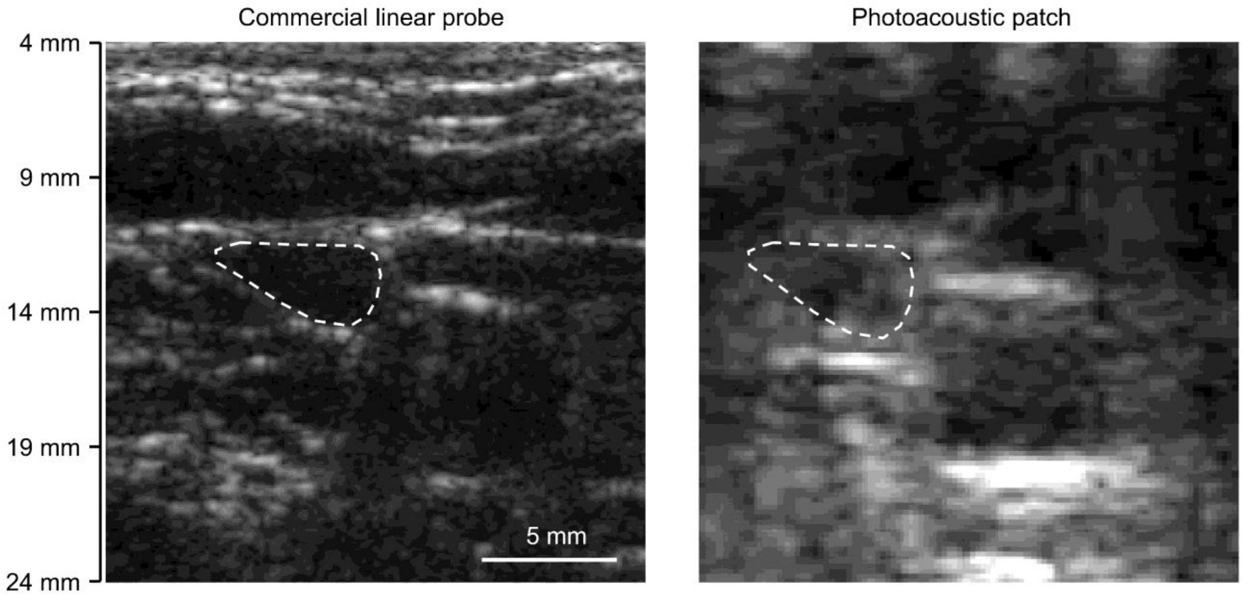

Supplementary Figure 42 | Comparison of ultrasound B-mode images acquired by a commercial probe and the photoacoustic patch. The commercial probe (Verasonics, L11-5v) has better ultrasound imaging quality than the photoacoustic patch because of the higher frequency (8 MHz) and more transducer elements (128 elements).

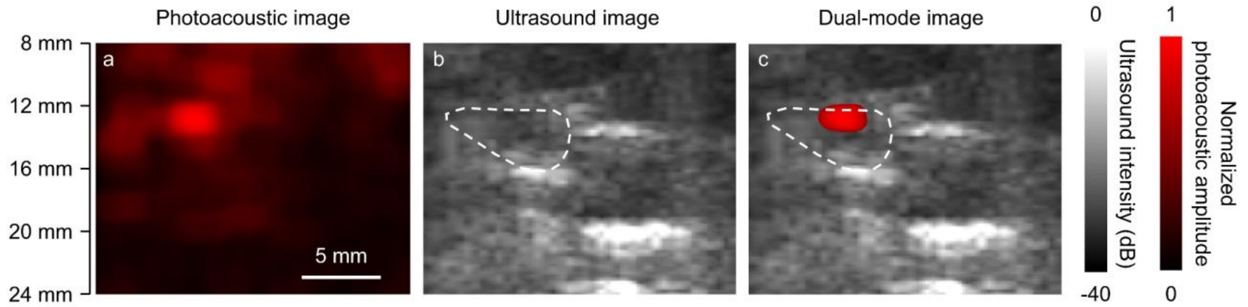

Supplementary Figure 43 | Dual-mode images of the internal jugular vein acquired by photoacoustic patch. **a**, One typical 2D photoacoustic image of the internal jugular vein. **b**, Corresponding ultrasound B-mode image. **c**, Co-registered ultrasound image and photoacoustic image, where only the photoacoustic amplitude larger than 0.5 was displayed. Even though there are some areas introduce photoacoustic contrasts, the internal jugular vein has the highest signal amplitude.

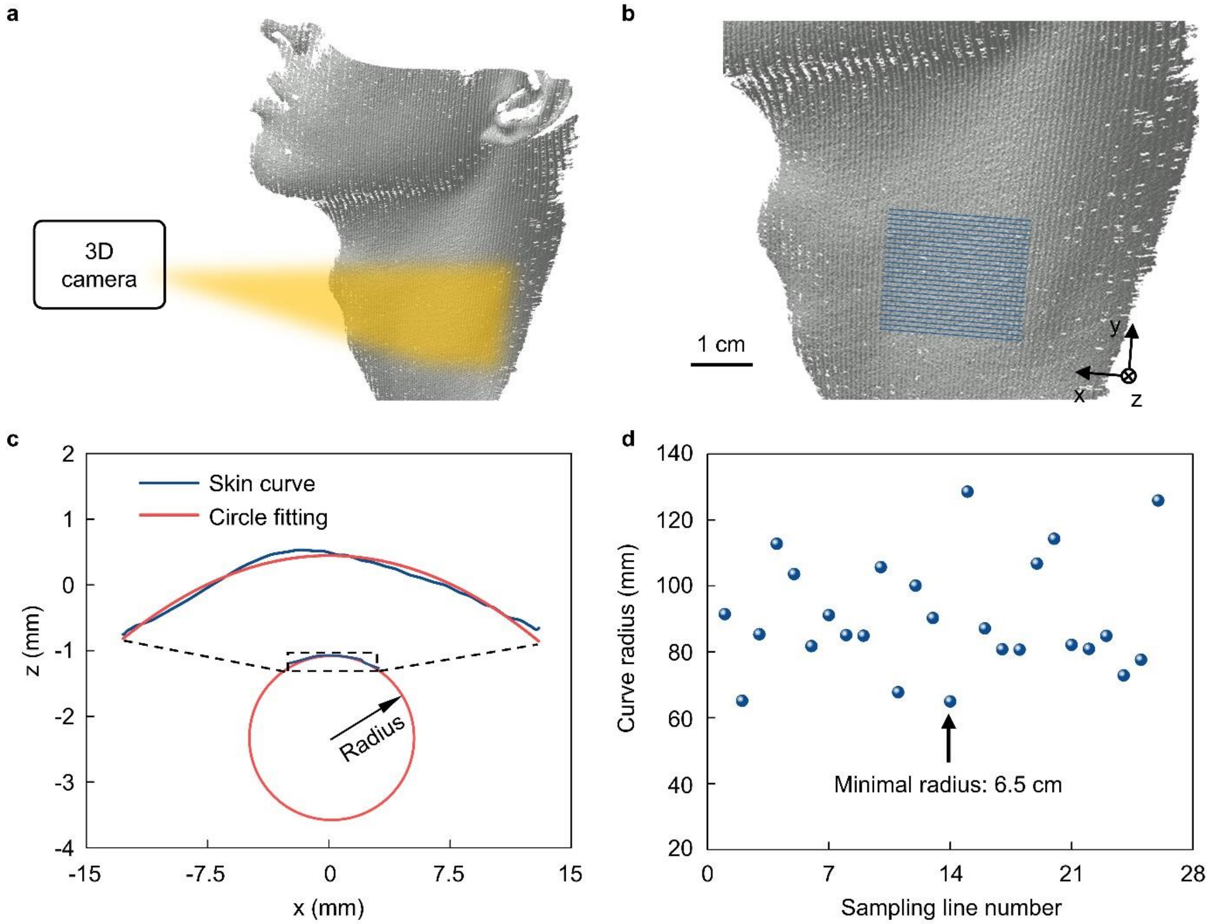

Supplementary Figure 44 | Characterization of skin curvatures by a 3D camera. a, Schematics of scanning the neck with a 3D camera. **b,** Extracting curves of the scanned skin surface. The 26 intersection lines are labeled in blue. **c,** Determining radius of skin curvature by circle fitting. **d,** Measured curvature radii at 26 positions on the subject. The smallest radius is found to be 6.5 cm, which is used in this work to evaluate the influence of the skin curvature on the imaging performance of the soft photoacoustic patch.

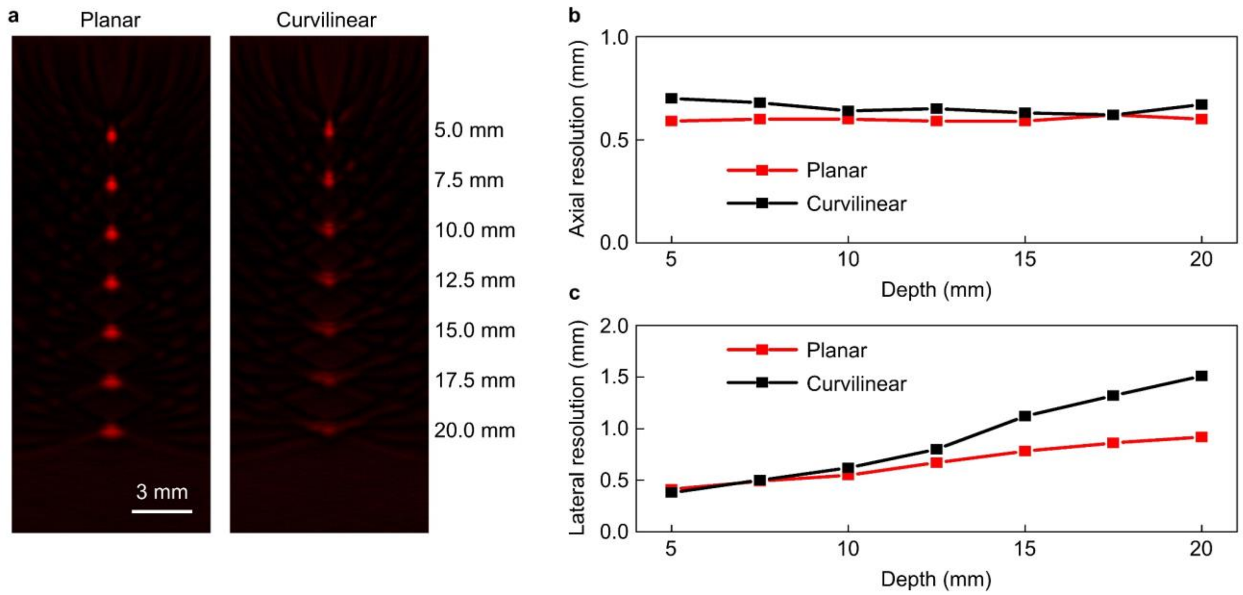

Supplementary Figure 45 | Influence of the skin curvature on the imaging results. a, Imaging results of point sources at different depths when the soft photoacoustic patch is placed on planar (left) and curvilinear (right) surfaces. Changes in **b,** the axial and **c**, the lateral resolutions at different depths when the patch is placed on the planar and curvilinear surfaces, respectively.

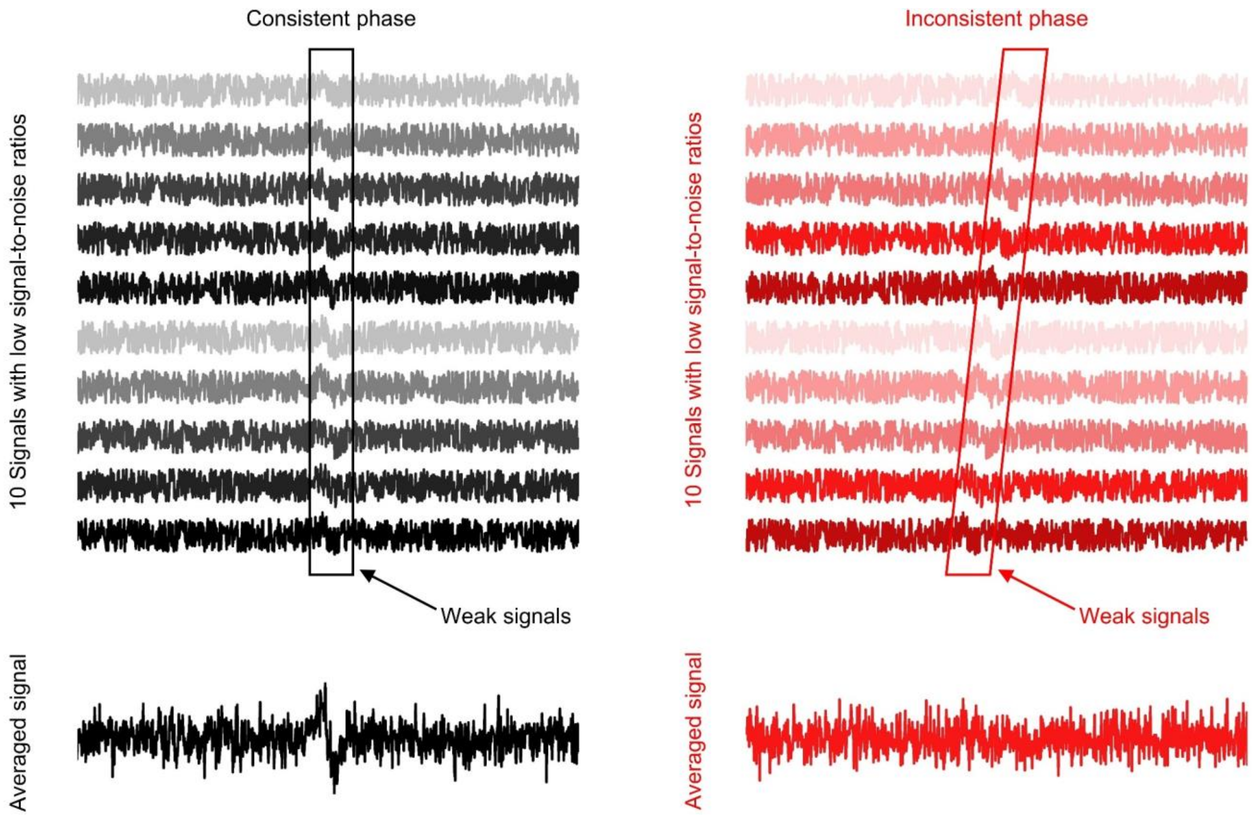

Supplementary Figure 46 | Comparison of averaged signals between consistent and inconsistent phases. A relatively static tissue yields a consistent phase in the photoacoustic signals, which can get a high signal-to-noise ratio after signal averaging. A dynamic tissue will generate inconsistent phases, resulting in a reduced signal-to-noise ratio after signal averaging.

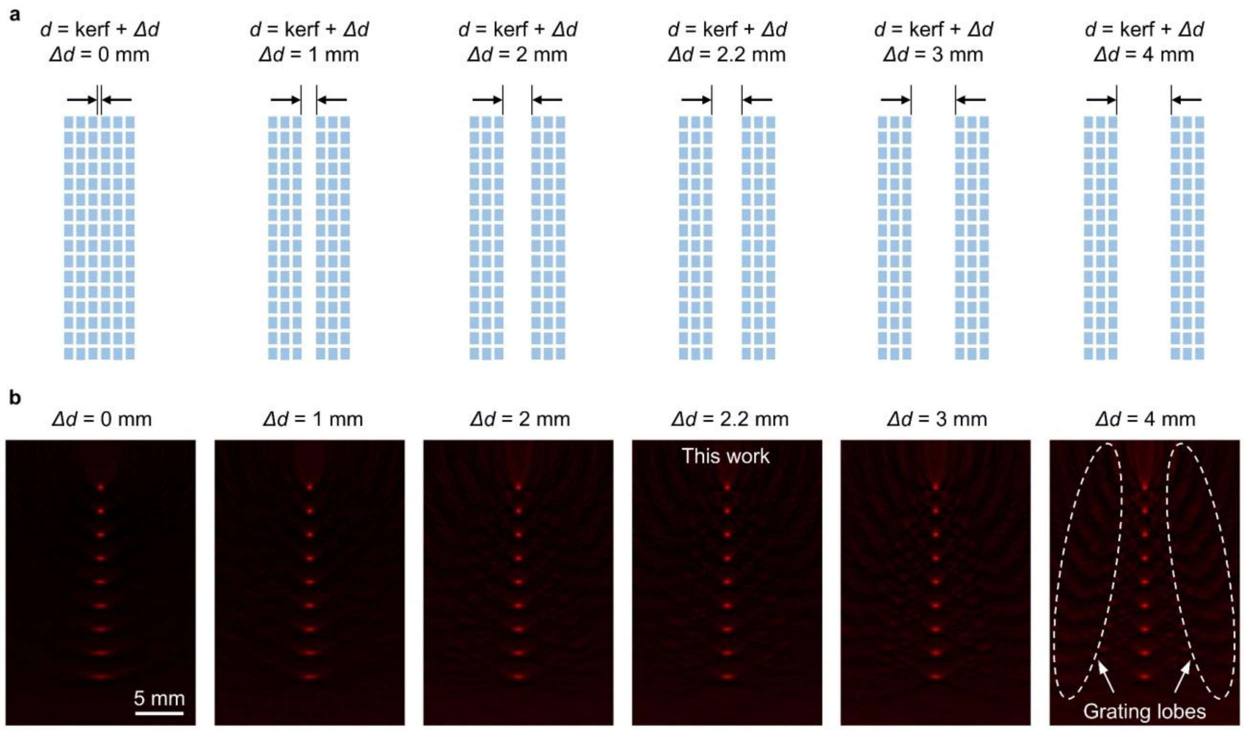

Supplementary Figure 47 | Influence of the VCSEL chip size on the imaging performance. Photoacoustic imaging simulation is performed in an open-source MATLAB toolbox — *k*-Wave. **a**, Schematics showing larger VCSEL chips lead to increased distances between the transducers. *Δd* is defined as the extra distance caused by VCSEL chips between every three columns of ultrasound transducers. **b**, Simulated reconstructed images with stronger intensities of grating lobes at increased distances between the transducers. For case *Δd* = 0 mm, the distance of each two ultrasound transducers (i.e., the pitch) is 0.8 mm, close to the wavelength at 2 MHz. The photoacoustic image shows weak grating lobes. For the other five cases, the effective pitch increases, which enhances grating lobes. However, the amplitudes of grating lobes are still much weaker than the main lobes. Furthermore, the lateral resolution is improved as the effective pitch dimension increases because the aperture of the array is increased. This Figure is correlated to Supplementary Figure 4, emphasizing the influence of VCSEL chip size.

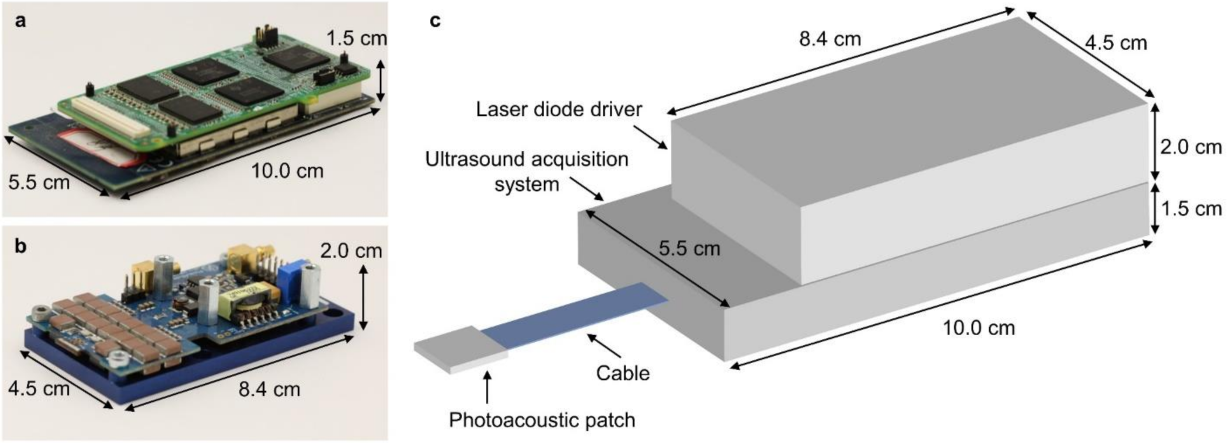

Supplementary Figure 48 | Portable photoacoustic device. The main reason why the current testing system is bulky is that we used a bulky ultrasound research platform (Verasonics system) to acquire photoacoustic signals. Verasonics system also consumes the most power in the entire device. This system is powerful but has many redundant functions that are not necessary for our applications, such as ultrasound wave transmission, high intensity focused ultrasound. The current photoacoustic patch is connected with a high-power laser diode driver and data acquisition system through some cables. Because one coaxial cable for ultrasound signal receiving only has 60 channels, we had to use multiple coaxial cables to connect all of the 240 transducer elements. Alternatively, we can use one coaxial cable containing 256 channels to reduce the number of cables. Therefore, we can keep only two cables connected with the backend controlling system, one for laser diode driving and the other for data acquisition. It is highly possible to replace the current bulky backend system with a handheld backend controlling system. The number of cables will be greatly reduced. The photoacoustic patch, including the laser diodes and ultrasound transducers, can be connected with the portable device with one customized cable. In recent years, portable ultrasound systems with ultra-compact sizes have been developed for point-of-care uses. **a,** A typical portable system developed by Texas Instruments[125], which has a size of ~10.0 cm × 5.5 cm × 1.5 cm. This system can replace the bulky Verasonics system to record photoacoustic signals. In addition, this system has low power consumption of ~2.5 W[125], which can be powered by a USB cable plugged into a laptop. **b**, The laser diode driver used in this work has a compact size of ~8.4 cm × 4.5 cm × 2.0 cm. The power consumption of the driver is less than 10 W when driving the laser diodes at a pulse repetition rate of 3 kHz. Furthermore, the power consumption is tunable and can be further cut down by reducing the laser pulse width, pulse repetition rate, and imaging frame rate. For example, assuming decreasing the imaging frame rate from 1 Hz to 0.5 Hz, the laser diode driver power consumption can be theoretically reduced by ~50%, i.e., <5 W of total power consumption. **c**, A schematic of handheld photoacoustic device with possible dimensions that are predicted based on existing electrical circuits. The power consumption of such a system can be reduced to be in the range of several Watts.

	**Wearable**	**Long-term continuous**	**Noninvasive**	**Detection depth**	**Spatial resolution**	**Citations**
**Magnetic Resonance Imaging (MRI)**	No	No	Yes	>10 cm	~1.5 mm	[1]
**Positron Emission Tomography (PET)**	No	No	Yes	>10 cm	~3 mm	[3]
**Fluorescence imaging**	No	No	Yes	~3 mm	~4 µm	[5]
**Optical Coherence Tomography (OCT)**	No	No	Yes	<2 mm	~1–10 µm	[7]
**Bulky photoacoustic imaging system**	No	No	Yes	>4 cm	<0.25 mm	[9,10]
**Electrochemical soft electronics**	Yes	Yes	Yes	~3 mm	N/A	[11]
**Optical soft electronics**	Yes	Yes	Yes	<3 mm	N/A	[12]
**Photoacoustic patch**	Yes	Yes	Yes	>2 cm	~0.7 mm	This work

Supplementary Table 1 | Comparison between different methods for biomolecular detection. In comparison to existing wearable electronics, the photoacoustic patch in this work realizes non-invasive 3D mapping of biomolecules in deep tissues. This technology not only achieves imaging resolutions and detection depth comparable with the conventional bulky systems, but also has compact size and conformal mechanical properties, which are suitable for long-term monitoring.

Type	Pulse energy	Pulse repetition rate	Imaging frame rate	Number of data averaging	Size of ultrasound probe	Size of laser source	Citation
Laser	~20 mJ cm^{-2} @ 680-950 nm	100 Hz	100 Hz (3D)	0	Spherical matrix array, cylindrical shape Diameter ~ 70 mm, Thickness > 80 mm	Not described.	84
Laser	23 mJ cm^{-2} @ 1064 nm<br>19 mJ cm^{-2} @ 694 nm	10 Hz<br>1 Hz	0.5 Hz (3D)	0	Hemispherical matrix array Diameter = 260 mm	Not described.	85
Laser	15 mJ cm^{-2} @ 532 nm, 27 mJ cm^{-2} @ 1064 nm	10 Hz	10 Hz (2D) ~0.025 Hz (3D)	0	ATL L7-4, commercial linear probe	Optical head: 775 × 178 × 190 mm Power supply: 622 × 282 × 508 mm	86
Laser	20 mJ cm^{-2} @ 730-900 nm<br>50 mJ cm^{-2} @ 1064 nm	10 Hz	10 Hz (3D)	0	Spherical matrix array with a radius of 40 mm, thickness > 80 mm	Integrated unit: 483 × 762 × 1092 mm	87
Laser	6.25 ~ 12.5 mJ cm^{-2} @ 1064 nm<br>20 mJ cm^{-2} @ 1064 nm	10 Hz	10 Hz (2D) ~1/15 Hz (3D)	0	Ring transducer array, Diameter = 220 mm, Elevation size = 5 mm	Optical head: 1173 × 508 × 120 mm Power supply: 770 × 640 × 757 mm	9,126
Laser	10 mJ cm^{-2} @ 610 nm	10 Hz	10 Hz (2D)	0	Ring transducer array, Diameter = 100 mm, Elevation size = 20 mm	Not described.	88
Laser	2 mJ cm^{-2} @ 630 nm<br>2 mJ cm^{-2} @ 780 nm<br>30 mJ cm^{-2} @ 780 nm	20 Hz	20 Hz (2D)	0	Ring transducer array, Diameter = 100 mm, Elevation size = 20 mm	Optical head: 800 × 450 × 150 mm Power supply: 446 × 449 × 177 mm Cooling system: 446 × 449 × 266 mm Control unit: 446 × 449 × 133 mm	89,127
Laser	125 mJ cm^{-2} @ 532 nm	10 Hz	10 Hz (2D)	0	ATL L7-4, commercial linear probe	Optical head: 147 × 526 × 125 mm Harmonic modules: 99 × 123 × 125 mm Power supply: 513 × 507 × 283 mm	128
Laser	12.7 mJ cm^{-2} @ 1064 nm	20 Hz	20 Hz (2D)	0	Ring transducer array, Diameter = 80 mm, Elevation size = 14 mm	Optical head: 735 × 179 × 162 mm Power supply: 647 × 334 × 554 mm	129
Laser	5 mJ cm^{-2} @ 760 nm	20 Hz	1 Hz (2D)	10	Ring transducer array, Diameter = 100 mm	Not described.	90
LED	10 µJ cm^{-2} @ 850 nm<br>2 µJ cm^{-2} @ 690 nm	1~4 kHz	0.15~30 Hz (2D)	32~25600	LEDs and ultrasound probe integrated.<br>Ultrasound probe: Linear array, ~ 40 × 10 × 80 mm<br>Optical head: 12.4 × 86.5 × 10.2 mm × 2<br>LED driver: -		91
LED	< 200 µJ @ 850 nm<br>< 80 µJ @ 690 nm	4 kHz, 16 kHz	10 Hz, 500 Hz (2D)	384 / 32	LEDs and ultrasound probe integrated.<br>Ultrasound probe: Linear array, ~ 40 × 10 × 80 mm<br>Optical head: 12.4 × 86.5 × 10.2 mm × 2<br>LED driver: -		38
LED	1.8 µJ @ 460 nm; 0.4 µJ @ 530 nm<br>1.7 µJ @ 590 nm; 2.7 µJ @ 620 nm<br>9 µJ @ 623nm	500 Hz	0.05 Hz 0.1 Hz (2D)	5000 / 10000	Cylindrical single transducer, Diameter = 10 mm Length: -	Optical head (LED): 11 × 10 × 5.3 mm<br>9 × 9 × 5.4 mm<br>LED driver: 51 × 82.5 × 13 mm	92
Laser diode	24 µJ / 184 µJ @ 905 nm	20 kHz / 2 kHz	4 Hz / 0.4 Hz (2D)	5000	Not described.	Not described.	31

Laser diode	0.56 mJ (1.5 mJ cm^{-2}) @ 805 nm	210 Hz	10 Hz (2D)	20	LEDs and ultrasound probe integrated. Size not mentioned. Larger than a commercial linear probe.		93
Laser diode	1.4 mJ (0.28 mJ cm^{-2}) @ 803 nm	7 kHz	1/3 Hz (2D)	42	Cylindrical single transducer, Diameter = 10 mm Length: -	Optical head: 137 × 76 × 38 mm	94
**Laser diode**	**~192 μJ @ 850 nm**	**3 kHz**	**1 Hz (2D, offline 3D)**	**3000**	**Laser diodes and ultrasound transducer integrated. Overall footprint: 20 × 16 × 1.2 mm**		**This work**

Supplementary Table 2 | Comparison between the soft photoacoustic patch in this work and other photoacoustic systems. Specifications of typical photoacoustic tomography systems include laser source type, pulse energy, pulse repetition rate, imaging frame rate, number of data averaging, size of ultrasound probe, and size of laser source. In the literature, PAT systems equipped with high-power lasers usually describe the laser energy per area on the tissue surface, while low-cost PAT systems using laser diodes or LEDs usually describe the pulse energy. The maximum pulse energy for the first kind is in the range from tens of millijoules to several joules by referring to the datasheets of the lasers.

	Wearable	Long-term continuous	Response time	Depth	Spatial mapping	Citations
**Magnetic Resonance Imaging (MRI)**	No	No	<1 s	>10 cm	Yes	[53]
**Resistance temperature detectors and thermistors**	Yes	Yes	~1 s	Skin surface	Yes	[50-52]
**Zero Heat flux model**	Yes	Yes	~180 s	~1 cm	No	[58]
**Dual Heat flux model**	Yes	Yes	>447 s	9.2 mm	No	[27]
**Photoacoustic patch**	Yes	Yes	~1 s	>2 cm	Yes	This work

Supplementary Table 3 | Comparison of different noninvasive temperature measurement techniques. The photoacoustic patch can noninvasively map the core temperature with high accuracy and fast response.

Equipment name	Company	model
Ultrasound Research platform	Verasonics	Vantage-256
Ultrasound transducer connector	ATL	Customized
Adapter	Verasonics	UTA-260D
Signal generator	Rigol	DG822
Power Supply	Agilent	E3620A
Laser diode driver	PicoLAS	LDP-V 240-100 V3.3
Thermometer	Omega Engineering	HH806AU
Thermocouple	Omega Engineering	SC-TT-K-30-36
ACF cable	Elform	Customized
Micro-coaxial cable	I-PEX	Customized
PCB convert board	JLC	Customized

Supplementary Table 4 | Equipment name, company name, and model of equipment in the experiments.

Material name	Company	Model
VCSEL	Ace Photonics	850nm VCSEL
Piezoelectric material	Del Piezo	1-3 PZT-5H
Ecoflex	Smooth On	00-30
Siver-Epoxy	Von Roll	3022
Copper foil	Oak-Mitsui Inc.	N/A
Polyimide	HD Microsystem	P12535
Aluminum Nitride	MARUWA	Customized
Gold	VEM	Gold sputtering target

Supplementary Table 5 | Material name, company, and model of the materials used in the experiments.

References

1 Uecker, M. *et al.* Real-time MRI at a resolution of 20 ms. *NMR Biomed.* **23**, 986-994 (2010).

2 Berger, A. How does it work?: Magnetic resonance imaging. *BMJ: British Medical Journal* **324**, 35 (2002).

3 Gambhir, S. S. Molecular imaging of cancer with positron emission tomography. *Nature Reviews Cancer* **2**, 683-693 (2002).

4 Berger, A. How does it work?: Positron emission tomography. *BMJ: British Medical Journal* **326**, 1449 (2003).

5 Diao, S. *et al.* Fluorescence imaging in vivo at wavelengths beyond 1500 nm. *Angew. Chem.* **127**, 14971-14975 (2015).

6 Huang, D. *et al.* Optical coherence tomography. *Science* **254**, 1178-1181 (1991).

7 Robles, F. E., Wilson, C., Grant, G. & Wax, A. Molecular imaging true-colour spectroscopic optical coherence tomography. *Nat. Photonics* **5**, 744-747 (2011).

8 Wang, L. V. & Hu, S. Photoacoustic tomography: in vivo imaging from organelles to organs. *science* **335**, 1458-1462 (2012).

9 Lin, L. *et al.* Single-breath-hold photoacoustic computed tomography of the breast. *Nat. Commun.* **9**, 1-9 (2018).

10 Li, L. *et al.* Single-impulse panoramic photoacoustic computed tomography of small-animal whole-body dynamics at high spatiotemporal resolution. *Nat. Biomed. Eng.* **1**, 1-11 (2017).

11 Gao, W. *et al.* Fully integrated wearable sensor arrays for multiplexed in situ perspiration analysis. *Nature* **529**, 509-514 (2016).

12 Lee, G.-H. *et al.* Multifunctional materials for implantable and wearable photonic healthcare devices. *Nature Reviews Materials* **5**, 149-165 (2020).

13 Lee, H. *et al.* Wearable/disposable sweat-based glucose monitoring device with multistage transdermal drug delivery module. *Science advances* **3**, e1601314 (2017).

14 Choi, J., Ghaffari, R., Baker, L. B. & Rogers, J. A. Skin-interfaced systems for sweat collection and analytics. *Science advances* **4**, eaar3921 (2018).

15 Bariya, M., Nyein, H. Y. Y. & Javey, A. Wearable sweat sensors. *Nature Electronics* **1**, 160-171 (2018).

16 Arakawa, T. *et al.* Mouthguard biosensor with telemetry system for monitoring of saliva glucose: A novel cavitas sensor. *Biosensors and Bioelectronics* **84**, 106-111 (2016).

17 Kim, J. *et al.* Wearable salivary uric acid mouthguard biosensor with integrated wireless electronics. *Biosensors and Bioelectronics* **74**, 1061-1068 (2015).

18 Reid, R. C., Minteer, S. D. & Gale, B. K. Contact lens biofuel cell tested in a synthetic tear solution. *Biosensors and Bioelectronics* **68**, 142-148 (2015).

19 Park, J. *et al.* Soft, smart contact lenses with integrations of wireless circuits, glucose sensors, and displays. *Science advances* **4**, eaap9841 (2018).

20 Ku, M. *et al.* Smart, soft contact lens for wireless immunosensing of cortisol. *Science advances* **6**, eabb2891 (2020).

21 Wang, Z. *et al.* Microneedle patch for the ultrasensitive quantification of protein biomarkers in interstitial fluid. *Nature Biomedical Engineering* **5**, 64-76 (2021).

22 Yang, B., Fang, X. & Kong, J. In situ sampling and monitoring cell-free dna of the epstein–barr virus from dermal interstitial fluid using wearable microneedle patches. *ACS applied materials & interfaces* **11**, 38448-38458 (2019).
23 Boyne, M. S., Silver, D. M., Kaplan, J. & Saudek, C. D. Timing of changes in interstitial and venous blood glucose measured with a continuous subcutaneous glucose sensor. *Diabetes* **52**, 2790-2794 (2003).
24 Hauke, A. *et al.* Complete validation of a continuous and blood-correlated sweat biosensing device with integrated sweat stimulation. *Lab on a Chip* **18**, 3750-3759 (2018).
25 Fallis, W. M. Monitoring urinary bladder temperature in the intensive care unit: state of the science. *American Journal of Critical Care* **11**, 38-45 (2002).
26 Krizanac, D. *et al.* Femoro-iliacal artery versus pulmonary artery core temperature measurement during therapeutic hypothermia: an observational study. *Resuscitation* **84**, 805-809 (2013).
27 Zhang, Y. *et al.* Theoretical and experimental studies of epidermal heat flux sensors for measurements of core body temperature. *Advanced healthcare materials* **5**, 119-127 (2016).
28 Teunissen, L., Klewer, J., De Haan, A., De Koning, J. & Daanen, H. Non-invasive continuous core temperature measurement by zero heat flux. *Physiological measurement* **32**, 559 (2011).
29 Liu, S. *et al.* Toward wearable healthcare: A miniaturized 3d imager with coherent frequency-domain photoacoustics. *IEEE transactions on biomedical circuits and systems* **13**, 1417-1424 (2019).
30 Kalva, S. K., Upputuri, P. K. & Pramanik, M. High-speed, low-cost, pulsed-laser-diode-based second-generation desktop photoacoustic tomography system. *Optics Letters* **44**, 81-84 (2019).
31 Allen, T. J. & Beard, P. C. Pulsed near-infrared laser diode excitation system for biomedical photoacoustic imaging. *Optics letters* **31**, 3462-3464 (2006).
32 Singh, M. K. A. & Steenbergen, W. Photoacoustic-guided focused ultrasound (PAFUSion) for identifying reflection artifacts in photoacoustic imaging. *Photoacoustics* **3**, 123-131 (2015).
33 Xia, W. *et al.* Handheld real-time LED-based photoacoustic and ultrasound imaging system for accurate visualization of clinical metal needles and superficial vasculature to guide minimally invasive procedures. *Sensors* **18**, 1394 (2018).
34 Jo, J. *et al.* Detecting joint inflammation by an LED-based photoacoustic imaging system: a feasibility study. *Journal of biomedical optics* **23**, 110501 (2018).
35 Zhang, H. F., Maslov, K., Stoica, G. & Wang, L. V. Functional photoacoustic microscopy for high-resolution and noninvasive in vivo imaging. *Nature biotechnology* **24**, 848-851 (2006).
36 Yao, J. *et al.* High-speed label-free functional photoacoustic microscopy of mouse brain in action. *Nat. Methods* **12**, 407-410 (2015).
37 Liu, S. *et al.* Handheld photoacoustic imager for theranostics in 3D. *IEEE Trans. Med. Imaging* **38**, 2037-2046 (2019).
38 Zhu, Y. *et al.* Light emitting diodes based photoacoustic imaging and potential clinical applications. *Scientific reports* **8**, 1-12 (2018).
39 Manwar, R., Zafar, M. & Xu, Q. Signal and Image Processing in Biomedical Photoacoustic Imaging: A Review. *Optics* **2**, 1-24 (2021).

40 Singh, M. K. A., Sato, N., Ichihashi, F. & Sankai, Y. LED-Based Photoacoustic Imaging. *Springer* **10**, 978-981 (2020).
41 Smith, A. M., Mancini, M. C. & Nie, S. Second window for in vivo imaging. *Nature nanotechnology* **4**, 710-711 (2009).
42 Sordillo, L. A., Pu, Y., Pratavieira, S., Budansky, Y. & Alfano, R. R. Deep optical imaging of tissue using the second and third near-infrared spectral windows. *Journal of biomedical optics* **19**, 056004 (2014).
43 Upputuri, P. K. & Pramanik, M. Photoacoustic imaging in the second near-infrared window: a review. *J. Biomed. Opt.* **24**, 040901 (2019).
44 Chen, Y.-S., Zhao, Y., Yoon, S. J., Gambhir, S. S. & Emelianov, S. Miniature gold nanorods for photoacoustic molecular imaging in the second near-infrared optical window. *Nat. Nanotechnol.* **14**, 465-472 (2019).
45 Lyu, Y., Li, J. & Pu, K. Second Near‐Infrared Absorbing Agents for Photoacoustic Imaging and Photothermal Therapy. *Small Methods* **3**, 1900553 (2019).
46 Sordillo, L. A. *et al.* in *Optical Biopsy XII.* 89400V (International Society for Optics and Photonics).
47 Fulbrook, P. Core body temperature measurement: a comparison of axilla, tympanic membrane and pulmonary artery blood temperature. *Intensive and Critical Care Nursing* **13**, 266-272 (1997).
48 Shi, C. *et al.* Application of a sub–0.1-mm3 implantable mote for in vivo real-time wireless temperature sensing. *Sci. Adv.* **7**, eabf6312 (2021).
49 Shin, J. *et al.* Bioresorbable optical sensor systems for monitoring of intracranial pressure and temperature. *Sci. Adv.* **5**, eaaw1899 (2019).
50 Han, S. *et al.* Battery-free, wireless sensors for full-body pressure and temperature mapping. *Sci. Transl. Med.* **10** (2018).
51 You, I. *et al.* Artificial multimodal receptors based on ion relaxation dynamics. *Science* **370**, 961-965 (2020).
52 *Thermocouple Response Time*, <https://www.omega.com/en-us/resources/thermocouples-response-time> (2019).
53 Denis de Senneville, B., Quesson, B. & Moonen, C. T. Magnetic resonance temperature imaging. *Int. J. Hyperthermia* **21**, 515-531 (2005).
54 Eshraghi, Y. *et al.* An evaluation of a zero-heat-flux cutaneous thermometer in cardiac surgical patients. *Anesthesia & Analgesia* **119**, 543-549 (2014).
55 Huang, M., Tamura, T., Tang, Z., Chen, W. & Kanaya, S. A wearable thermometry for core body temperature measurement and its experimental verification. *IEEE journal of biomedical and health informatics* **21**, 708-714 (2016).
56 Kitamura, K.-I., Zhu, X., Chen, W. & Nemoto, T. Development of a new method for the noninvasive measurement of deep body temperature without a heater. *Med. Eng. Phys.* **32**, 1-6 (2010).
57 Yamakage, M. & Namiki, A. Deep temperature monitoring using a zero-heat-flow method. *J. Anesth.* **17**, 108-115 (2003).
58 Pesonen, E. *et al.* The focus of temperature monitoring with zero-heat-flux technology (3M Bair-Hugger): a clinical study with patients undergoing craniotomy. *J. Clin. Monit. Comput.* **33**, 917-923 (2019).
59 Zhou, Y. *et al.* Thermal memory based photoacoustic imaging of temperature. *Optica* **6**, 198-205 (2019).

60 Shah, J. *et al.* Photoacoustic imaging and temperature measurement for photothermal cancer therapy. *J. Biomed. Opt.* **13**, 034024 (2008).
61 Alaeian, M. & Orlande, H. R. B. Inverse photoacoustic technique for parameter and temperature estimation in tissues. *Heat Transfer Engineering* **38**, 1573-1594 (2017).
62 Wu, X., Sanders, J. L., Stephens, D. N. & Oralkan, Ö. in *2016 38th Annual International Conference of the IEEE Engineering in Medicine and Biology Society (EMBC).* 3235-3238 (IEEE).
63 Duck, F. A. *Physical properties of tissues: a comprehensive reference book.* (Academic press, 2013).
64 Dewitte, K., Fierens, C., Stockl, D. & Thienpont, L. M. Application of the Bland–Altman plot for interpretation of method-comparison studies: a critical investigation of its practice. *Clinical chemistry* **48**, 799-801 (2002).
65 Lee, K. *et al.* Mechano-acoustic sensing of physiological processes and body motions via a soft wireless device placed at the suprasternal notch. *Nature biomedical engineering* **4**, 148-158 (2020).
66 Chung, H. U. *et al.* Skin-interfaced biosensors for advanced wireless physiological monitoring in neonatal and pediatric intensive-care units. *Nature medicine* **26**, 418-429 (2020).
67 Chung, H. U. *et al.* Binodal, wireless epidermal electronic systems with in-sensor analytics for neonatal intensive care. *Science* **363** (2019).
68 Song, Z., Ding, H., Li, J. & Pu, H. Circular curve-fitting method for field surveying data with correlated noise. *J. Surv. Eng.* **144**, 04018010 (2018).
69 Treeby, B. E. & Cox, B. T. k-Wave: MATLAB toolbox for the simulation and reconstruction of photoacoustic wave fields. *J. Biomed. Opt.* **15**, 021314 (2010).
70 Hai, P. *et al.* Label-free high-throughput photoacoustic tomography of suspected circulating melanoma tumor cells in patients in vivo. *Journal of biomedical optics* **25**, 036002 (2020).
71 Chaffer, C. L. & Weinberg, R. A. A perspective on cancer cell metastasis. *science* **331**, 1559-1564 (2011).
72 Wang, Y. *et al.* Fiber-laser-based photoacoustic microscopy and melanoma cell detection. *Journal of biomedical optics* **16**, 011014 (2011).
73 He, Y. *et al.* In vivo label-free photoacoustic flow cytography and on-the-spot laser killing of single circulating melanoma cells. *Scientific reports* **6**, 1-8 (2016).
74 Zhang, R. *et al.* Noninvasive photoacoustic measurement of glucose by data fusion. *Analyst* **142**, 2892-2896 (2017).
75 Pleitez, M. A. *et al.* In vivo noninvasive monitoring of glucose concentration in human epidermis by mid-infrared pulsed photoacoustic spectroscopy. *Analytical chemistry* **85**, 1013-1020 (2013).
76 Sim, J. Y., Ahn, C.-G., Jeong, E.-J. & Kim, B. K. In vivo microscopic photoacoustic spectroscopy for non-invasive glucose monitoring invulnerable to skin secretion products. *Scientific reports* **8**, 1-11 (2018).
77 Zhang, C., Zhang, Y. S., Yao, D.-K., Xia, Y. & Wang, L. V. Label-free photoacoustic microscopy of cytochromes. *Journal of biomedical optics* **18**, 020504 (2013).
78 Zhang, C., Zhang, Y., Yao, D.-K., Xia, Y. & Wang, L. V. in *Photons Plus Ultrasound: Imaging and Sensing 2012.* 82231W (International Society for Optics and Photonics).

79 Yang, L. *et al.* ICG‐Conjugated and 125I‐Labeled Polymeric Micelles with High Biosafety for Multimodality Imaging‐Guided Photothermal Therapy of Tumors. *Advanced healthcare materials* **9**, 1901616 (2020).
80 Alander, J. T. *et al.* A review of indocyanine green fluorescent imaging in surgery. *International journal of biomedical imaging* **2012** (2012).
81 Lutzweiler, C., Meier, R., Rummeny, E., Ntziachristos, V. & Razansky, D. Real-time optoacoustic tomography of indocyanine green perfusion and oxygenation parameters in human finger vasculature. *Optics letters* **39**, 4061-4064 (2014).
82 Stoffels, I. *et al.* Metastatic status of sentinel lymph nodes in melanoma determined noninvasively with multispectral optoacoustic imaging. *Science translational medicine* **7**, 317ra199-317ra199 (2015).
83 Stylogiannis, A. *et al.* Continuous wave laser diodes enable fast optoacoustic imaging. *Photoacoustics* **9**, 31-38 (2018).
84 Deán-Ben, X. L., Fehm, T. F., Ford, S. J., Gottschalk, S. & Razansky, D. Spiral volumetric optoacoustic tomography visualizes multi-scale dynamics in mice. *Light: Science & Applications* **6**, e16247-e16247 (2017).
85 Na, S. *et al.* Massively parallel functional photoacoustic computed tomography of the human brain. *Nature Biomedical Engineering* **6**, 584-592 (2022).
86 Wang, Y., Wang, D., Hubbell, R. & Xia, J. Second generation slit‐based photoacoustic tomography system for vascular imaging in human. *Journal of biophotonics* **10**, 799-804 (2017).
87 Ivankovic, I., Merčep, E., Schmedt, C.-G., Deán-Ben, X. L. & Razansky, D. Real-time volumetric assessment of the human carotid artery: handheld multispectral optoacoustic tomography. *Radiology* **291**, 45-50 (2019).
88 Li, L., Hsu, H. C., Verkhusha, V. V., Wang, L. V. & Shcherbakova, D. M. Multiscale Photoacoustic Tomography of a Genetically Encoded Near‐Infrared FRET Biosensor. *Advanced Science* **8**, 2102474 (2021).
89 Zhang, P., Li, L., Lin, L., Shi, J. & Wang, L. V. In vivo superresolution photoacoustic computed tomography by localization of single dyed droplets. *Light: Science & Applications* **8**, 1-9 (2019).
90 Yang, J. *et al.* Photoacoustic assessment of hemodynamic changes in foot vessels. *Journal of biophotonics* **12**, e201900004 (2019).
91 Hariri, A. *et al.* The characterization of an economic and portable LED-based photoacoustic imaging system to facilitate molecular imaging. *Photoacoustics* **9**, 10-20 (2018).
92 Allen, T. J. & Beard, P. C. High power visible light emitting diodes as pulsed excitation sources for biomedical photoacoustics. *Biomedical optics express* **7**, 1260-1270 (2016).
93 Daoudi, K. *et al.* Handheld probe integrating laser diode and ultrasound transducer array for ultrasound/photoacoustic dual modality imaging. *Optics express* **22**, 26365-26374 (2014).
94 Upputuri, P. K. & Pramanik, M. Performance characterization of low-cost, high-speed, portable pulsed laser diode photoacoustic tomography (PLD-PAT) system. *Biomedical optics express* **6**, 4118-4129 (2015).
95 Mienkina, M. P. *et al.* Multispectral photoacoustic coded excitation imaging using unipolar orthogonal Golay codes. *Optics Express* **18**, 9076-9087 (2010).

96 Su, S.-Y. & Li, P.-C. Coded excitation for photoacoustic imaging using a high-speed diode laser. *Optics express* **19**, 1174-1182 (2011).

97 Mienkina, M. P. *et al.* Experimental evaluation of photoacoustic coded excitation using unipolar golay codes. *IEEE transactions on ultrasonics, ferroelectrics, and frequency control* **57**, 1583-1593 (2010).

98 Zhang, H. K., Kondo, K., Yamakawa, M. & Shiina, T. Coded excitation using periodic and unipolar M-sequences for photoacoustic imaging and flow measurement. *Optics Express* **24**, 17-29 (2016).

99 Zhou, M., Xia, H., Zhong, H., Zhang, J. & Gao, F. A noise reduction method for photoacoustic imaging in vivo based on EMD and conditional mutual information. *IEEE Photonics Journal* **11**, 1-10 (2019).

100 Sun, M., Feng, N., Shen, Y., Shen, X. & Li, J. Photoacoustic signals denoising based on empirical mode decomposition and energy-window method. *Advances in Adaptive Data Analysis* **4**, 1250004 (2012).

101 Donoho, D. L. & Johnstone, J. M. Ideal spatial adaptation by wavelet shrinkage. *biometrika* **81**, 425-455 (1994).

102 Zhou, M. *et al.* in *2018 40th Annual International Conference of the IEEE Engineering in Medicine and Biology Society (EMBC).* 4796-4799 (IEEE).

103 Van de Sompel, D., Sasportas, L. S., Jokerst, J. V. & Gambhir, S. S. Comparison of deconvolution filters for photoacoustic tomography. *PloS one* **11**, e0152597 (2016).

104 Manwar, R. *et al.* Photoacoustic signal enhancement: towards utilization of low energy laser diodes in real-time photoacoustic imaging. *Sensors* **18**, 3498 (2018).

105 Nilsen, C.-I. C. & Holm, S. Wiener beamforming and the coherence factor in ultrasound imaging. *IEEE transactions on ultrasonics, ferroelectrics, and frequency control* **57**, 1329-1346 (2010).

106 Jeon, S. *et al.* Real-time delay-multiply-and-sum beamforming with coherence factor for in vivo clinical photoacoustic imaging of humans. *Photoacoustics* **15**, 100136 (2019).

107 Park, S., Karpiouk, A. B., Aglyamov, S. R. & Emelianov, S. Y. Adaptive beamforming for photoacoustic imaging. *Optics letters* **33**, 1291-1293 (2008).

108 Friedlein, J. T. *et al.* Dual-comb photoacoustic spectroscopy. *Nature communications* **11**, 1-10 (2020).

109 Telenkov, S. & Mandelis, A. Signal-to-noise analysis of biomedical photoacoustic measurements in time and frequency domains. *Review of Scientific Instruments* **81**, 124901 (2010).

110 Lengenfelder, B. *et al.* Remote photoacoustic sensing using speckle-analysis. *Scientific reports* **9**, 1-11 (2019).

111 Wang, Y. *et al.* In vivo three-dimensional photoacoustic imaging based on a clinical matrix array ultrasound probe. *Journal of Biomedical Optics* **17**, 061208 (2012).

112 Kothapalli, S.-R. *et al.* Deep tissue photoacoustic imaging using a miniaturized 2-D capacitive micromachined ultrasonic transducer array. *IEEE Transactions on Biomedical Engineering* **59**, 1199-1204 (2012).

113 Wang, L., Maslov, K. I., Xing, W., Garcia-Uribe, A. & Wang, L. V. Video-rate functional photoacoustic microscopy at depths. *Journal of biomedical optics* **17**, 106007 (2012).

114 Shi, J. *et al.* High-resolution, high-contrast mid-infrared imaging of fresh biological samples with ultraviolet-localized photoacoustic microscopy. *Nature photonics* **13**, 609-615 (2019).

115 Yang, J. *et al.* Motionless volumetric photoacoustic microscopy with spatially invariant resolution. *Nature communications* **8**, 1-7 (2017).
116 Shi, J., Wang, L., Noordam, C. & Wang, L. V. Bessel-beam Grueneisen relaxation photoacoustic microscopy with extended depth of field. *Journal of biomedical optics* **20**, 116002 (2015).
117 Wang, L., Maslov, K., Yao, J., Rao, B. & Wang, L. V. Fast voice-coil scanning optical-resolution photoacoustic microscopy. *Optics letters* **36**, 139-141 (2011).
118 Cebrecos, A. *et al.* Beamforming for large-area scan and improved SNR in array-based photoacoustic microscopy. *Ultrasonics* **111**, 106317 (2021).
119 Deán-Ben, X. L., López-Schier, H. & Razansky, D. Optoacoustic micro-tomography at 100 volumes per second. *Scientific reports* **7**, 1-8 (2017).
120 Paul, S., Mandal, S. & Singh, M. S. Noise adaptive beamforming for linear array photoacoustic imaging. *IEEE Transactions on Instrumentation and Measurement* **70**, 1-11 (2021).
121 Yao, J., Ke, H., Tai, S., Zhou, Y. & Wang, L. V. Absolute photoacoustic thermometry in deep tissue. *Optics letters* **38**, 5228-5231 (2013).
122 Liu, S., Feng, X., Ruochong, Z. & Zheng, Y. in *2018 IEEE International Symposium on Circuits and Systems (ISCAS).* 1-5 (IEEE).
123 Xiaohua, F., Fei, G. & Yuanjin, Z. Photoacoustic-based-close-loop temperature control for nanoparticle hyperthermia. *IEEE Transactions on Biomedical Engineering* **62**, 1728-1737 (2015).
124 Li, Y. L., Hyun, D., Abou-Elkacem, L., Willmann, J. K. & Dahl, J. J. Visualization of small-diameter vessels by reduction of incoherent reverberation with coherent flow power doppler. *IEEE transactions on ultrasonics, ferroelectrics, and frequency control* **63**, 1878-1889 (2016).
125 Instruments, T. *Ultrasound smart probe - Products and reference designs*, <https://www.ti.com/solution/ultrasound-smart-probe?variantid=35221> (2018).
126 Wray, P., Lin, L., Hu, P. & Wang, L. V. Photoacoustic computed tomography of human extremities. *Journal of biomedical optics* **24**, 026003 (2019).
127 Li, L. *et al.* Small near-infrared photochromic protein for photoacoustic multi-contrast imaging and detection of protein interactions in vivo. *Nature communications* **9**, 1-14 (2018).
128 Vu, T. *et al.* Photoacoustic computed tomography of mechanical HIFU-induced vascular injury. *Biomedical Optics Express* **12**, 5489-5498 (2021).
129 Zhang, Y. & Wang, L. Video-rate ring-array ultrasound and photoacoustic tomography. *IEEE Transactions on Medical Imaging* **39**, 4369-4375 (2020).
