# Supplementary material for: A photoacoustic patch for three-dimensional imaging of hemoglobin and core temperature
Source: Nat Commun. 2022 Dec 15;13:7757. doi: 10.1038/s41467-022-35455-3 (PMC9755152; doi:10.1038/s41467-022-35455-3)
Supplement: Supplementary file 2 — Description of Additional Supplementary Files [file 41467_2022_35455_MOESM2_ESM.docx]

**Description of Additional Supplementary Files**

**Supplementary Movie 1:**

Real time imaging of venous occlusion test on the human body shown in MATLAB. The test includes three steps: no pressure applied, pressure applied, and cuff released.
