# Supplementary material for: A photoacoustic patch for three-dimensional imaging of hemoglobin and core temperature
Source: Nat Commun. 2022 Dec 15;13:7757. doi: 10.1038/s41467-022-35455-3 (PMC9755152; doi:10.1038/s41467-022-35455-3)
Supplement: Supplementary file 4 — Reporting Summary [file 41467_2022_35455_MOESM4_ESM.pdf]

nature portfolio

Corresponding author(s): Sheng Xu

Last updated by author(s): Oct 31, 2022

Reporting Summary

Nature Portfolio wishes to improve the reproducibility of the work that we publish. This form provides structure for consistency and transparency in reporting. For further information on Nature Portfolio policies, see our Editorial Policies and the Editorial Policy Checklist.

Statistics

For all statistical analyses, confirm that the following items are present in the figure legend, table legend, main text, or Methods section.

n/a	Confirmed	
☐	☒	The exact sample size (*n*) for each experimental group/condition, given as a discrete number and unit of measurement
☐	☒	A statement on whether measurements were taken from distinct samples or whether the same sample was measured repeatedly
☒	☐	The statistical test(s) used AND whether they are one- or two-sided<br>*Only common tests should be described solely by name; describe more complex techniques in the Methods section.*
☒	☐	A description of all covariates tested
☒	☐	A description of any assumptions or corrections, such as tests of normality and adjustment for multiple comparisons
☐	☒	A full description of the statistical parameters including central tendency (e.g. means) or other basic estimates (e.g. regression coefficient) AND variation (e.g. standard deviation) or associated estimates of uncertainty (e.g. confidence intervals)
☒	☐	For null hypothesis testing, the test statistic (e.g. *F*, *t*, *r*) with confidence intervals, effect sizes, degrees of freedom and *P* value noted<br>*Give P values as exact values whenever suitable.*
☒	☐	For Bayesian analysis, information on the choice of priors and Markov chain Monte Carlo settings
☒	☐	For hierarchical and complex designs, identification of the appropriate level for tests and full reporting of outcomes
☒	☐	Estimates of effect sizes (e.g. Cohen's *d*, Pearson's *r*), indicating how they were calculated

Our web collection on statistics for biologists contains articles on many of the points above.

Software and code

Policy information about availability of computer code

Data collection	The data is collected by commercial software Matlab 2018a.
Data analysis	The data is analyzed in commercial software Matlab 2018a. 2D image is constructed based on coherence factor weighted delay and sum algorithm in published literature. The 3D image is constructed in Amira 2019.1. The figures are drawn in OriginPro 9.0.

For manuscripts utilizing custom algorithms or software that are central to the research but not yet described in published literature, software must be made available to editors and reviewers. We strongly encourage code deposition in a community repository (e.g. GitHub). See the Nature Portfolio guidelines for submitting code & software for further information.

Data

Policy information about availability of data

All manuscripts must include a data availability statement. This statement should provide the following information, where applicable:

- Accession codes, unique identifiers, or web links for publicly available datasets
- A description of any restrictions on data availability
- For clinical datasets or third party data, please ensure that the statement adheres to our policy

All data supporting the findings of this study are available within the paper and its Supplementary Information. The data generated in this study are available from a public data repository at https://figshare.com/articles/dataset/PA_patch/21440925.

Human research participants

Policy information about studies involving human research participants and Sex and Gender in Research.

Reporting on sex and gender	In our research, we only recruited one volunteer (male) for experiments. Gender was not a factor to be considered in the experiment design. Gender was determined based on self-reporting. Our experiments were mainly about the blood vessel imaging of human body. Gender was not a variable that affects the results of the experiment.
Population characteristics	The volunteer is a 25 year old man. He is healthy without any diseases.
Recruitment	We posted recruitment flyers in the office building to recruit the volunteer. The volunteer contacted us by himself. He was not selected from our own group or any group that has partnership with us.
Ethics oversight	University of California, San Diego Institutional Review Board

Note that full information on the approval of the study protocol must also be provided in the manuscript.

Field-specific reporting

Please select the one below that is the best fit for your research. If you are not sure, read the appropriate sections before making your selection.

[x] Life sciences [ ] Behavioural & social sciences [ ] Ecological, evolutionary & environmental sciences

For a reference copy of the document with all sections, see nature.com/documents/nr-reporting-summary-flat.pdf

Life sciences study design

All studies must disclose on these points even when the disclosure is negative.

Sample size	We only recruited one volunteer in this research. This research is not about clinical applications. It only demonstrates a proof-of-concept of a device. No statistical methods used in this research.
Data exclusions	No data was excluded from analysis.
Replication	All of the measurements were taken on the same subject for multiple times (with the stable physiological condition). The data at each test location were sampled for multiple times (more than 5 times) and compared during the measurements. From the measured results, the data showed high similarity, and the reproducibility of all kinds of measurements were good in this research.
Randomization	The subject was recruited from outside the group by posting flyers. He was randomly selected.
Blinding	The subject was only told the experimental process. The subject did not know test results. The subject did not participate in data processing.

Reporting for specific materials, systems and methods

We require information from authors about some types of materials, experimental systems and methods used in many studies. Here, indicate whether each material, system or method listed is relevant to your study. If you are not sure if a list item applies to your research, read the appropriate section before selecting a response.

Materials & experimental systems

n/a	Involved in the study	
[x]	[ ]	Antibodies
[x]	[ ]	Eukaryotic cell lines
[x]	[ ]	Palaeontology and archaeology
[x]	[ ]	Animals and other organisms
[x]	[ ]	Clinical data
[x]	[ ]	Dual use research of concern

Methods

n/a	Involved in the study	
[x]	[ ]	ChIP-seq
[x]	[ ]	Flow cytometry
[x]	[ ]	MRI-based neuroimaging
